# High diversity, inbreeding and a dynamic Pleistocene demographic history revealed by African buffalo genomes

**DOI:** 10.1038/s41598-021-83823-8

**Published:** 2021-02-25

**Authors:** Deon de Jager, Brigitte Glanzmann, Marlo Möller, Eileen Hoal, Paul van Helden, Cindy Harper, Paulette Bloomer

**Affiliations:** 1 Molecular Ecology and Evolution Programme, Department of Biochemistry, Genetics and Microbiology, Faculty of Natural and Agricultural Sciences, University of Pretoria, Pretoria, South Africa; 2 DSI-NRF Centre of Excellence for Biomedical Tuberculosis Research, Stellenbosch University, Cape Town, South Africa; 3 South African Medical Research Council Centre for Tuberculosis Research, Stellenbosch University, Cape Town, South Africa; 4 Division of Molecular Biology and Human Genetics, Faculty of Medicine and Health Sciences, Stellenbosch University, Cape Town, South Africa; 5 Veterinary Genetics Laboratory, Faculty of Veterinary Science, University of Pretoria, Pretoria, South Africa

**Keywords:** Population genetics, Genetic variation, Conservation genomics, Inbreeding

## Abstract

Genomes retain records of demographic changes and evolutionary forces that shape species and populations. Remnant populations of African buffalo (*Syncerus caffer*) in South Africa, with varied histories, provide an opportunity to investigate signatures left in their genomes by past events, both recent and ancient. Here, we produce 40 low coverage (7.14×) genome sequences of Cape buffalo (*S. c. caffer*) from four protected areas in South Africa. Genome-wide heterozygosity was the highest for any mammal for which these data are available, while differences in individual inbreeding coefficients reflected the severity of historical bottlenecks and current census sizes in each population. PSMC analysis revealed multiple changes in *N*_e_ between approximately one million and 20 thousand years ago, corresponding to paleoclimatic changes and Cape buffalo colonisation of southern Africa. The results of this study have implications for buffalo management and conservation, particularly in the context of the predicted increase in aridity and temperature in southern Africa over the next century as a result of climate change.

## Introduction

Population genomics studies of non-model organisms have increased considerably in recent years, owing to the development of reduced-representation sequencing methods and the ever-decreasing cost of short-read sequencing^1^. Often, researchers need to use the genome of a closely, or sometimes distantly, related species as reference. Consequently, ascertainment bias and reduced efficiency of read mapping results in large amounts of unused sequence data. For example, in studies of African buffalo (*Syncerus caffer*) where the cattle (*Bos taurus*) genome has typically been used as the reference, only 19–30% of reads mapped to the reference genome^2,3^. Fortunately, it has become possible to sequence whole genomes of non-model species at high coverage, thus creating valuable species-specific genomic resources^4^.

The African buffalo, a large, gregarious ruminant, is classified as Near Threatened on the International Union for Conservation of Nature (IUCN) Red List of Threatened Species, owing mainly to a decreasing population trend^5^. This trend is predicted to continue and likely accelerate, predominantly due to human population expansion and related activities, such as development, agriculture and biological resource use^5^. The species has high conservation value, as it is the only extant member of its genus and is both ecologically (disease reservoir, herbivory, prey) and economically (eco- and consumptive tourism, wildlife ranching) important, particularly in South Africa^6–9^. For the Cape buffalo subspecies (*S. c. caffer*), a 14% decline was observed between 1998 (> 548,000 individuals) and 2014 (> 473,000) across its range^5,10^. The majority of this decline occurred in Tanzania, where > 342,000 buffalo in 1998 decreased to > 189,230 individuals in 2014 (− 45%)^10^. Most other range countries showed more stable population sizes over this period, while the southern African countries of Botswana, Namibia, Mozambique, Zimbabwe and South Africa showed increases in buffalo numbers^10^. In South Africa, Cape buffalo increased from ~ 30,970 to > 77,800 between 1998 and 2014^10^. This dramatic increase was predominantly a result of the disease-free breeding programme of South African National Parks (SANParks) and the expansion of the wildlife ranching industry, where ~ 26,000 disease-free buffalo occur on ~ 2700 privately-owned game ranches or reserves^10^. However, protected areas in South Africa are highly fragmented, as are private ranches, and gene flow between populations is contingent on the translocation of buffalo, which is often precluded by regulations to prevent the spread of bovid diseases, such as foot-and-mouth disease and bovine tuberculosis, of which buffalo are carriers^11^.

Population genetics studies of African buffalo, employing microsatellites and mitochondrial DNA, have found high levels of differentiation between subspecies^12–14^ and between populations of the Cape buffalo subspecies (*S. c. caffer*) across Africa^13,15–17^. The few buffalo studies employing more modern genetics techniques have focused on disease-related variants and variant discovery, while relying on the cattle genome as reference^2,3,18,19^.

Disease has played a significant role in shaping buffalo populations in southern Africa. Only an estimated 5% of buffalo in the region survived the rinderpest and foot-and-mouth disease epidemics of the late 1890s and early 1900s^11^. Furthermore, in the early to mid-1900s, wild ungulates that are hosts of the tsetse fly (*Glossina* spp.), including buffalo, were subject to large-scale extermination efforts in southern Africa to eradicate the tsetse fly, which transmitted sleeping sickness to humans and trypanosomiasis (nagana) to cattle^11,20^. After this period, what remained of naturally occurring South African buffalo populations was represented by relict populations in three important protected areas, geographically isolated from each other, namely the Kruger National Park (KNP), Hluhluwe-iMfolozi Park (HiP) and Addo Elephant National Park (AENP).

In the north-east of South Africa, KNP has the largest free-ranging buffalo population in the country, with a census of ~ 40,900 individuals in 2011^10^. Using a maximum likelihood method based on genetic data, O'Ryan et al.^16^ found that the minimum population size of KNP was likely not lower than 1600 individuals between 1898 (the year KNP was proclaimed as a protected area under the name Sabi Game Reserve) and 1998. This population maintains the highest genetic diversity of natural populations in South Africa (expected heterozygosity based on microsatellites [*H*_E_] of 0.62–0.75)^13,15,16,21,22^ and is thus used as the reference point to which all other local populations are compared^15,16^. Currently, buffalo in KNP harbour several economically important diseases, namely foot-and-mouth disease, corridor disease, brucellosis and bovine tuberculosis^23^. Buffalo in KNP cluster genetically with populations in the neighbouring countries of Zimbabwe and Mozambique, due to historical connections and present-day open borders within the Greater Limpopo Transfrontier Park and some cross-border gene flow likely occurs between populations within this transfrontier park^2^.

Following outbreaks of bovine tuberculosis and corridor disease in the 1980s and 1990s, South African National Parks recognized the need to preserve the genetic diversity of KNP buffalo in a population outside the disease control zone- a so-called disease-free population^24^. Such a population was established in Mokala National Park (MNP) in the centre of South Africa near Kimberley in the Northern Cape Province in 1999 through a disease-free breeding programme (*Pers. Comm.* D. Zimmerman 2015)^17^. The breeding programme consisted of buffalo (approximately 140 cows and 10 bulls) that originated mainly from northern KNP that were contained in a fenced-off camp in KNP (*Pers. Comm.* D. Zimmerman 2015). From 1999 onwards, the MNP population was supplemented with yearlings from this breeding group, after disease testing, until 2007 (*Pers. Comm.* D. Zimmerman 2015). No buffalo have been introduced to MNP since 2007 (*Pers. Comm.* D. Zimmerman 2015). The current census size of MNP is approximately 400 buffalo (*Pers. Comm.* D. Zimmerman 2015). This population has been shown to have high genetic diversity based on microsatellite data (*H*_E_ = 0.61), indicating the disease-free breeding programme likely achieved its goal of preserving the high diversity of the KNP buffalo population, although a direct comparison between MNP and KNP was not performed^17^.

Hluhluwe-iMfolozi Park (HiP), situated in the KwaZulu-Natal Province in the east of the country, harbours the second-largest free-ranging buffalo population in South Africa^10^. The most recent census survey estimated a population size of 4544 in HiP^25^. HiP had approximately 8400 buffalo in 1998^16^. The census size of buffalo in HiP at the time of its proclamation (as a protected area) in 1895 is unknown, but the earliest known estimate was 75 buffalo in 1929^16^. Based on their maximum likelihood method O'Ryan et al.^16^ estimated a population size of ~ 429 buffalo for HiP in the period between 1929 and 1977. Currently the HiP population harbours corridor disease and bovine tuberculosis^23^, the latter of which is actively managed through a test-and-cull programme. The buffalo population in HiP has been shown to have lower genetic diversity (*H*_E_ = 0.533–0.549)^15,16^ than KNP, but higher diversity than AENP^15,16^. There have been no recorded introductions of buffalo to HiP since its proclamation as a protected area.

Addo Elephant National Park (AENP), is situated in the Eastern Cape Province on the southern coast of South Africa. A recent census of AENP buffalo estimated a population size of ~ 800 individuals (*Pers. Comm.* D. Zimmerman 2015). The size of the buffalo population in AENP was not known at the time it was proclaimed in 1931. However, 130 buffalo were removed in 1981, reducing the population to 75 individuals. By 1983 the population had increased to approximately 220 individuals, but was reduced again to 52 buffalo in 1985, potentially due to drought during that time (*Pers. Comm.* D. Zimmerman 2015)^17^. There are no records of any human-mediated introduction of buffalo to AENP at any point in the history of this population (*Pers. Comm.* D. Zimmerman 2015). AENP has the lowest genetic diversity of the three relict populations of buffalo in South Africa (*H*_E_ = 0.425 – 0.482)^16,17^, being lower than both KNP and HiP^16^. In contrast to KNP and HiP, the buffalo populations in AENP and MNP currently harbour no diseases of economic importance and AENP has historically also been disease-free^23^, presumably since the disease outbreaks of the 1890s and 1900s.

In this study, we used the recently published Cape buffalo (*S. c. caffer*) reference genome^26^ to conduct the first population genomics study of this species. We generated 40 low coverage genomes from four protected areas in South Africa, namely KNP, MNP, HiP and AENP. We aimed to (i) Investigate whether known recent population bottlenecks resulted in low genome-wide diversity and/or high individual inbreeding coefficients, (ii) Determine whether the disease-free breeding programme maintained high genetic diversity in MNP, by direct comparison with its source population, KNP, and (iii) Determine whether genome-wide data mirror the results of previous microsatellites studies regarding the differentiation between these populations. While microsatellites and mitogenomes have been used to investigate Holocene and Late Pleistocene buffalo demographic history in East Africa^27,28^, we take advantage of the increased power of these whole-genome sequences to (iv) Elucidate the more ancient Pleistocene demography of the species, while also determining whether the genome-wide data support previously estimated expansion events. We discuss the conservation and management implications of the findings based on these population genomic data throughout.

## Methods

### Samples and DNA extraction

**Figure 1:**
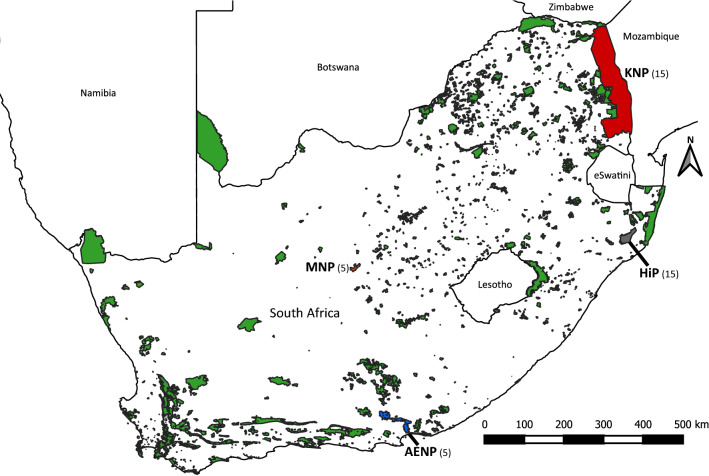
Sampling localities of buffalo included in this study. Sampling localities are shown in colours other than green. Sample sizes are indicated in parentheses. Green polygons show all other terrestrial protected areas in South Africa. The protected area shapefile was downloaded from https://egis.environment.gov.za/protected_and_conservation_areas_database, accessed on 10/08/2020. The map was created in QGIS v3.4^29^ (https://www.qgis.org/en/site/), with labels and colours for sampling localities added in Inkscape v0.92^30^ (https://www.inkscape.org).

Sampling locations are shown in Fig. 1, together with all terrestrial protected areas in South Africa. The protected area shapefile was downloaded from https://egis.environment.gov.za/protected_and_conservation_areas_database, accessed on 10/08/2020. The map was constructed in QGIS v3.4^29^. Buffalo blood samples stored in ethylenediaminetetraacetic acid (EDTA) were previously obtained from South African National Parks (SANParks) and Ezemvelo KZN Wildlife (EKZNW) for unrelated studies and their use in this study was incidental. The authors declare that the ethical standards required in terms of the University of Pretoria’s Code of ethics for researchers and the Policy guidelines for responsible research were observed. The applicable research ethics approval for experimental protocols was obtained from the Animal Ethics Committee of the University of Pretoria and all experiments were performed in accordance with relevant guidelines and regulations. Five unrelated samples from each of AENP and MNP were contributed by the Veterinary Genetics Laboratory, University of Pretoria. Fifteen samples from each of KNP and HiP were contributed by the Division of Molecular Biology and Human Genetics, Stellenbosch University. High molecular weight (HMW) DNA was extracted from the AENP and MNP samples using the MagAttract HMW DNA Mini Kit (Qiagen, Hilden, Germany), according to the manufacturer’s instructions. HMW DNA was previously extracted from the KNP and HiP samples using the illustra Nucleon BACC3 RPN8512 kit (GE Healthcare Life Sciences, Chicago, IL, United States of America), according to the manufacturer’s instructions.

### Genome resequencing

Low coverage whole-genome sequencing of the 40 buffalo samples was outsourced to Novogene (Beijing, People’s Republic of China). In short, a total of 1.0 μg DNA per sample was used for DNA sample preparations. Sequencing libraries were generated using the Truseq Nano DNA HT Sample Preparation Kit (Illumina, San Diego, CA, USA) following the manufacturer's instructions. Index codes were added to attribute sequences to each sample. Genomic DNA was randomly fragmented to a size of 350 base pairs (bp) using a Covaris sonicator (Covaris, Woburn, MA, USA). Thereafter, DNA fragments were end polished, A-tailed, and ligated with the full-length adapter for Illumina sequencing with further polymerase chain reaction (PCR) amplification. PCR products were subsequently purified using the AMPure XP system (Beckman Coulter, Brea, CA, USA). Finally, the size distribution of the libraries was analysed using the Agilent 2100 Bioanalyzer (Agilent, Santa Clara, CA, USA) and quantified using real-time PCR. The libraries were then sequenced as 150 bp paired-end reads on a HiSeq X instrument (Illumina).

### Quality control of raw sequences

FastQC v0.11.5^31^ was used to evaluate the quality of the raw sequences, adapter contamination and overrepresented sequences. Trimmomatic v0.36^32^ was used to filter low quality reads and remove adapter sequences. Commonly used Illumina adapter sequences, provided with Trimmomatic, were used to identify adapter contamination via the palindrome mode, allowing two seed mismatches, a palindrome clip threshold of 30, a simple clip threshold of 10 and a minimum adapter length to be clipped of one nucleotide. Both forward and reverse reads were kept after trimming. Extremely low-quality bases (base quality less than 3) were removed from the ends of the reads. Thereafter, the sliding window approach was implemented in Trimmomatic to remove low-quality regions of the reads. Here, four nucleotides (window size) were assessed at a time and if the average quality in the window fell below a Phred-scaled quality threshold of 20, the bases were trimmed. This prevented the loss of high-quality data if a single low-quality base was present in the window. Finally, any sequences shorter than 36 bases were dropped. Only those sequences that retained both the forward and reverse reads (i.e. paired sequences) after trimming and filtering were mapped to the reference genome.

### Preparation of the reference genome

The African Cape buffalo (*Syncerus caffer caffer*) genome (NCBI accession number: PRJNA341313), with an estimated size of 2.732 gigabases (Gb), was used as the reference genome in this study^26^. Due to the large number of scaffolds and contigs that constituted this reference genome (442,402), the scaffolds and contigs were joined into 51 super-scaffolds, or artificial chromosomes. This was done because the HaplotypeCaller tool from the Genome Analysis Toolkit v3.8.0 (GATK)^33^, which was used for SNP genotyping (see below), cannot handle more than a few hundred scaffolds in the reference genome. The sequences were thus concatenated using ScaffoldStitcher^34^. This tool can only join one type of sequence (scaffold or contig) at a time. Thus, scaffolds were joined first, with a spacer of 1000 N’s between scaffolds, to prevent reads mapping to multiple scaffolds. A maximum length of 60 megabases (Mb) was chosen for the super-scaffolds (i.e. ScaffoldStitcher would not make a super-scaffold longer than 60 Mb). The joining of scaffolds resulted in 45 super-scaffolds (Super_Scaffold0 to Super_Scaffold44). Thereafter, contigs were joined with the same parameters, except the maximum length was adjusted to 100 Mb, to get as close as possible to the diploid number of chromosomes (2n = 52) of Cape buffalo. This resulted in six additional super-scaffolds (Super_Scaffold45 to Super_Scaffold50) and a total of 51 super-scaffolds.

### Processing of resequenced genomes

Paired sequences were aligned to the 51 super-scaffolds of the reference genome using the BWA-MEM algorithm in the Burrows-Wheeler Aligner (BWA) v0.7.12^35^, with default settings, except that split hits were marked as secondary (–M option) for downstream compatibility with Picard’s markDuplicates tool. The output files from BWA were passed directly to Samtools v1.3.1^36^ to be sorted by coordinates and output as binary alignment (BAM) files. Picard v2.6.0^37^ was used to add ReadGroups to the reads in the BAM files, as these are required by various GATK tools. Thereafter, duplicate reads were marked using the markDuplicates tool in Picard. Alignment and genome coverage statistics were collected using Picard’s AlignmentSummaryMetrics and CollectWgsMetrics tools.

The HaplotypeCaller tool from the GATK v3.8.0 was used to identify and call variants in each sample individually in the emit reference confidence mode (-ERC GVCF). In this study, the heterozygosity value used to compute prior likelihoods that a site is non-reference (-hets) was 0.03 and the minimum base quality score (-mbq) to consider a base for calling was 20. The output file, a genomic variant call format (gVCF) file, contains a record of all variant and non-variant sites for that sample.

In order to call variants across all 40 samples simultaneously (as a cohort), the GenotypeGVCFs tool from the GATK was used. This tool aggregates individual sample gVCF files produced by HaplotypeCaller and produces genotype likelihoods and re-genotypes the combined records. GenotypeGVCFs by default uses a minimum Phred-scaled confidence threshold at which variants should be called (-stand_call_conf) of 10, which allows high sensitivity of variant identification at the cost of an increased number of false positives. However, potential false positives can be reduced during variant filtering. Furthermore, loci found to be non-variant after calling were included in the output file (-allSites) to retain as much genome-wide data as possible. GenotypeGVCFs analysis was carried out separately for each super-scaffold. The VCF output files for the super-scaffolds were then combined using the CatVariants tool from the GATK to produce one VCF file containing the variant sites for all samples across all super-scaffolds.

### Variant filtering

Variants were filtered using BCFtools v1.6^38^. First, at an individual level, sites covered by fewer than two reads, more than 40 reads and a genotype quality of less than 30 were removed. Thereafter, at the variant level (across all individuals), INDELs and other variants were removed by selecting only SNPs and retaining those SNP sites that had a minor allele count of at least two, less than 50% missing data and a maximum of two alleles at the locus. Loci with an allele frequency of greater than 0.9 were removed, as well as loci with a total depth of coverage of 500 or more across all individuals. Sites with excessively high coverage are likely false variants resulting from the presence of paralogs^5^. Statistics such as the number of SNPs and the transition/transversion (Ti/Tv) ratio were calculated using the stats command in BCFtools. The resulting filtered VCF file, henceforth referred to as the GATK-VCF, containing the high-confidence SNPs, was used in the individual inbreeding estimation, the detection of inbreeding tracts and population structure analyses (see below).

### Genome-wide diversity

Genome-wide (global) nuclear heterozygosity was calculated per sample using ANGSD v0.918^39^ following the workflow provided on the ANGSD wiki (http://www.popgen.dk/angsd/index.php/Heterozygosity). ANGSD uses genotype likelihoods (GLs) of variants, which usually provides more accurate results with low coverage data, compared to using called genotypes, as the uncertainty of the data is taken into account^39,40^. ANGSD provides several models for estimating genotype likelihood, with the -GL flag. Two of these models were compared in this study to determine their effect on the estimates of genome-wide heterozygosity, namely the Samtools method (-GL 1, usually the option used in estimating heterozygosity), which accounts for sequencing errors and performs corrections and the GATK method (-GL 2), the implementation of which in ANGSD does not account for errors^41^.

To calculate heterozygosity, the folded site allele frequency likelihood was first estimated using the -doSaf command in ANGSD, with the reference genome providing the ancestral state. The same BAM files that were used for genotype calling in GATK were used as input for this analysis. Since these BAM files contained raw alignments, the data had to be filtered within ANGSD. Thus, mapping quality was adjusted for excessive mismatches using the -C 50 flag, as recommended when BWA was used for alignment. Thereafter, reads that were flagged as bad (-remove_bads), did not map to a unique location (-uniqueOnly), had a mapping quality of less than 30 (-minmapq) and did not have a mate (-only_proper_pairs) were removed. Bases with a quality score of less than 20 (-minQ) were also removed. Since the site allele frequency estimation is based on the genotype likelihood (and not called genotypes), this was calculated in ANGSD using either the -GL 1 (Samtools) or the the -GL 2 (GATK) flag. Thereafter, the site frequency spectrum (SFS)^42^ was estimated from the site allele frequency using the realSFS sub-program in ANGSD, and the SFS in turn was used to calculate the genome-wide heterozygosity per sample in R v3.6.2^43^_._ This was achieved by dividing the number of heterozygous sites (the second entry in the SFS) by the total number of sites (first entry + second entry in the SFS) to obtain the proportion of heterozygous sites for each genome.

To investigate the effect of low coverage data on the estimation of genome-wide heterozygosity, we subsampled the reads in the BAM files of four individuals (one from each population) to 25% and 50% using Samtools v1.6 and the view command with the -s flag. The same seed (1459) was used for subsampling reads at 25% and 50% for all individuals. Genome-wide heterozygosity was calculated as before, using the Samtools genotype likelihood model (-GL 1), and compared to the heterozygosity obtained with no subsampling. Mean coverage of the subsampled BAM files was calculated using the CollectWgsMetrics tool in Picard.

We compared the distributions of heterozygosity of each population using a two-sided non-parametric Wilcoxon–Mann–Whitney test for a difference in means in R. This test was used as the KNP heterozygosity data were not normally distributed as shown by a Shapiro–Wilk test for normality in R (*p*-value = 9.773e-6). Data for the other populations were normally distributed. We also compared the genome-wide heterozygosity obtained for each population and for all Cape buffalo samples combined to other wild mammalian species for which genome-wide heterozygosity has been estimated, and plotted these values in the context of census population size and IUCN Red List status for each species (as on 03/08/2020). Finally, we used the mean genome-wide heterozygosity across all samples as a proxy for theta (θ), as done by Ekblom et al.^44^ and Humble et al.^45^, in the equation θ = 4*N*_e_µ to calculate long-term *N*_e_ of the Cape buffalo subspecies assuming 1.5e−8 as the per site/per generation mutation rate^46^.

### Inbreeding

Individual inbreeding coefficients (*F*) were estimated using ngsF v1.2.0^47^, which uses the GLs in an expectation–maximization (EM) algorithm under a probabilistic framework to calculate inbreeding coefficients^47^. The *F* calculated is the proportion of sites across the genome of the individual in which the observed alleles are identical by descent^47–49^. The program requires the input GLs to be in the same binary format as used by the program BEAGLE. The filtered GATK-VCF file contained both the high confidence genotype calls of all the samples and the GLs. This file could thus be used as input in ANGSD, which is able to convert the file into the required binary BEAGLE format. This was achieved by using the -vcf-gl flag in ANGSD, to indicate the input file was a VCF file containing GLs, and the -doGlf 3 flag, instructing ANGSD to output the GLs in binary BEAGLE format. It is also important to instruct ANGSD not to attempt to calculate GLs from this input file (the default behaviour), by using the -GL 0 flag.

Individual inbreeding coefficients were estimated by first obtaining approximate inbreeding coefficients using the faster -approx_EM method, with a maximum root mean squared difference between iterations to assume convergence of the algorithm of 1.0e−5 (-min_epsilon) and random initial values for individual inbreeding and site frequency (-init_values). The output of this initial estimation was then used as the initial values for the full implementation of the algorithm (without the -approx_EM flag), where a -min_epsilon of 1.0e−7 was used to assume convergence. To avoid convergence to local maxima, this two-step analysis was repeated ten times by using the bash script provided with the program for this purpose.

Inbreeding, or identical by descent (IBD), tracts within the genome of each individual were identified using ngsF-HMM^40^, packaged with ngsTools v1.0.1^50^. Once again, GLs are utilised in this program, which uses a two-state Hidden Markov Model (HMM) to identify inbreeding tracts. This analysis required the input file containing GLs to be in the non-binary BEAGLE format and was prepared from the GATK-VCF file as for the binary BEAGLE format, except that the -doGlf 2 flag was used in ANGSD. Inbreeding tracts were then identified using an initial frequency value (-freq) of 0.1 and initial inbreeding and transition values (-indF) of 0.1 and 0.1, respectively. A -min_epsilon of 1.0e−7 between iterations was used to assume convergence of the algorithm. Inbreeding tracts were then plotted in R using the R script provided with ngsF-HMM.

### Population structure

Principal component analyses (PCA) were carried out using the ngsCovar tool in ngsTools v1.0.1^50,51^. First, the required genotype probability input file was prepared in ANGSD from the GATK-VCF file using the -doGeno 32 and -doPost 1 flags. The former instructs ANGSD to write the posterior probabilities of the three genotypes as binary and the latter informs ANGSD to use the per-site allele frequency as a prior in calculating the posterior probability of the genotypes. The covariance matrix between individuals was then estimated in ngsCovar based on these genotype probabilities, not calling genotypes (-call 0) and not normalising the data using allele frequencies (-norm 0), as this could give more weight to low frequency variants which are more difficult to estimate. The resulting covariance matrix was converted into eigenvectors and plotted in R using the plotPCA R script provided with the ngsTools package.

Individual admixture proportions were estimated with NGSadmix v32^52^. This program requires the same GL input file as ngsF-HMM and implements an EM algorithm using the GLs. NGSadmix was implemented for values of *K* ranging from one to six, with a log likelihood difference for 50 iterations of less than 0.01 required to assume convergence of the algorithm (-tolLike50). In order to identify the most likely number of subpopulations, the likelihood of each value of *K* was plotted and the value at which the curve started to plateau was interpreted as representing the most likely number of subpopulations. The individual assignment results were plotted in R using the plotAdmix R script provided with the ngsTools package.

### Demographic history

The demographic history of each population was inferred by applying the pairwise sequentially Markovian coalescent (PSMC) model to autosomal sequences of each individual genome^53^. Bovid X chromosome sequences were identified in the buffalo reference genome using a synteny analysis to the cattle (*Bos taurus*) X chromosome implemented in Satsuma Synteny v3.1.0^54^. The cattle X chromosome sequence (accession number: AC_000187.1) was obtained from the cattle genome build UMD3.1.1 on the National Center for Biotechnology Information (NCBI) database.

Due to the concatenation of scaffolds and contigs of the reference genome into super-scaffolds, bovid X chromosome sequences were present on all super-scaffolds except Super_Scaffold50. Thus, scaffolds could not simply be removed for PSMC analysis. Instead, the output from Satsuma Synteny that provided a list of the positions of syntenic X chromosome regions in the buffalo genome in 1-based format was manually converted to a three column (chromosome, start, end) BED format (0-based, half-open). BEDTools v2.26.0^55^ was then used to create the complement BED file, i.e. regions of non-X chromosome sequences, to be used in the next step—consensus sequence preparation.

The input file for PSMC was prepared by creating a consensus diploid sequence for each sample using the mpileup command in Samtools v1.3.1, with the BAM file as input and the artificially concatenated buffalo genome as reference, while adjusting for mapping quality (-C 50). The X chromosome sequences were excluded by instructing Samtools to only perform the pileup in the regions specified by the non-X chromosome BED file (using the -l flag). The output from Samtools mpileup was passed to BCFtools v1.3.1 to construct the actual consensus sequence using the call command and the -c flag. This consensus VCF file was then converted to fastq format using the vcf2fq command of the vcfutils script (provided with Samtools), while filtering out bases with quality lower than five (-Q), a minimum read depth of two (-d) and a maximum read depth of 40 (-D). Finally, the consensus sequence in fastq format was converted to the input format required by PSMC using the fq2psmcfa script provided with PSMC, while filtering out bases with a quality score lower than 20.

PSMC analysis was carried out on all 40 buffalo samples using the default parameters, with the exception of the -r flag that was changed to five and the -p flag that was changed to “4 + 25 × 2 + 4 + 6”, as these parameters had been shown previously to be meaningful in humans and other species^46,53,56,57^. PSMC divides the diploid consensus sequence into 100 bp non-overlapping bins, scores the bin as heterozygous if a heterozygous site is present, or otherwise as homozygous^53^ and subsequently infers time to the most recent common ancestor of alleles in heterozygous sites. One hundred bootstrap replicates were performed for each sample by splitting the input file into multiple regions and performing bootstrapping over these regions. The inverse distribution of coalescence events is then usually interpreted as the effective population size (*N*_e_) over time. However, what is in fact inferred is the inverse instantaneous coalescent rate (IICR)^58^, which nonetheless can be interpreted as *N*_e_ in a panmictic population (but not in a structured population)^58,59^. Thus, the IICR as inferred by PSMC was plotted over time with the x-axis scaled using a per generation mutation rate for buffalo of 1.5e−8 and a generation time of 7.5 years (the estimated per site per year mutation rate of 2.0e−9 by Chen et al.^46^ converts to 1.5e−8 for a generation time of 7.5 years). PSMC plots were constructed in R v3.6.2^43^, using ggplot2 v3.3.0^60^, by editing the script of Emily Humble (available at: https://github.com/elhumble/SHO_analysis_2020), which uses the plotPsmc R function from Liu and Hansen^61^. A link to all code used in this study is provided in the data availability section.

## Results

### Genome resequencing, assembly and variant filtering

We generated 40 whole-genome sequences of buffalo from four national parks in South Africa (Fig. 1), with a targeted depth of 10× (Supplementary Table 1). After quality filtering of reads, mapping to the reference genome and removing duplicates, a mean depth of 7.14× coverage was realised (Supplementary Tables 2 and 3), with similar depth of coverage between the four localities (Supplementary Table 4). The high-quality mapped reads covered ~ 2.6 Gb of the genome of ~ 2.7 Gb (Supplementary Table 3). The GATK pipeline identified approximately 49 million raw single nucleotide polymorphism (SNP) sites and 6 million INDELs across all samples and all super-scaffolds. After filtering, this was reduced to approximately 3.8 million high quality SNPs (Supplementary Table 5). The Ti/Tv ratio is an indication of the quality and specificity of SNP calls, and is expected to be between ~ 2.0 and 2.1 for mammals^62^. A Ti/Tv ratio that is substantially lower than 2.0 could be an indication of low-quality sequencing data^3,62^. The Ti/Tv ratio of the raw SNP data set was 1.90 and increased to 2.02 in the filtered data set (the GATK-VCF file) (Supplementary Table 5).

### Genomic diversity and inbreeding

The comparison of the two genotype likelihood models in ANGSD, namely Samtools (-GL 1) and GATK (-GL 2), showed substantially higher heterozygosity estimates were obtained for all samples with the GATK method (Supplementary Fig. 1), which does not account for error in its implementation in ANGSD. Given that the Samtools method does account for error, this model was used in all subsequently reported heterozygosity estimates, as well as in the estimation of long-term *N*_e_. The comparison of heterozygosity between subsampled BAM files, to determine the effect of low coverage data on this measure, showed the lowest heterozygosity for the low coverage data (25% of reads subsampled), higher heterozygosity for the medium coverage data (50% of reads subsampled) and the highest value was obtained when no reads were subsampled (Supplementary Fig. 2).

**Figure 2:**
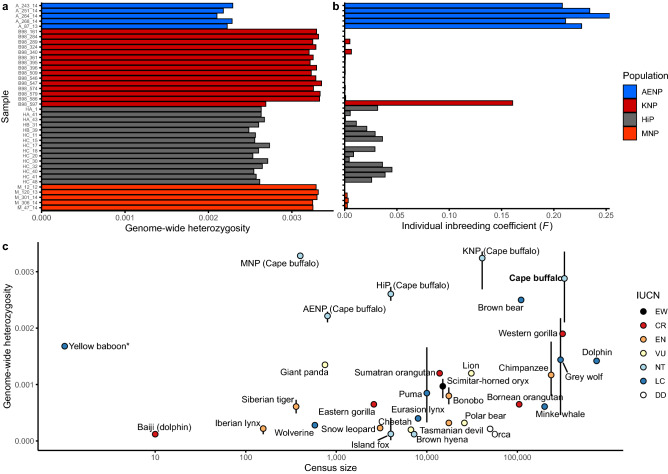
Genome-wide diversity indices. (**a**) Individual genome-wide heterozygosity estimates for each sample (-GL 1 Samtools). (**b**) Individual inbreeding estimates (*F*) for each sample. (**c**) Genome-wide heterozygosity of mammalian species plotted against census size. Colours indicate IUCN Red List categories as on 03/08/2020. The vertical bars indicate the range of heterozygosity across multiple individuals, where available. The mean and range across all Cape buffalo samples in this study is shown (bold face), as well as the mean and range for each population. The range for MNP is plotted, but is too narrow to extend beyond the plotting point. *No estimate of census size is available for the yellow baboon; thus, it was plotted at a value of 1 to still be included in the figure. The figure was adapted from Ekblom et al.^44^ Data for a, b and c are provided in Supplementary Tables 6 and 7.

Individual genome-wide heterozygosity estimates (Fig. 2a) were significantly higher in the Kruger National Park (KNP) and Mokala National Park (MNP) compared to Hluhluwe-iMfolozi Park (HiP, *p*-values = 5.16e−8 and 1.29e−4) and Addo Elephant National Park (AENP, *p*-values = 1.29e−4 and 7.94e−3). Heterozygosity was also significantly higher in HiP compared to AENP (*p*-value = 1.29e−4), while that observed in MNP was statistically equivalent to the heterozygosity of KNP (*p*-value = 0.866). One individual from KNP, B98_597, had substantially lower genome-wide heterozygosity than other individuals from KNP (Fig. 2a, Supplementary Table 6). Cape buffalo had significantly higher genome-wide heterozygosity compared to all species included in Fig. 2c, except the brown bear and the grey wolf, for which heterozygosity estimates overlapped with those of Cape buffalo (Fig. 2c, Supplementary Table 7). The populations KNP and MNP had higher heterozygosity than all other species included in Fig. 2c.

Buffalo from AENP had high individual inbreeding coefficients (*F*) (0.21–0.25, Fig. 2b, Supplementary Table 6). Individuals from HiP had inbreeding coefficients close to zero, despite having relatively lower levels of genome-wide heterozygosity compared to KNP and MNP. Individuals from KNP and MNP also had inbreeding coefficients close to zero, except for sample B98_597 for which *F* = 0.16.

Inbreeding (or IBD) tracts were evident across most of the super-scaffolds in the samples from AENP, highlighting the inbred nature of these buffalo (Supplementary Fig. 2). Such tracts were markedly absent in buffalo from the other populations, except for the slightly inbred sample from KNP, B98_597 (Supplementary Fig. 3). The long-term *N*_e_ of Cape buffalo was estimated to be 48,007 (range 35,038–55,938).

### Population structure

**Figure 3:**
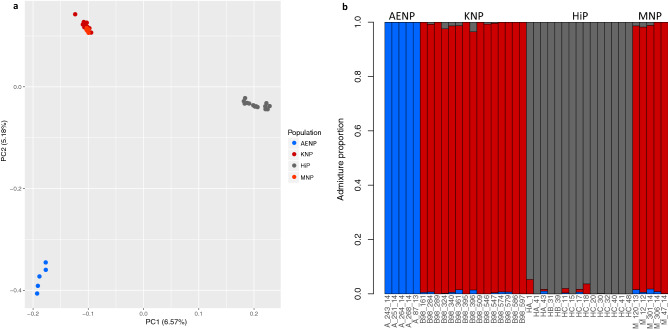
Population structure of buffalo included in this study. (**a**) Principal component analysis (PCA) plot. The percentage of variation explained by each principal component (PC) is shown in parenthesis on the x- and y-axis. (**b**) Individual assignment plot of the NGSadmix analysis at *K* = 3. Plots for values of *K* = 2, 4, 5 and 6 are shown in Supplementary Fig. 5.

The principal component analysis (PCA) separated the samples from the four populations into three distinct clusters (Fig. 3a). Samples from KNP and MNP clustered together, while those from AENP and HiP formed separate clusters. The NGSadmix analysis of admixture and population structure reached convergence for all values of *K* investigated (one to six). The most likely number of subpopulations was deemed to be *K* = 3, interpreted as the point where the likelihood curve started to plateau (Supplementary Fig. 4). As in the PCA, samples from AENP and HiP were separated into separate clusters and those from KNP and MNP formed a single cluster (Fig. 3b). Individual assignment plots at *K* = 2, 4, 5 and 6 are shown in Supplementary Fig. 5.

### Demographic history

**Figure 4:**
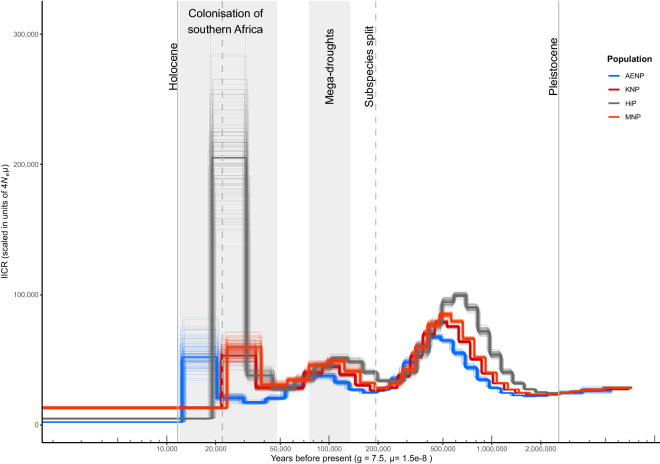
Pairwise sequentially Markovian coalescent (PSMC) inference of the inverse instantaneous coalescent rate (IICR) in Cape buffalo. One individual from each sampling locality is shown (AENP- A_243_14, KNP- B98_509, HiP- HC-32 and MNP- M_120_13). The x-axis represents time in years, calibrated using a generation time (g) of 7.5 years and a per site per generation mutation rate (µ) of 1.5e−8. The y-axis shows IICR, which can be interpreted as *N*_e_ in an unstructured population. The faded lines show the 100 bootstrap replicates for each sample. The unlabelled vertical dashed line at 22,000 years ago indicates the Last Glacial Maximum (LGM).

The demographic history, as represented by the inverse instantaneous coalescent rate (IICR), of each population was investigated by PSMC analysis of each individual buffalo genome (Supplementary Fig. 6). The demographic history of each population, represented by one sample each, is shown in Fig. 4. The same general trend of three expansion-decline events between ~ 1 million and ~ 20 thousand (ka) years ago was inferred for each population and all samples (Fig. 4, Supplementary Fig. 5). Assuming a non-structured population in the Pleistocene, we can interpret the IICR as equivalent to the effective population size (*N*_e_) of buffalo through this period. After the first increase approximately 500 ka, a maximum *N*_e_ of between 64,000 and 100,000 was reached. The decline after this resulted in a minimum *N*_e_ of approximately 30,000 around 200 ka. The next expansion event, peaking at ~ 100 ka, resulted in a maximum *N*_e_ of just under half the previous maximum, which was followed by a decline at ~ 50 ka that reached about the same minimum *N*_e_ as the previous decline. The third expansion-decline event occurred between 35 and 20 ka, although IICR estimates become less reliable in this final window, as shown by the greater variance between bootstrap replicates. The timing of these events was similar in all samples, until approximately 50 ka, at which point the IICR curve of AENP started to diverge from those of KNP, MNP and HiP, with the changes in IICR becoming comparatively delayed in AENP.

## Discussion

Genomic resources are becoming increasingly accessible for non-model species as a result of the decreased costs of next-generation sequencing technologies^1^. Here, we take advantage of this advancement and the published African buffalo (*Syncerus caffer*) genome^26^ to perform the first population genomics study of the Cape buffalo (*S. c. caffer*). This subspecies is widespread from East to southern Africa (Supplementary Fig. 7) and is an ecologically- and economically important bovid species. In this study we produced 40 low coverage Cape buffalo genomes from four protected areas in South Africa, representing three distinct gene pools. We use these data to characterise genome-wide diversity, the effects of recent population bottlenecks on genomic diversity and the demographic history of the species. The results provide new insights regarding the genetic diversity of the populations, as well as the historical demography of the species, which have implications for its conservation. The genomic data generated in this study provide a valuable resource for future studies of the species, which may aid in its conservation, the management of economically important bovid diseases and sustainable management in both natural and privately-owned populations of the species.

Our first aim was to determine the effect on genome-wide diversity of the recent bottleneck experienced by Cape buffalo in southern Africa from the 1890s to the mid-1900s, caused by disease outbreaks and hunting. We found that Cape buffalo had higher genome-wide heterozygosity (mean = 0.0029, range = 0.0021–0.0034) than any of the 27 wild mammalian species for which similar analyses have been conducted. The range of heterozygosity values for buffalo overlapped only with that of the brown bear (*Ursus arctos*, 0.0026)^63^ and the grey wolf (*Canis lupus*, mean = 0.0014, range = 0.0005–0.0022)^64^. The high genome-wide diversity of Cape buffalo may seem surprising given the estimated 95% reduction in population size experienced in southern Africa ~ 100 years ago. Comparisons of genome-wide heterozygosity between studies of different species, while informative to a certain extent, should be interpreted with caution, as heterozygosity estimates are sensitive to the applied bioinformatics pipeline and the variant filtering applied^44^, as shown in this study (Samtools vs GATK method in ANGSD) and elsewhere^65^.

Nonetheless, the high genome-wide diversity may be explained by several factors. First, it should be noted that most of the species for which this type of analysis has been done are listed as Vulnerable or at a higher threat status on the IUCN Red List, suggesting significant and sustained population declines or small population sizes in these species, which generally result in low genetic diversity^66^. Furthermore, genomic studies of species listed as Near Threatened or Least Concern may focus on locally threatened populations of conservation concern, potentially harbouring low genetic diversity. African buffalo has recently been up-listed from Least Concern to Near Threatened, indicating that high genetic diversity should be expected. In essence, the current database of mammalian genome-wide heterozygosity estimates is likely biased towards species and populations with low genetic diversity. We expect that as more mammalian species, and more populations, are studied at a genomic level there will be more instances of high genome-wide heterozygosity.

Second, while the percentage reduction in the southern African buffalo population may have been extreme, the loss of genetic diversity is dependent on the absolute number of individuals that remain after the initial decline, the duration of the bottleneck in generations and the growth rate of the population after the bottleneck^67^. In other words, tens of individuals must remain for many generations before significant loss of genetic diversity occurs, while a high growth rate after the bottleneck will result in more new mutations, creating genetic diversity, compared to a slow growth rate. The buffalo population in KNP likely did not have a population size less than 1600 individuals after the disease- and hunting induced bottleneck of the 1890s and early 1900s^16^. Assuming the bottleneck effectively ended when KNP was proclaimed as a protected area in 1898, the bottleneck lasted fewer than two buffalo generations. Furthermore, there was lower hunting pressure in KNP, due to the presence of malaria^16,68,69^. The KNP population has since recovered well to ~ 40,000 buffalo today^10^. Thus, the bottleneck in KNP was apparently not severe enough, nor lasted long enough, to result in any significant loss of genetic diversity. Lastly, KNP is connected to neighbouring protected areas in Zimbabwe and Mozambique, thus allowing gene flow to maintain, or increase, genetic diversity in this population.

Interestingly, one KNP individual (B98_597) had substantially lower genome-wide diversity than its KNP counterparts and a comparatively high individual inbreeding coefficient (*F*, the proportion of sites across the genome in which the observed alleles are identical by descent) of 0.16. This individual is most likely the offspring of two related individuals (probably of second-order relatedness, such as half siblings), which is an event that is expected to occur occasionally in buffalo herds, due to their hierarchical mating structures, and is thus a natural consequence of their biology^7,70^.

The relatively high long-term *N*_e_ of Cape buffalo estimated in this study (48,007) suggests a large population and high genetic diversity in the past. Assuming the *N*_e_ of Cape buffalo is between 10 and 30% of the census size, *N*_c_^16,22^_,_ the historical population size was between 160,023 and 480,070 individuals. This overlaps with the upper limits of long-term *N*_c_ estimated by Smitz et al.^15^ for the northern cluster in southern Africa in their microsatellite study (23,000–250,000) and is higher than their estimate for the central cluster which included KNP (10,000 to 100,000). Our estimate overlaps with the current estimated *N*_c_ of the subspecies across its entire range of approximately 473,000^5^, as well as the estimated *N*_c_ in southern Africa (Botswana, Mozambique, Namibia, South Africa and Zimbabwe) of ~ 209,690^10^, suggesting a stable long-term population size of the subspecies, notwithstanding the recent declines that contributed to the up-listing of African buffalo from Least Concern to Near Threatened on the IUCN Red List^5^.

The heterozygosity in MNP was statistically equivalent to that observed in KNP, its source population, indicating that the founder effect^71^ was likely avoided in the establishment of this disease-free population of KNP buffalo (aim ii of this study). This implies that the disease-free breeding programme implemented by SANParks to establish the MNP population had a sufficiently high number of breeders (i.e. founders) that adequately represented the genetic diversity present in KNP^67^. However, it may be that too few generations have passed between the final introduction of buffalo to MNP in 2007 and the time samples were collected (2012–2014) for a significant loss of genetic diversity to occur. Thus, we caution that the genetic diversity in MNP should be closely monitored and supplemented with KNP buffalo if necessary, given its recent establishment and relatively small population size (~ 400 buffalo), which may result in genetic drift leading to a loss of genetic diversity^72^.

The heterozygosity estimates of HiP buffalo were intermediate between KNP-MNP and AENP, while the individual inbreeding levels were close to zero and no inbreeding tracts were detected. These results are in line with those of O'Ryan et al.^16^ based on microsatellite data, indicating that the bottleneck was slightly more severe in HiP compared to KNP, but not as severe as in AENP. The lowest population size recorded for HiP was 75 individuals in 1929, but the population appears to have recovered well since then with > 8000 buffalo recorded in 1998 and > 4000 at present. Additionally, hunting pressures were low in HiP, due to lower human activity in the area as a result of the high prevalence of the tsetse fly, the vector for *Trypanosoma* species, which causes sleeping sickness in humans^73^. HiP may have recovered to higher levels of genetic diversity had there been some connection to other populations, but this was unlikely, due to the fragmented nature of South African protected areas. Given that natural connectivity between HiP and other buffalo populations is unlikely to be re-established due to human expansion, the genetic diversity in HiP should be monitored and, if required, augmented with buffalo from an appropriate source, such as MNP. It should be noted that for the interpopulation comparisons of heterozygosity, sample sizes were small and thus the outcome of the statistical tests should be interpreted with caution.

The high heterozygosity estimates, compared to other mammals, and high *F* estimates obtained for AENP may seem a somewhat paradoxical result. However, these results correlate within the context of the species and this data set, as AENP had the lowest heterozygosity of the four protected areas sampled. Microsatellite analysis of a larger sample size from AENP (*N* = 79) showed low allelic richness, but also a relatively low population-level inbreeding coefficient (*F*_IS_) of 0.049 that was not significantly greater than zero^17^. Here it should be noted that *F*_IS_ is a measure of non-random mating and thus does not represent the extent of individual inbreeding, *F*, in a population^48^. This is evident from the five unrelated AENP samples in this study, which had *F* estimates between 0.21 and 0.25, substantially higher than the population-level inbreeding estimate. Other mammalian species for which the ngsF software has been used to estimate *F* include the muskox (*Ovibos moschatus*, 0.00 ≤ *F* ≤ 0.10)^74^, the grey wolf (*C. lupus*, 0.00 ≤ *F* ≤ 0.71)^75^, Przewalski’s horse (*Equus ferus* ssp.* przewalskii*, 0.00 ≤ *F* ≤ 0.32)^76^ and the house mouse (*Mus musculus*, ~ 0.40 < *F* <  ~ 0.85)^77^. The high level of inbreeding in AENP was also exemplified by the large number of inbreeding tracts identified in the AENP genomes, present on most super-scaffolds in every individual. These results indicated that close or, more likely, pervasive inbreeding (as a result of a small population size and genetic drift^78^) is prevalent and problematic in the AENP buffalo population.

The offspring from the mating of first-order relatives (e.g. full siblings) would have an individual inbreeding coefficient of 0.25 calculated from a pedigree (*F*_p_), while the offspring of the mating of second-order relatives (e.g. half siblings) would result in an *F*_p_ of 0.125^49^. However, the realised *F* (as calculated in this study) would vary between offspring for each of these parental categories, due to different pedigrees and the randomness of Mendelian inheritance^49^. Thus, we may conclude that the five different sets of parents of the five samples from AENP included in this study (which were unrelated based on microsatellite genotypes^17^), were effectively at the level of half- or full siblings. Vieira et al.^47^ showed that their method for estimating *F* (implemented in this study) was robust for small sample sizes (*N* = 10). While these results should be interpreted with caution at the present time, given the small sample size from AENP (*N* = 5), the level of inbreeding in AENP buffalo is nevertheless a cause for concern.

These high inbreeding coefficients may be explained by a longer lasting and more severe bottleneck, as compared to KNP and HiP. Hunting pressure likely started earlier in AENP, as the Cape Dutch Colony expanded in the late 1700s, and continued longer, since AENP was only proclaimed a protected area in 1931, as opposed to 1898 and 1895 for KNP and HiP, respectively. Thus, the bottleneck in AENP was at least 34–37 years (approximately four to five buffalo generations) and up to ~ 160 years (~ 21 buffalo generations) longer compared to KNP and HiP. Additionally, the hunting pressure was likely more severe on the AENP population, given the absence of the tsetse fly and malaria in this region. The lowest population size recorded in AENP after the bottleneck was 52 buffalo in 1985 and the present population is about 800 individuals. Thus, this population did not seem to recover as quickly and to the same level as HiP and KNP, perhaps due to a reduced carrying capacity of the environment, with the Cape Floristic Region consisting predominantly of nutrient-poor soil and unpalatable plants^79,80^. Finally, being on the edge of the entire distribution of the species and isolated from all other relict populations, the AENP population could not maintain, nor increase, genetic diversity through gene flow with other populations.

Microsatellite studies have previously indeed found low genetic diversity in the AENP population and proposed genetic augmentation via KNP, or more appropriately, disease-free MNP buffalo^16,17^. However, the results of genomic analyses in this study, showing the high inbreeding levels, and inbreeding tracts distributed throughout the genomes of AENP buffalo, highlight the urgency with which genetic supplementation should be implemented to avoid the manifestations of inbreeding depression and increase resilience of the population. O'Ryan et al.^16^ previously estimated that one breeding bull per generation (7.5 years) from KNP would increase the genetic diversity of the AENP population and restore it to ~ 90% of the diversity in KNP, while also preventing genetic swamping of AENP to prevent the loss of unique genetic variation and/or local adaptation. Supplementation of AENP, a disease-free population, with KNP buffalo was precluded by the presence of bovine tuberculosis in KNP at the time of the O'Ryan et al.^16^ study in 1998. However, disease-free KNP buffalo are now available in MNP, thus making genetic supplementation of AENP possible.

For our third aim, the population structure analyses (PCA and NGSadmix) with genome-wide data indicated that the populations were genetically differentiated, which agrees with microsatellite studies for AENP, HiP and KNP^15–17^. KNP and MNP, which have not been analysed together before, formed a single genetic cluster. This was expected since the MNP population was recently established using KNP buffalo as part of the South African National Parks (SANParks) disease-free buffalo breeding programme between 1999 and 2007^24^ (*Pers. Comm.* D. Zimmerman 2015). Henceforth these two populations will be referred to as the KNP-MNP population. Past population differentiation of southern African buffalo is thought to have been relatively weak, even across large geographic distances^13,16,81^, owing to the widespread occurrence, and strong dispersal ability, of buffalo^7,14^. Therefore, the genetic structuring of buffalo populations in southern Africa is likely due to disease outbreaks and anthropogenic factors resulting in habitat fragmentation, population decline and limited or no gene flow in the last ~ 150 to ~ 300 years^15,16^. This subsequently resulted in drift driving population differentiation, particularly in smaller populations such as AENP and HiP^15,16^. However, there is strong evidence for a rapid decline in Cape buffalo starting ~ 5000 years ago, coinciding with the onset of drier conditions during the Holocene and the expansion of pastoralism, with humans and domesticated cattle expanding to displace buffalo from much of its range^27,82^. This may suggest earlier fragmentation of buffalo populations than 150–300 years ago. Indeed, Smitz et al.^15^ found support for a split occurring approximately 6000–8400 years ago between populations in the north of Zimbabwe and Botswana (northern cluster) and those in the south of Zimbabwe and Mozambique and north of South Africa (central cluster). While neither HiP nor AENP were included in those analyses, the results suggest that these two populations may have been isolated earlier than 150–300 years ago, with recent population bottlenecks further exacerbating genetic differentiation of these populations.

Reconstructing the Pleistocene demographic history of African buffalo (the final aim of this study), using the pairwise sequentially Markovian coalescent (PSMC) model, revealed fluctuating inverse instantaneous coalescent rates (IICR) over time, which we interpreted as change in effective population sizes (*N*_e_). While low coverage genomes can result in a loss of power to detect changes in *N*_e_^83^, this did not appear to be the case in our study. The *N*_e_ changes we detected were comparable to those found using a high coverage African buffalo genome from Kenya (thus assumed to be a Cape buffalo, *S*. *c*. *caffer*)^46^.

We analysed genomes from each population separately, to determine whether any southern African population diverged in their demographic history from the other populations. The PSMC results showed near-identical *N*_e_ trajectories for all three populations (KNP-MNP, HiP and AENP) until ~ 50 ka, at which point the trajectory of AENP became comparatively delayed. This delayed signal is likely an artefact of the low diversity in the AENP genomes, as there is currently no evidence that this is a real biological signal. Therefore, the PSMC curve presented here represents the demographic history of the ancestral population and provides support for the hypothesis of a recent (i.e. Holocene) split of the sampled populations.

A study of Lake Malawi sediments identified a period of extreme climate variability in East Africa, with high-amplitude cycles of calcareous and non-calcareous sediments, representing relatively arid and moist conditions, which ended ~ 900 ka^84^. Following this period, cycles of relative arid and moist conditions were less extreme, suggesting a more stable environment^84^. The first expansion event inferred (of the ancestral African buffalo (*Syncerus caffer*) population) from the PSMC analysis started at around this point of more stable environmental conditions, approximately one million years ago and continued to ~ 500 ka. Calcium concentrations in sediments were at one of the lowest points around 800 ka, indicating relatively moist conditions, which was mirrored in a shift from open grasslands to wooded grasslands around the same time^84^. The change in conditions would theoretically support the expansion of the species. Buffalo are highly dependent on stable water supply, usually drinking twice daily^7^ and, while buffalo are grazers, they still require some tree cover to provide shade for temperature regulation during the hottest part of the day^7^. Similar expansion-decline events during this period were observed for giraffe (*Giraffa camelopardalis*), blue wildebeest (*Connochaetes taurinus*), steenbok (*Raphicerus campestris*) and suni (*Neotragus moschatus*)^46^ and the fossil record shows high faunal turnover in East Africa approximately one million years ago, likely related the change from a grassland to a wooded grassland, or more savannah-type ecosystem^85,86^. The entire expansion-decline event between one million years ago and 250 ka may also be a signal of multiple expansion-decline events in quick succession in response to arid and moist cycles, as PSMC may struggle to detect such sudden changes, with the result being a signal of one change spread over a longer period^53^.

The second expansion event detected around 200 ka supports previous findings of an expansion in the Cape buffalo subspecies after the split between the south-eastern clade (*S. c. caffer*) and the west-central clade (the forest-type subspecies, *S. c. nanus*, *S. c. brachyceros* and *S. c. aequinoctialis*)^12,14^. The divergence of these two lineages is estimated to have occurred around 193 ka (95% CI 145–449 ka)^14^ or 130–180 ka^12^. The following decline from ~ 100 ka may be a consequence of a series of “mega-droughts” that occurred in East Africa between 135 and 75 ka^87,88^, which is also supported by the calcium sediment data from Lake Malawi^84^. This decline around 100 ka was also concurrent with declines seen in many other ruminant species, which were concurrent with increasing human effective population size^46^.

The colonisation of southern Africa (from East Africa) by Cape buffalo has been dated to approximately 48–80 ka, when environmental conditions became more favourable in the Late Pleistocene, with the transition to relatively moist savannah-like environments in southern Africa^12,14,27,89^. This hypothesis is supported by our PSMC analysis, which shows signals of expansion during this period. The final decline should be interpreted with caution, as the ability of PSMC to detect recent events diminishes after ~ 20 ka^53^ and there is support from mitogenomes for continued expansion until ~ 5 ka^27^. The approximate expansion dates into southern Africa is supported by most current fossil evidence, with some of the earliest *S*. *caffer* fossils in South Africa dated to between 43.4 ± 3.0 ka and 106.8 ± 12.6 ka, at the Klasies River Mouth site on the southern coast of South Africa^90^. At least two other sites in South Africa also support these dates, with *S*. *caffer* fossils dated to between 50.7 ± 4.7 ka and 79.7 ± 15.6 ka at Die Kelders Cave^91^ and between 57.6 ± 2.1 ka and 59.6 ± 2.3 ka at Sibudu Cave^92^. Other sites in South Africa, such as Nelson Bay Cave and Boomplaas Cave had more recent *S*. *caffer* fossils, ranging between approximately 12 ka and 21 ka^93,94^. Furthermore, it is postulated that this expansion was facilitated by the decline of the ancient long-horned African buffalo, *Pelorovis antiquus*, that inhabited this region before going extinct approximately 12 ka^12,14,95^. The PSMC results may indicate a sensitivity of Cape buffalo to climate change, raising concerns about the persistence of the species in the southern African region, as the climate becomes hotter and drier as the century progresses^96,97^.

The 40 buffalo genomes generated in this study, in combination with the Cape buffalo reference genomes^26,46^, mitogenomes^27^ and SNPs identified in previous studies^2,3,19^, indicate that African buffalo genetics may be coming of age in the genomics era. A central database of African buffalo genomics resources could propel discoveries in this species to aid in its conservation. First and foremost, should be the improvement of the published reference genome assemblies, one from KNP^26^ and the other from Kenya^46^. This would simplify and improve genomic analyses and could lead to advances in multiple lines of inquiry, e.g. delineating subspecies more accurately, with associated conservation implications. Reconstructing the recent (< 300 years) demographic history of buffalo populations across Africa to determine the effect of past climatic events and human activity on the species could further inform current conservation action and policies. A database such as this could be instrumental in constructing a linkage map, which could be developed in conjunction with, and applied in, the wildlife ranching industry to potentially identify genes underlying traits under artificial selection, as well as possible hitchhiking deleterious variants or genes^2^. Furthermore, the sampling localities in this study could provide a good test case to study the disease-response of buffalo populations at the genome level, as KNP and HiP harbour diseases such as bovine tuberculosis that are not present in AENP, provided more samples are genotyped from each population. MNP may also be studied to track the dynamics of disease-related alleles no longer under selection pressure.

## Supplementary Information

Supplementary Information.

## Data Availability

The raw reads of the genomes generated in this study have been submitted to the Sequence Read Archive (SRA) of the NCBI and are available under the BioProject accession number PRJNA645266. Code used in the analysis of this data set and production of figures for this manuscript is available at https://github.com/DeondeJager/Buffalo_PopGenomics.
