# Supplementary material for: High diversity, inbreeding and a dynamic Pleistocene demographic history revealed by African buffalo genomes
Source: Sci Rep. 2021 Feb 25;11:4540. doi: 10.1038/s41598-021-83823-8 (PMC7907399; doi:10.1038/s41598-021-83823-8)
Supplement: Supplementary file 1 — Supplementary Information. [file 41598_2021_83823_MOESM1_ESM.pdf]

**Supplementary Information for the manuscript: High diversity, inbreeding and a dynamic Pleistocene demographic history revealed by African buffalo genomes**

Deon de Jager[1]*, Brigitte Glanzmann[2], Marlo Möller[2], Eileen Hoal[2], Paul van Helden[2], Cindy Harper[3], Paulette Bloomer[1]

[1]Molecular Ecology and Evolution Programme, Department of Biochemistry, Genetics and Microbiology, Faculty of Natural and Agricultural Sciences, University of Pretoria, Pretoria, South Africa

[2]DSI-NRF Centre of Excellence for Biomedical Tuberculosis Research; South African Medical Research Council Centre for Tuberculosis Research; Division of Molecular Biology and Human Genetics, Faculty of Medicine and Health Sciences, Stellenbosch University, Cape Town.

[3]Veterinary Genetics Laboratory, Faculty of Veterinary Science, University of Pretoria, Pretoria, South Africa

*Corresponding author

Email: dejager4@gmail.com

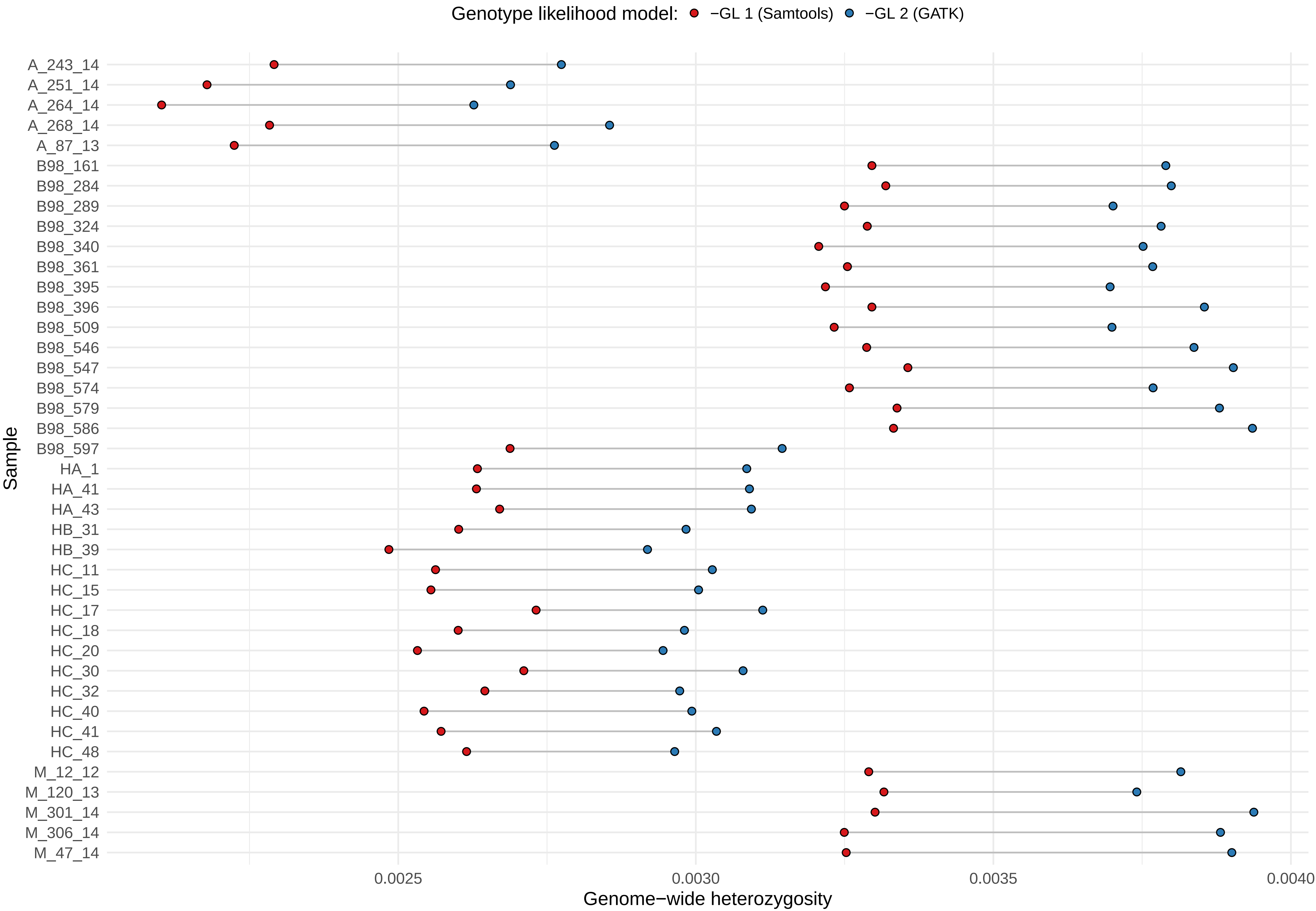

Genotype likelihood model:
−GL 1 (Samtools)
−GL 2 (GATK)
A_243_14
A_251_14
A_264_14
A_268_14
A_87_13
B98_161
B98_284
B98_289
B98_324
B98_340
B98_361
B98_395
B98_396
B98_509
B98_546
B98_547
B98_574
B98_579
B98_586
B98_597
HA_1
HA_41
HA_43
HB_31
HB_39
HC_11
HC_15
HC_17
HC_18
HC_20
HC_30
HC_32
HC_40
HC_41
HC_48
M_12_12
M_120_13
M_301_14
M_306_14
M_47_14
Sample
0.0025
0.0030
0.0035
0.0040
Genome−wide heterozygosity

**Supplementary Fig. 1. Comparison of genome-wide heterozygosity estimates using different genotype likelihood models in ANGSD.**

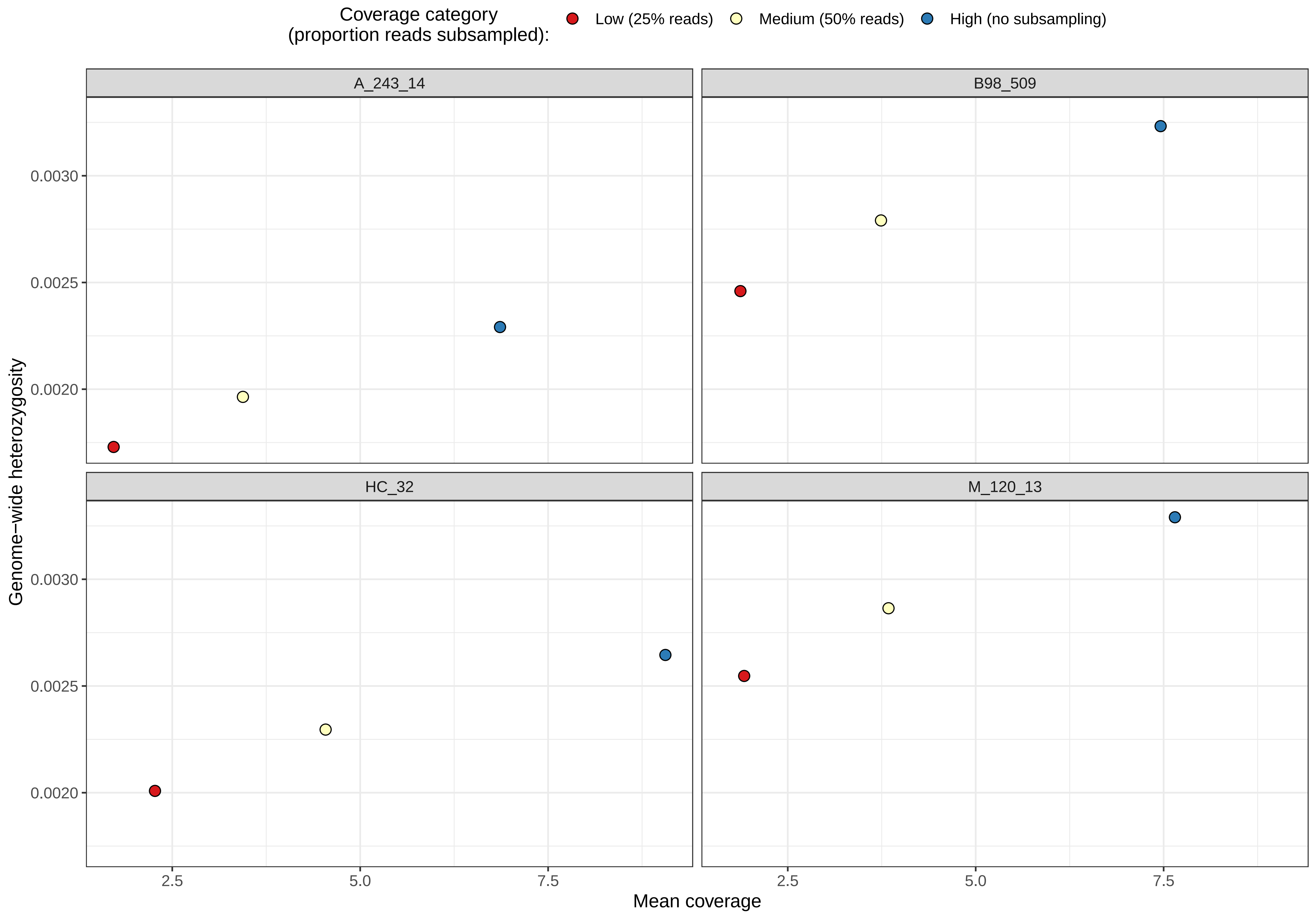
Coverage category
(proportion reads subsampled):
Low (25% reads)
Medium (50% reads)
High (no subsampling)
A_243_14
B98_509
HC_32
M_120_13
0.0030
0.0025
0.0020
2.5
5.0
7.5
Genome−wide heterozygosity
Mean coverage

**Supplementary Fig. 2. Comparison of genome-wide heterozygosity estimates across different depth of coverage categories.**

**Key for Supplementary Fig. 3.** The plots in this figure are arranged by sample (following the pattern below) and then by super-scaffold, starting from Super-Scaffold0 through to Super-Scaffold50. Each page thus contains the inbreeding tracts plot for each sample for a particular super-scaffold. Sampling locality is shown in parenthesis. AENP: Addo Elephant National Park, KNP: Kruger National Park, HiP: Hluhluwe-iMfolozi Park, MNP: Mokala National Park

| | |
|---|---|
| A_243_14 (AENP) | A_251_14 (AENP) |
| A_264_14 (AENP) | A_268_14 (AENP) |
| A_87_13 (AENP) | B98_161 (KNP) |
| B98_284 (KNP) | B98_289 (KNP) |
| B98_324 (KNP) | B98_340 (KNP) |
| B98_361 (KNP) | B98_395 (KNP) |
| B98_396 (KNP) | B98_509 (KNP) |
| B98_546 (KNP) | B98_547 (KNP) |
| B98_574 (KNP) | B98_579 (KNP) |
| B98_586 (KNP) | B98_597 (KNP) |
| HA_1 (HiP) | HA_41 (HiP) |
| HA_43 (HiP) | HB_31 (HiP) |
| HB_39 (HiP) | HC_11 (HiP) |
| HC_15 (HiP) | HC_17 (HiP) |
| HC_18 (HiP) | HC_20 (HiP) |
| HC_30 (HiP) | HC_32 (HiP) |
| HC_40 (HiP) | HC_41 (HiP) |
| HC_48 (HiP) | M_120_13 (MNP) |
| M_12_12 (MNP) | M_301_14 (MNP) |
| M_306_14 (MNP) | M_47_14 (MNP) |

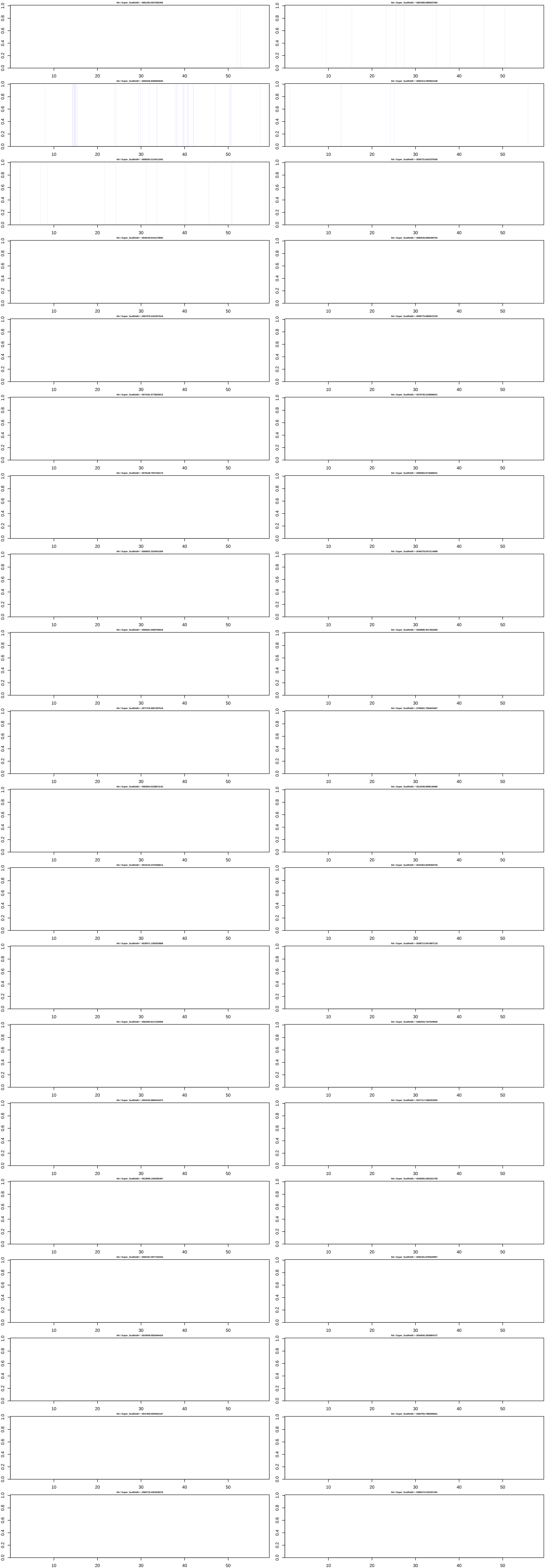

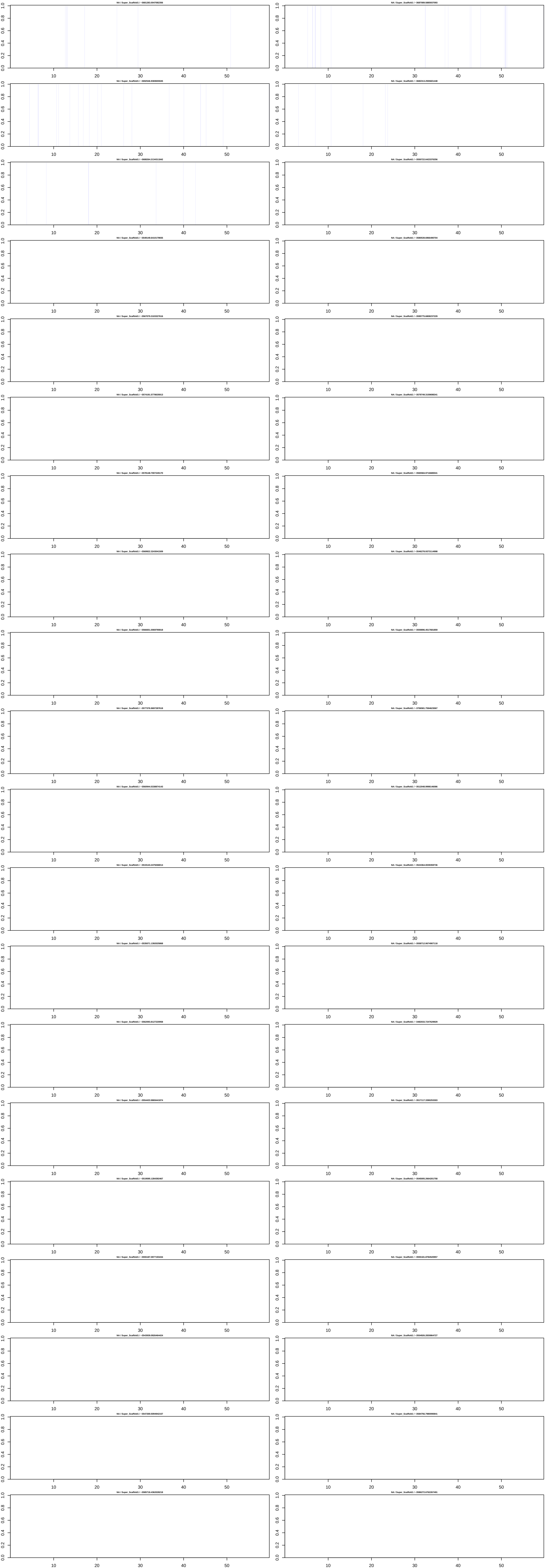

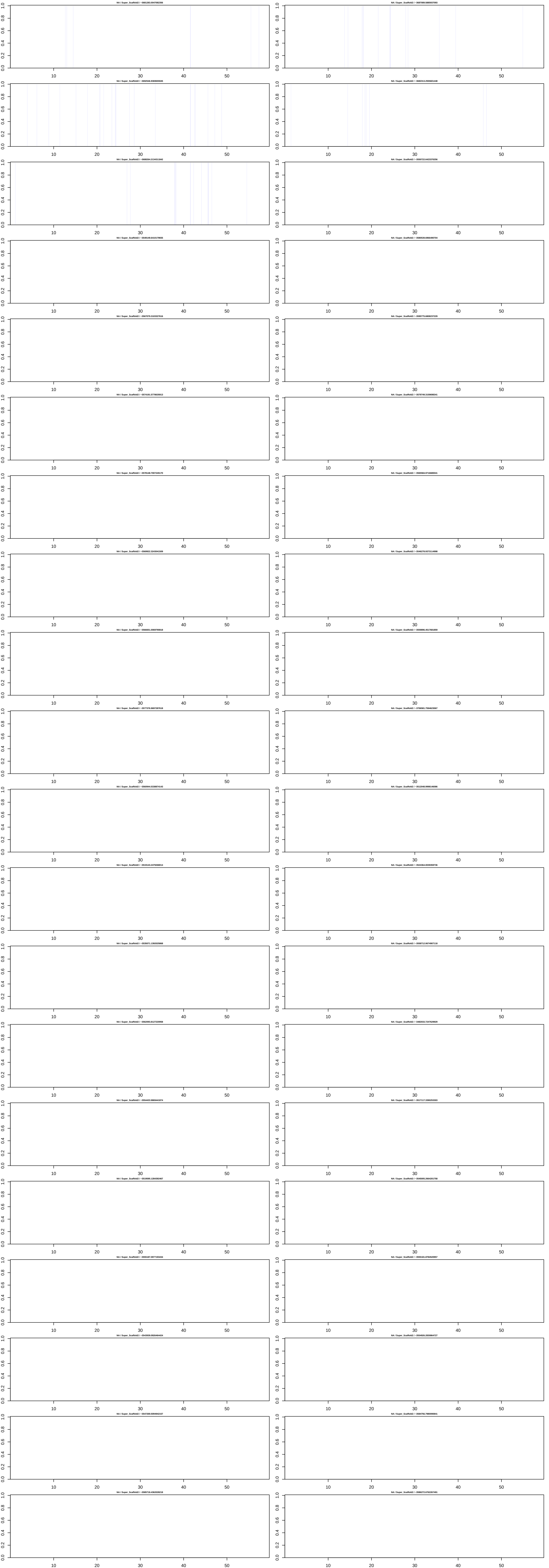
NA / Super_Scaffold2 / −3681283.0047082356
NA / Super_Scaffold2 / −3687089.5885937093
NA / Super_Scaffold2 / −3692546.9369655945
NA / Super_Scaffold2 / −3682313.2555601448
NA / Super_Scaffold2 / −3688264.2134311842
NA / Super_Scaffold2 / −3550722.6423370256
NA / Super_Scaffold2 / −3549149.6410179655
NA / Super_Scaffold2 / −3580539.6966490704
NA / Super_Scaffold2 / −3567079.3103327616
NA / Super_Scaffold2 / −3595775.6808237229
NA / Super_Scaffold2 / −3574191.5778635913
NA / Super_Scaffold2 / −3579749.3159698341
NA / Super_Scaffold2 / −3578148.7007245170
NA / Super_Scaffold2 / −3565584.9716680041
NA / Super_Scaffold2 / −3569922.3243041509
NA / Super_Scaffold2 / −3546278.9373114998
NA / Super_Scaffold2 / −3566651.0069790818
NA / Super_Scaffold2 / −3559896.4517681859
NA / Super_Scaffold2 / −3577376.9697287618
NA / Super_Scaffold2 / −3706581.7594623067
NA / Super_Scaffold2 / −3560944.5338874143
NA / Super_Scaffold2 / −3512048.9998146086
NA / Super_Scaffold2 / −3519143.2479268014
NA / Super_Scaffold2 / −3524364.0039359736
NA / Super_Scaffold2 / −3535071.1392025868
NA / Super_Scaffold2 / −3558712.0674907118
NA / Super_Scaffold2 / −3562093.8127220958
NA / Super_Scaffold2 / −3482033.7247629929
NA / Super_Scaffold2 / −3554433.9869441874
NA / Super_Scaffold2 / −3517117.3390253303
NA / Super_Scaffold2 / −3519595.1284392467
NA / Super_Scaffold2 / −3545005.2664201758
NA / Super_Scaffold2 / −3555187.0977193434
NA / Super_Scaffold2 / −3555181.8784520957
NA / Super_Scaffold2 / −3543939.0926464424
NA / Super_Scaffold2 / −3554926.2830864727
NA / Super_Scaffold2 / −3547269.5094942157
NA / Super_Scaffold2 / −3584756.7980090841
NA / Super_Scaffold2 / −3585718.4362028218
NA / Super_Scaffold2 / −3586272.6762267491

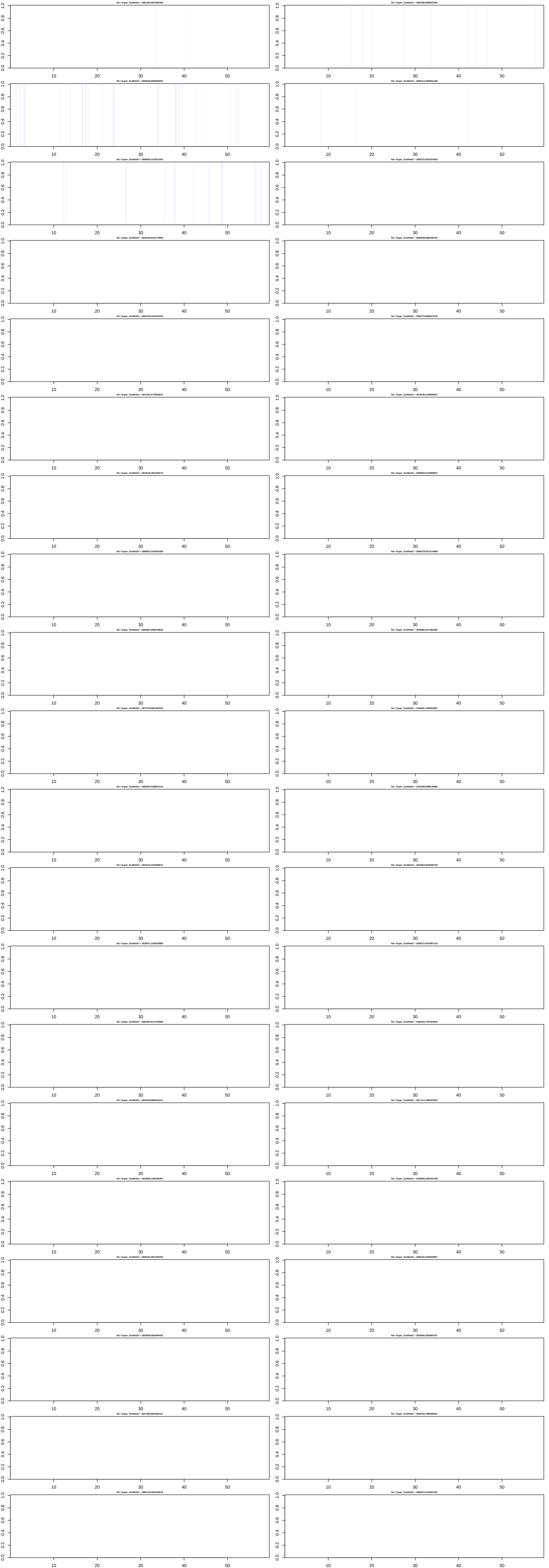

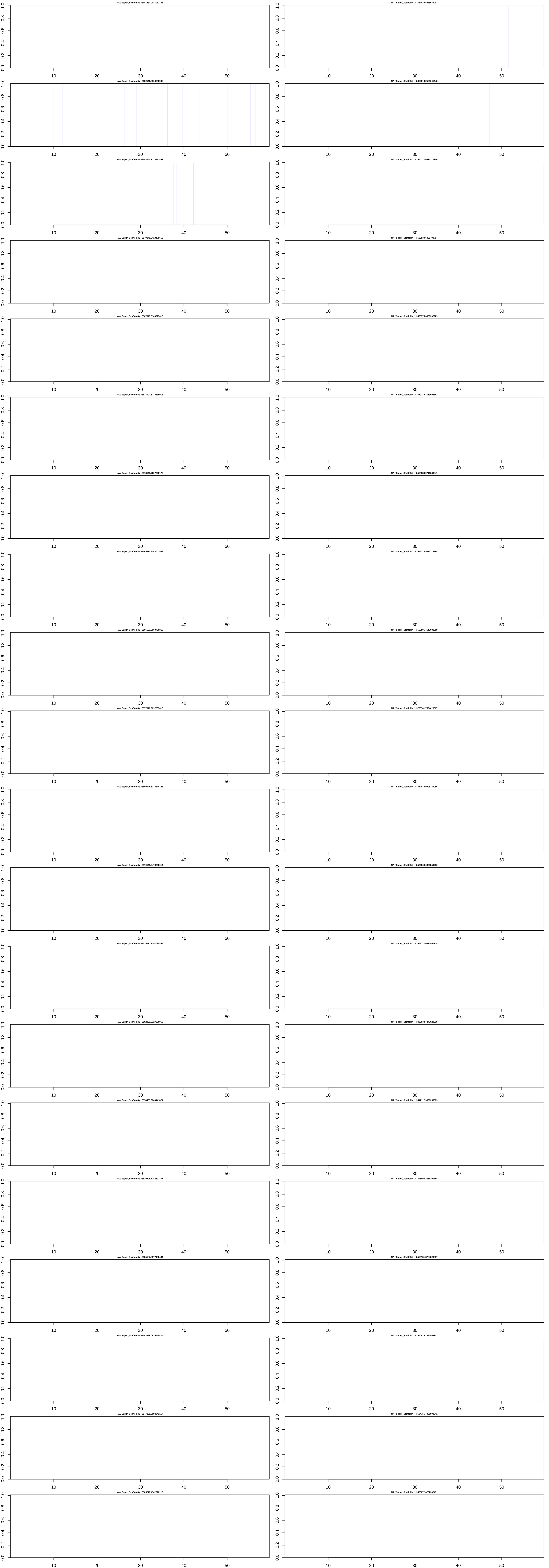

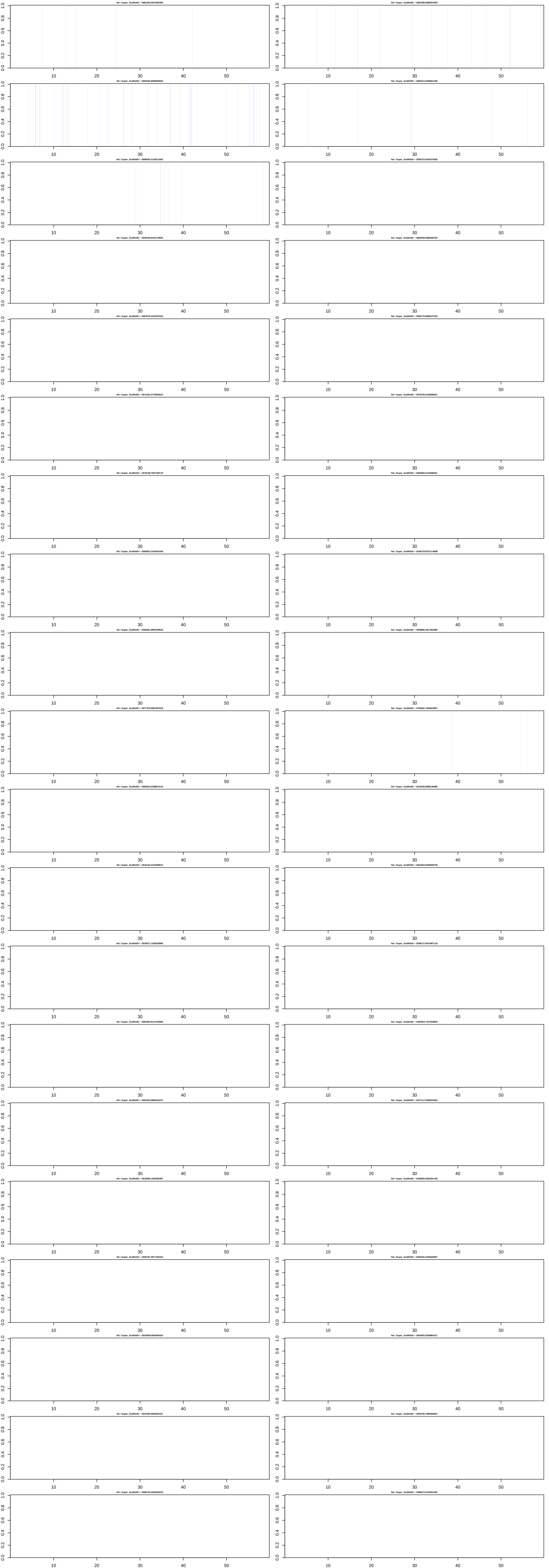

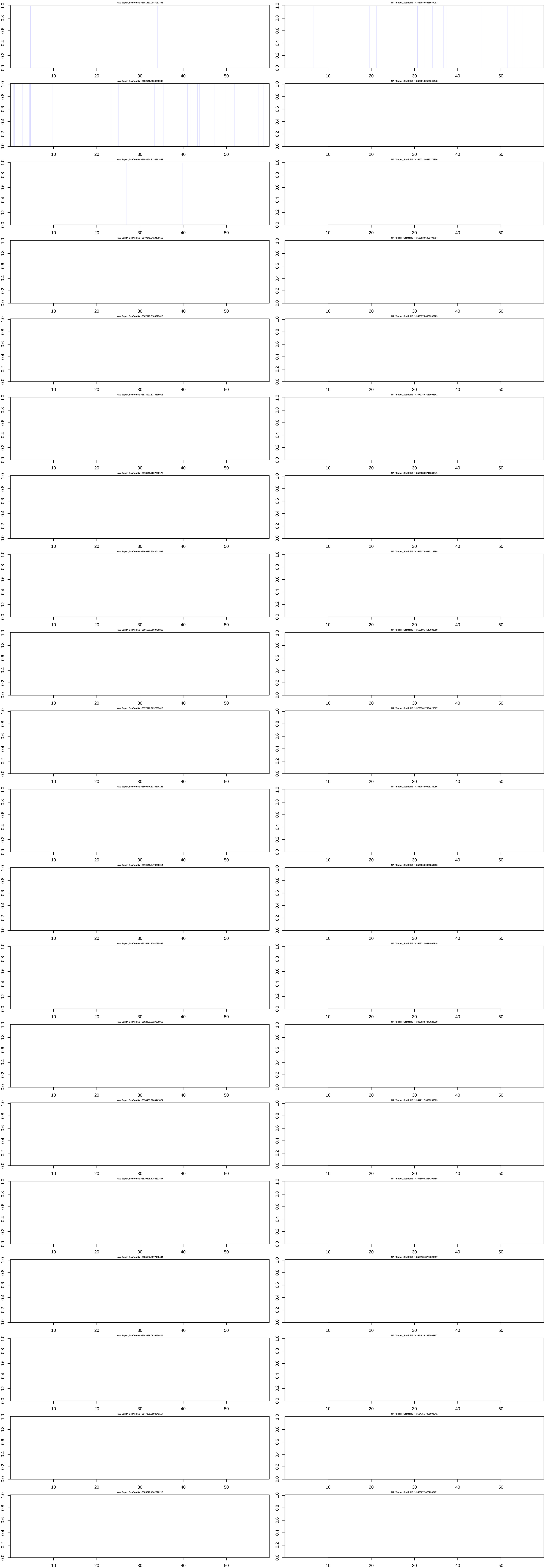

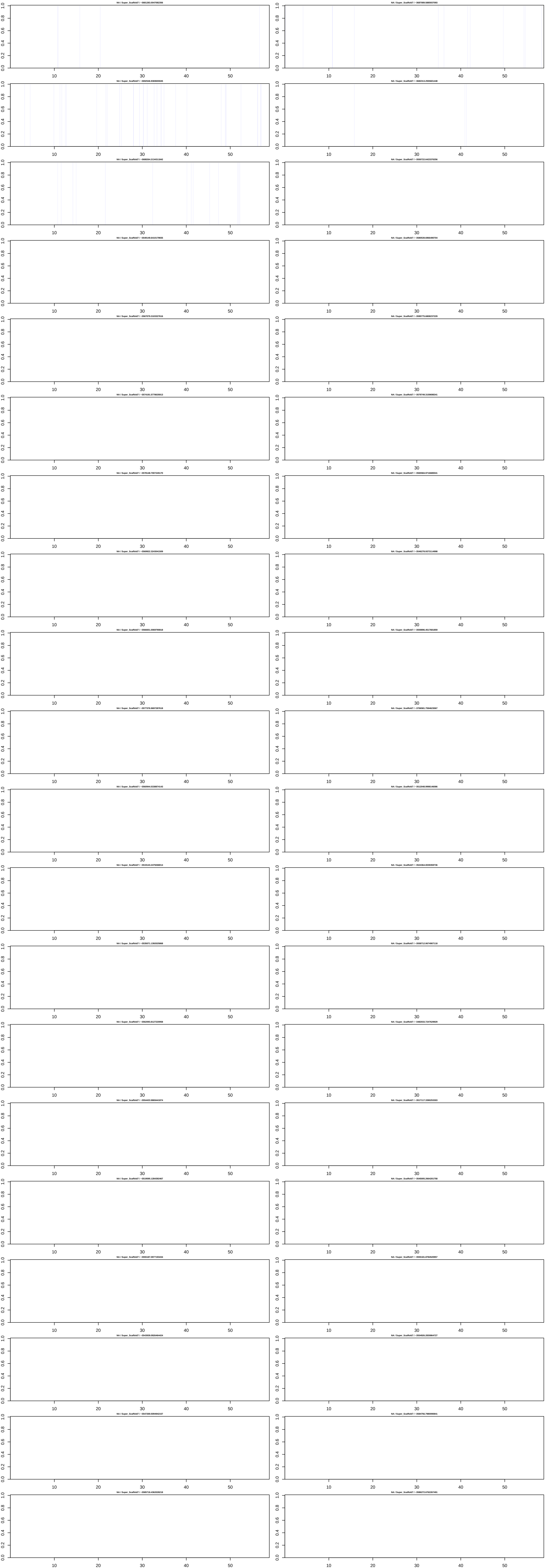

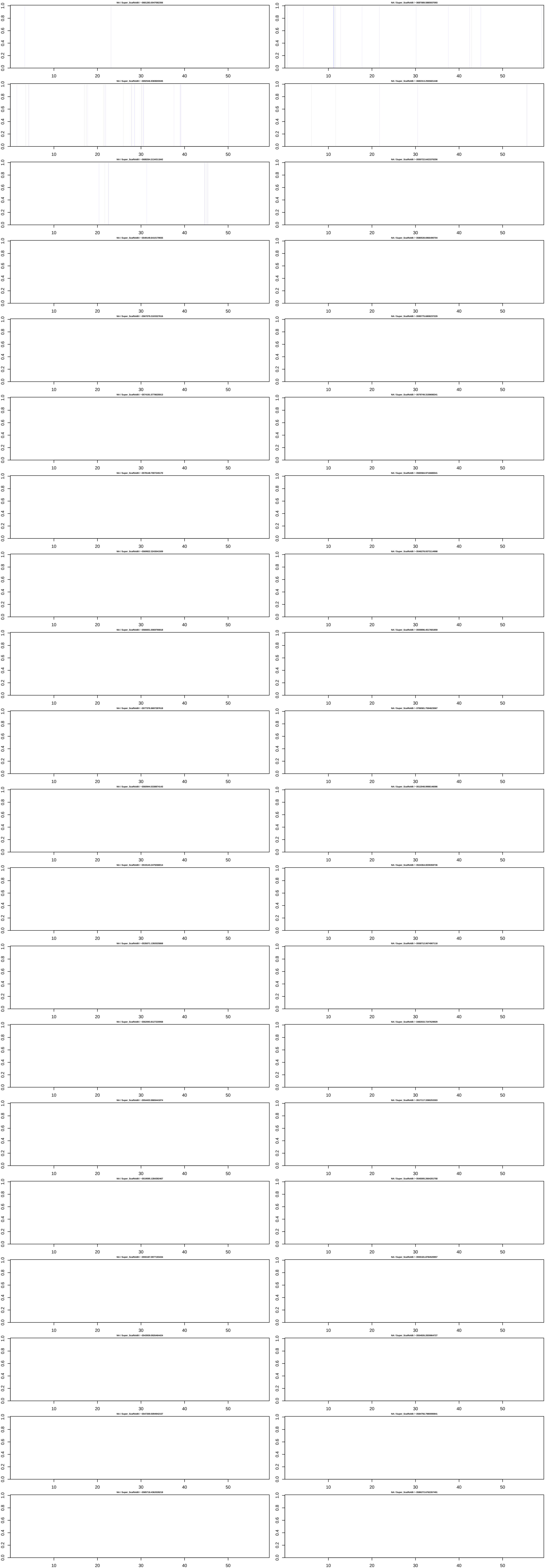

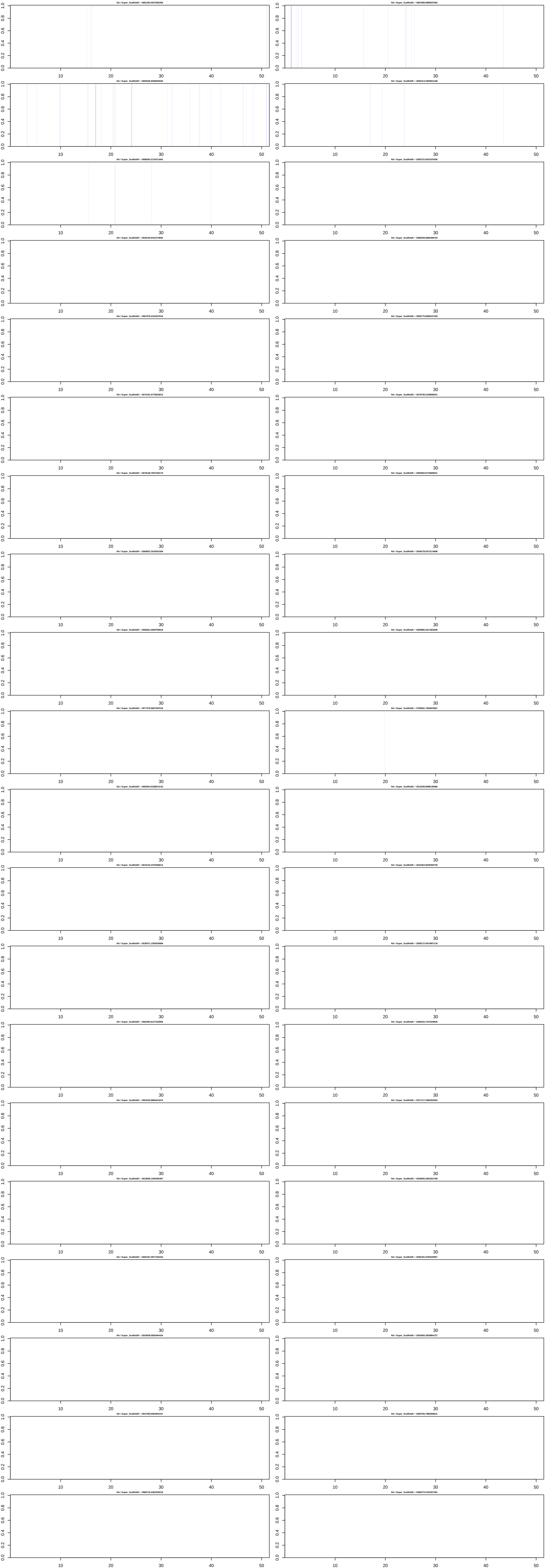

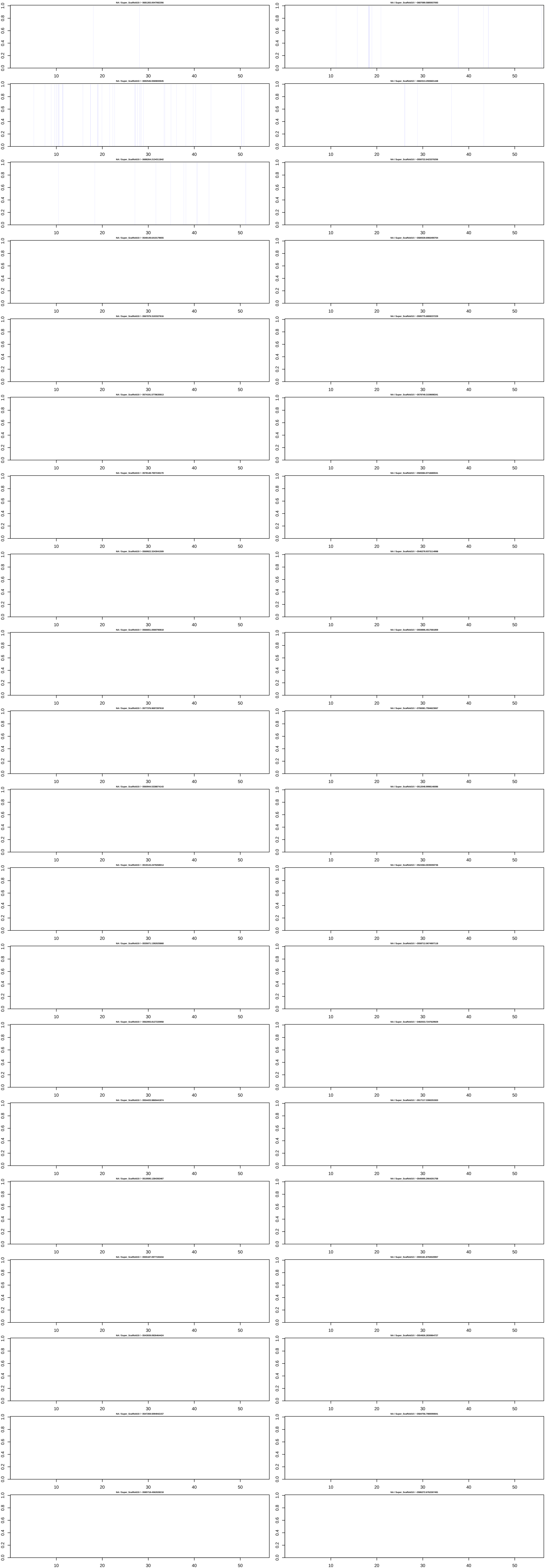

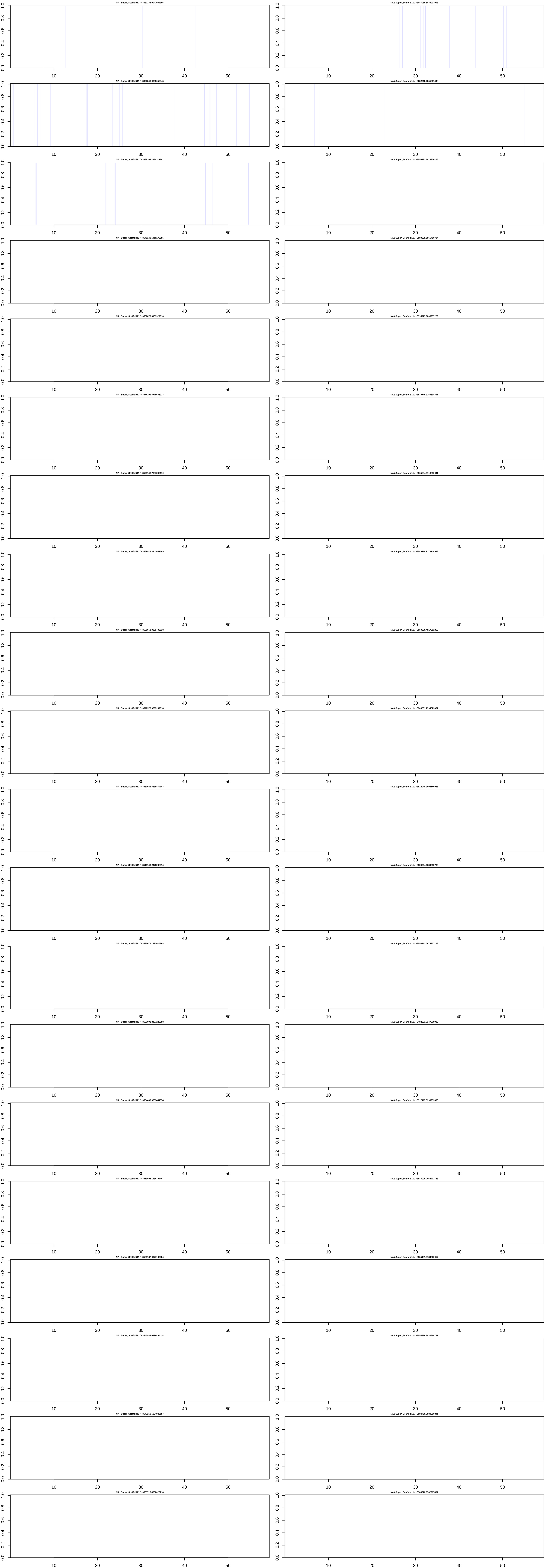

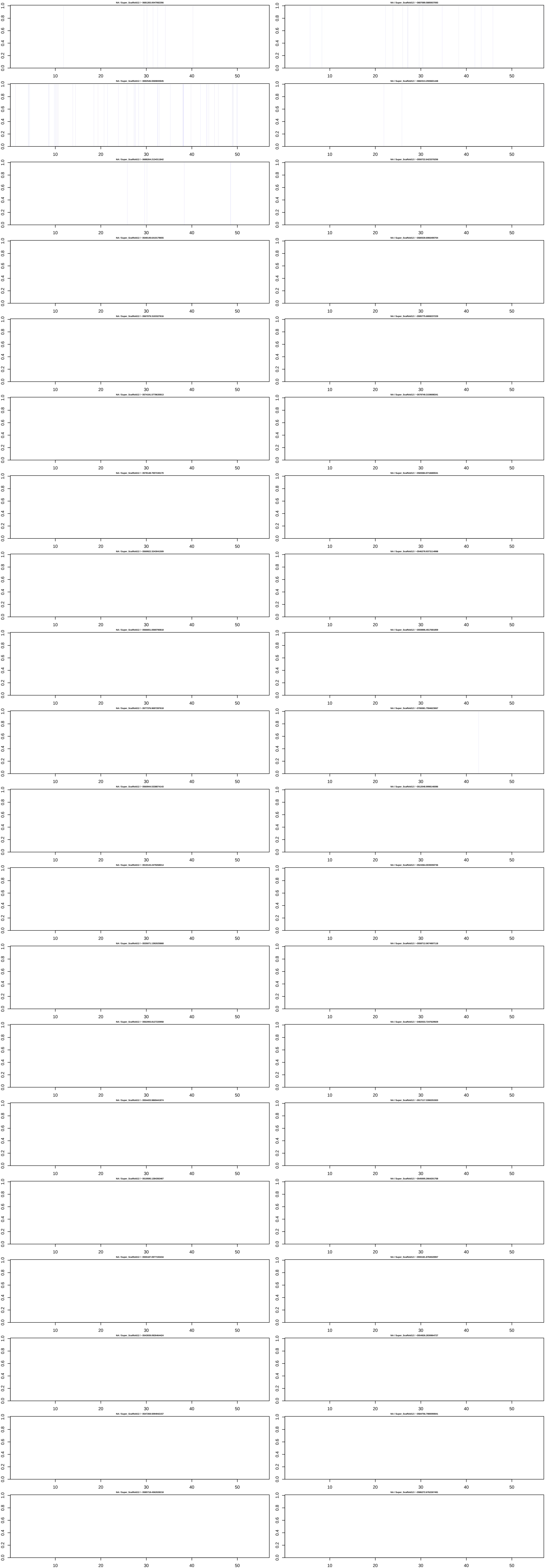

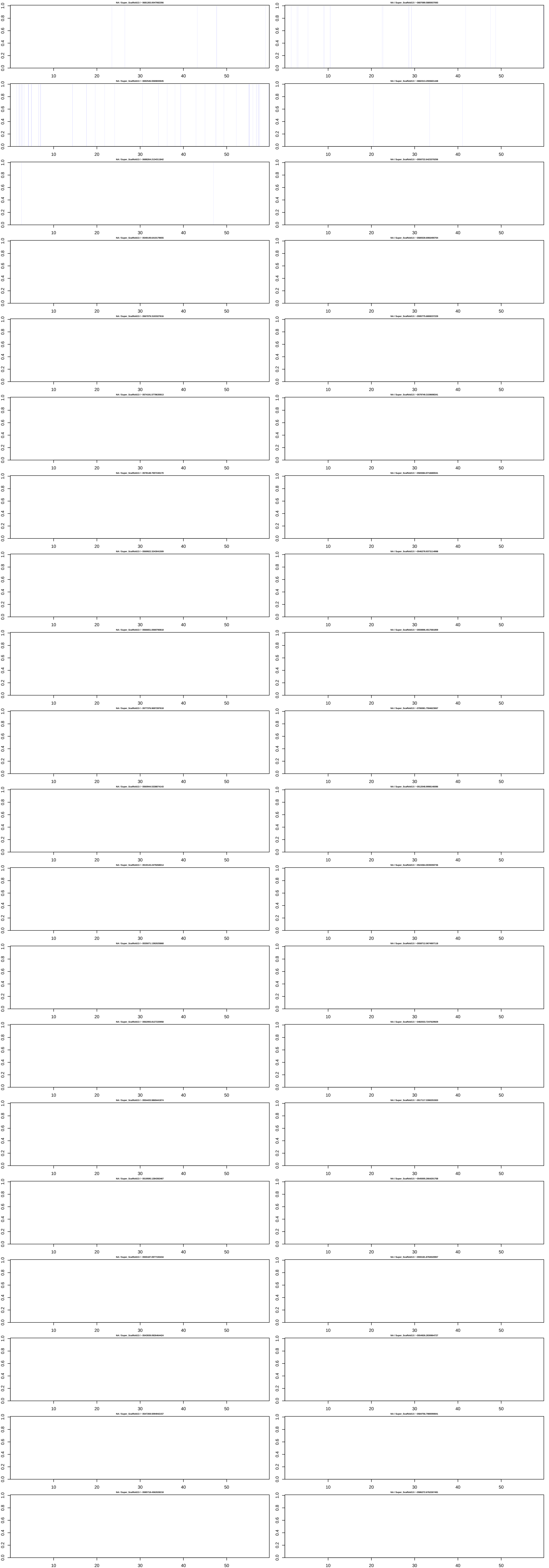

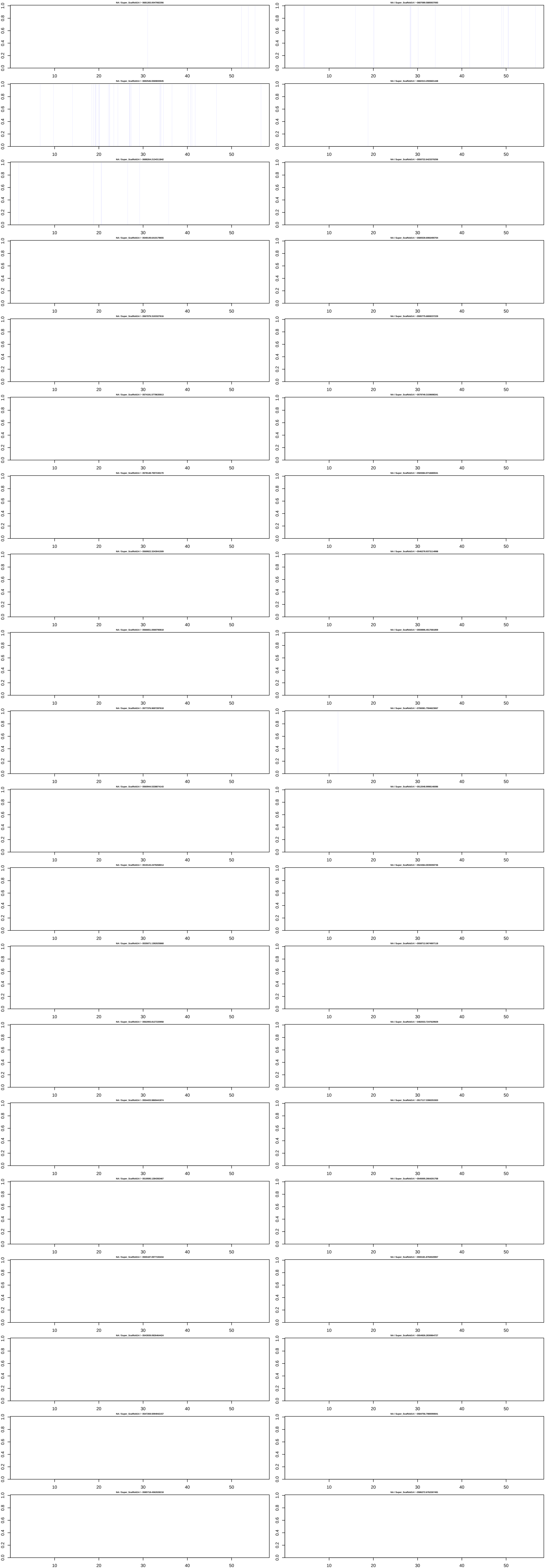
NA / Super_Scaffold14 / −3681283.0047082356
NA / Super_Scaffold14 / −3687089.5885937093
NA / Super_Scaffold14 / −3692546.9369655945
NA / Super_Scaffold14 / −3682313.2555601448
NA / Super_Scaffold14 / −3688264.2134311842
NA / Super_Scaffold14 / −3550722.6423370256
NA / Super_Scaffold14 / −3549149.6410179655
NA / Super_Scaffold14 / −3580539.6966490704
NA / Super_Scaffold14 / −3567079.3103327616
NA / Super_Scaffold14 / −3595775.6808237229
NA / Super_Scaffold14 / −3574191.5778635913
NA / Super_Scaffold14 / −3579749.3159698341
NA / Super_Scaffold14 / −3578148.7007245170
NA / Super_Scaffold14 / −3565584.9716680041
NA / Super_Scaffold14 / −3569922.3243041509
NA / Super_Scaffold14 / −3546278.9373114998
NA / Super_Scaffold14 / −3566651.0069790818
NA / Super_Scaffold14 / −3559896.4517681859
NA / Super_Scaffold14 / −3577376.9697287618
NA / Super_Scaffold14 / −3706581.7594623067
NA / Super_Scaffold14 / −3560944.5338874143
NA / Super_Scaffold14 / −3512048.9998146086
NA / Super_Scaffold14 / −3519143.2479268014
NA / Super_Scaffold14 / −3524364.0039359736
NA / Super_Scaffold14 / −3535071.1392025868
NA / Super_Scaffold14 / −3558712.0674907118
NA / Super_Scaffold14 / −3562093.8127220958
NA / Super_Scaffold14 / −3482033.7247629929
NA / Super_Scaffold14 / −3554433.9869441874
NA / Super_Scaffold14 / −3517117.3390253303
NA / Super_Scaffold14 / −3519595.1284392467
NA / Super_Scaffold14 / −3545005.2664201758
NA / Super_Scaffold14 / −3555187.0977193434
NA / Super_Scaffold14 / −3555181.8784520957
NA / Super_Scaffold14 / −3543939.0926464424
NA / Super_Scaffold14 / −3554926.2830864727
NA / Super_Scaffold14 / −3547269.5094942157
NA / Super_Scaffold14 / −3584756.7980090841
NA / Super_Scaffold14 / −3585718.4362028218
NA / Super_Scaffold14 / −3586272.6762267491

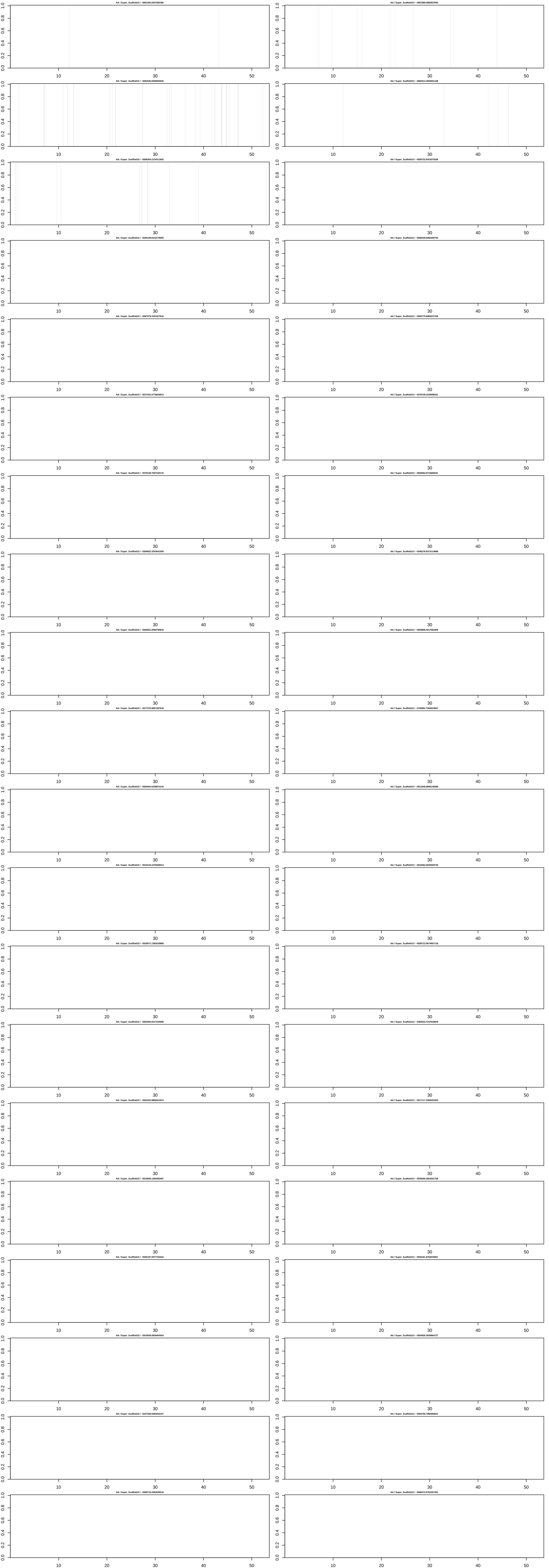

NA / Super_Scaffold15 / −3681283.0047082356
NA / Super_Scaffold15 / −3687089.5885937093
NA / Super_Scaffold15 / −3692546.9369655945
NA / Super_Scaffold15 / −3682313.2555601448
NA / Super_Scaffold15 / −3688264.2134311842
NA / Super_Scaffold15 / −3550722.6423370256
NA / Super_Scaffold15 / −3549149.6410179655
NA / Super_Scaffold15 / −3580539.6966490704
NA / Super_Scaffold15 / −3567079.3103327616
NA / Super_Scaffold15 / −3595775.6808237229
NA / Super_Scaffold15 / −3574191.5778635913
NA / Super_Scaffold15 / −3579749.3159698341
NA / Super_Scaffold15 / −3578148.7007245170
NA / Super_Scaffold15 / −3565584.9716680041
NA / Super_Scaffold15 / −3569922.3243041509
NA / Super_Scaffold15 / −3546278.9373114998
NA / Super_Scaffold15 / −3566651.0069790818
NA / Super_Scaffold15 / −3559896.4517681859
NA / Super_Scaffold15 / −3577376.9697287618
NA / Super_Scaffold15 / −3706581.7594623067
NA / Super_Scaffold15 / −3560944.5338874143
NA / Super_Scaffold15 / −3512048.9998146086
NA / Super_Scaffold15 / −3519143.2479268014
NA / Super_Scaffold15 / −3524364.0039359736
NA / Super_Scaffold15 / −3535071.1392025868
NA / Super_Scaffold15 / −3558712.0674907118
NA / Super_Scaffold15 / −3562093.8127220958
NA / Super_Scaffold15 / −3482033.7247629929
NA / Super_Scaffold15 / −3554433.9869441874
NA / Super_Scaffold15 / −3517117.3390253303
NA / Super_Scaffold15 / −3519595.1284392467
NA / Super_Scaffold15 / −3545005.2664201758
NA / Super_Scaffold15 / −3555187.0977193434
NA / Super_Scaffold15 / −3555181.8784520957
NA / Super_Scaffold15 / −3543939.0926464424
NA / Super_Scaffold15 / −3554926.2830864727
NA / Super_Scaffold15 / −3547269.5094942157
NA / Super_Scaffold15 / −3584756.7980090841
NA / Super_Scaffold15 / −3585718.4362028218
NA / Super_Scaffold15 / −3586272.6762267491

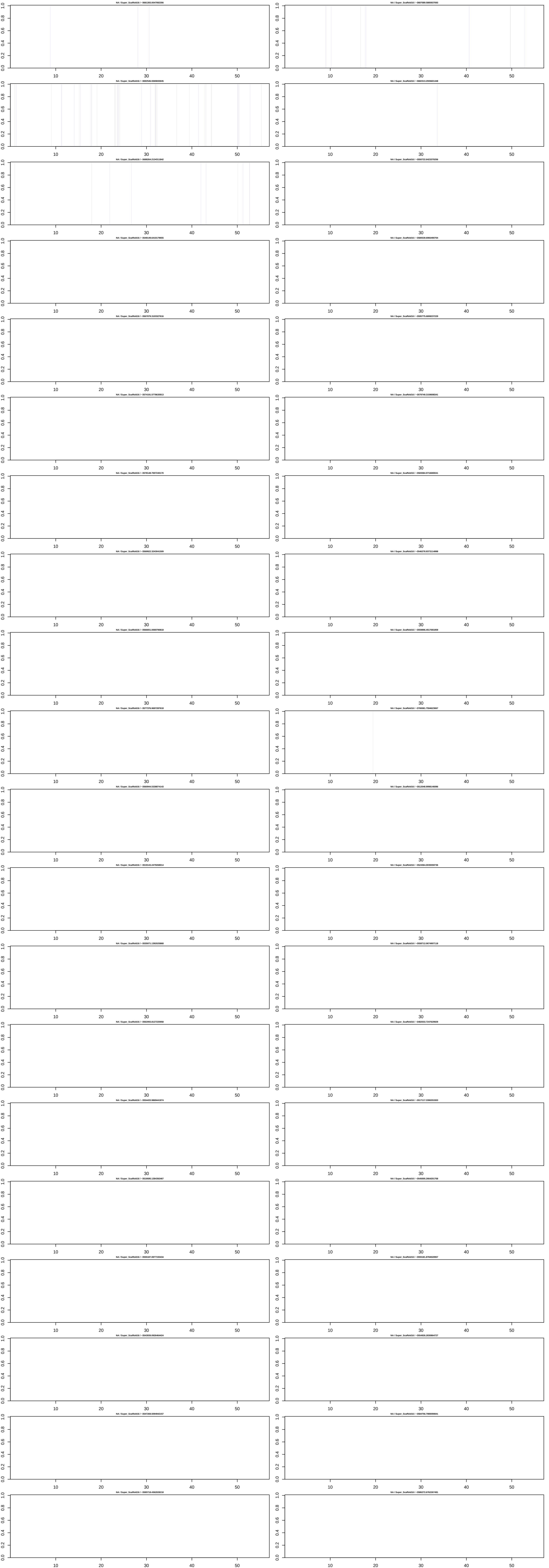

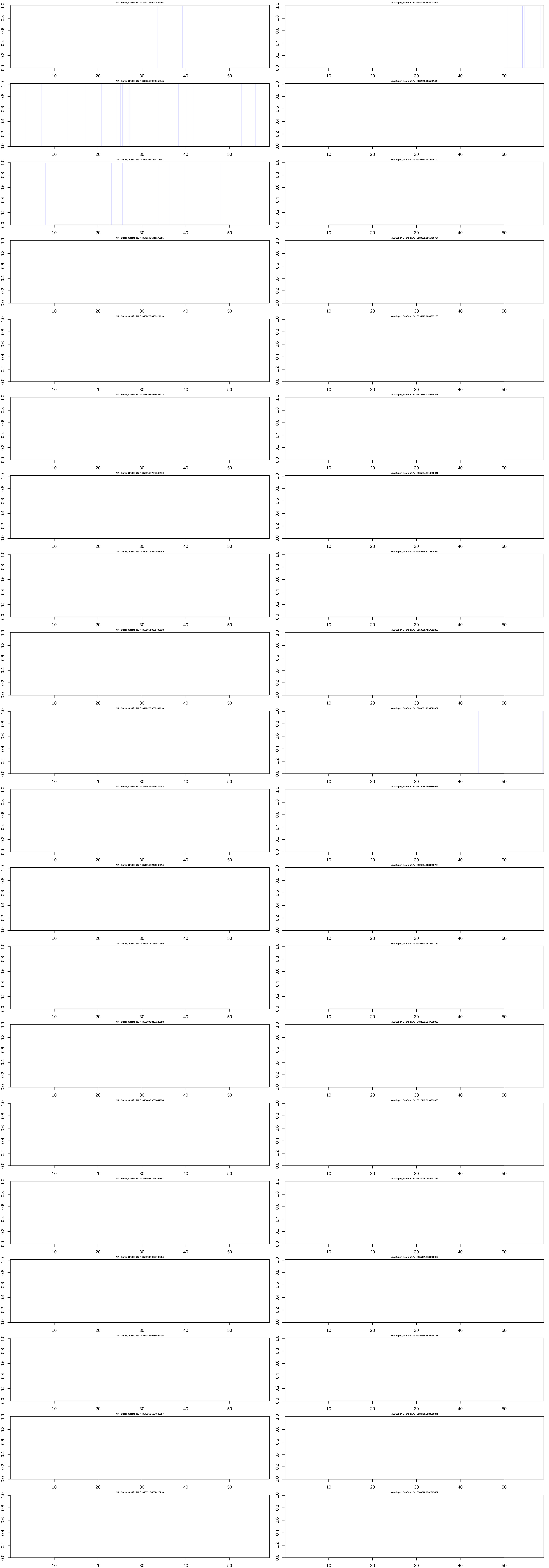

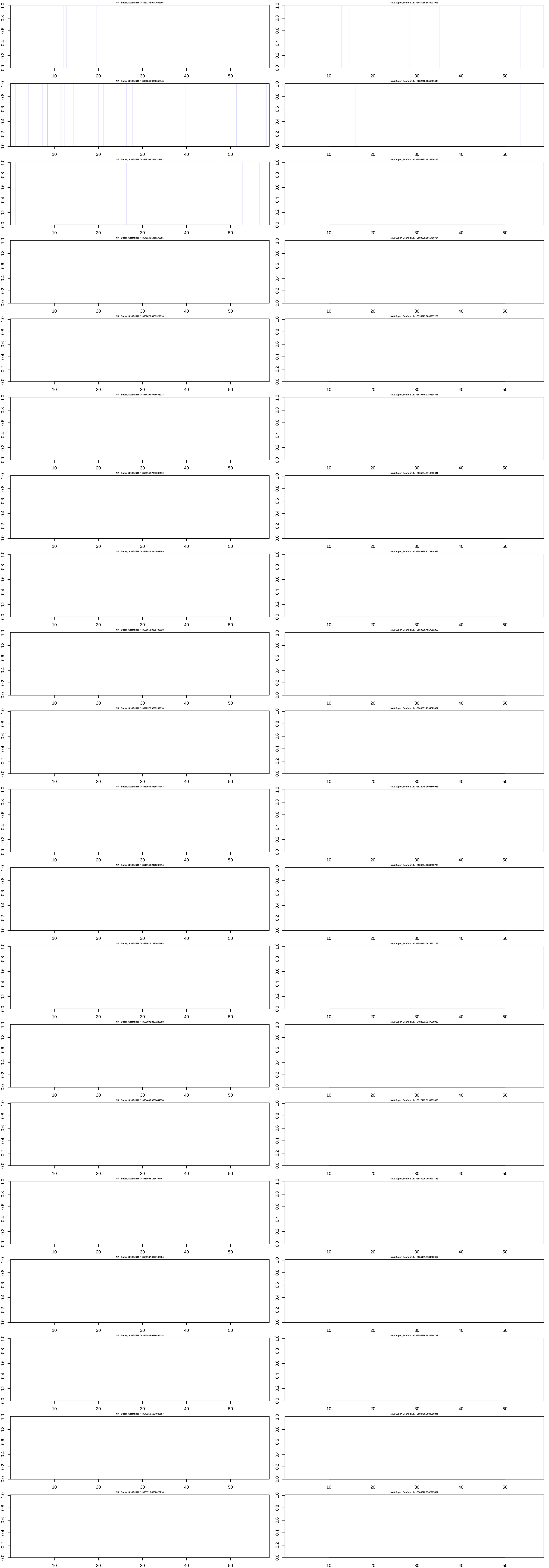

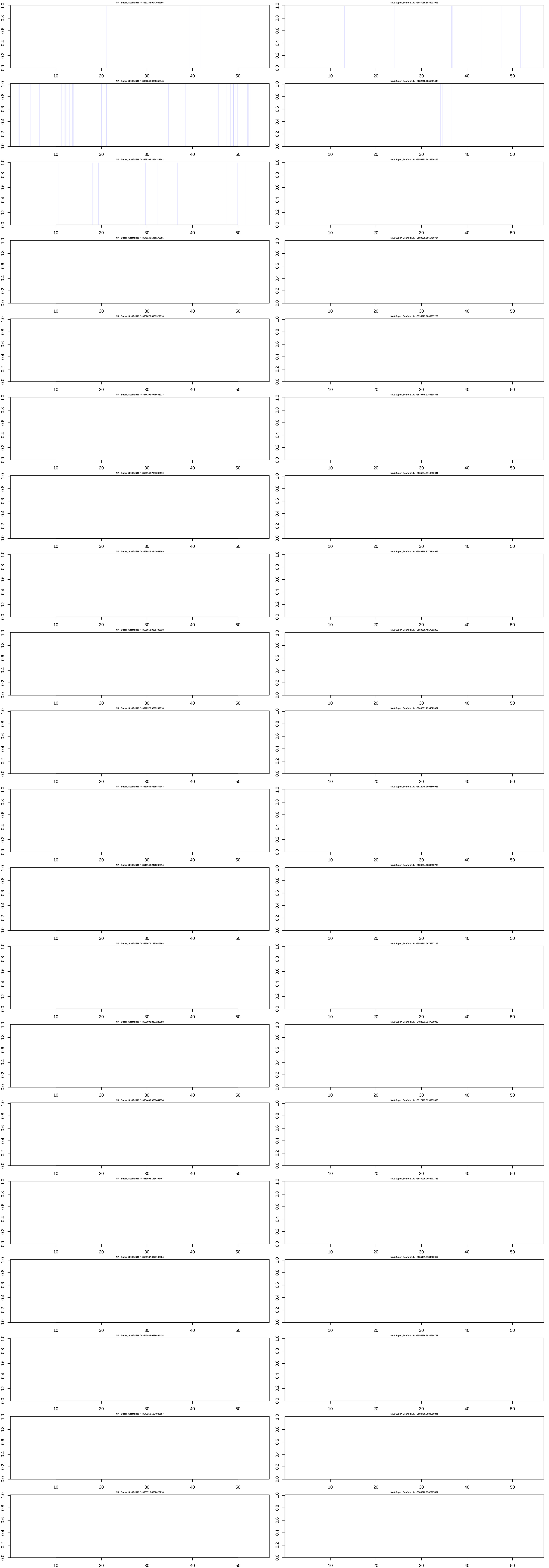

NA / Super_Scaffold19 / −3681283.0047082356
NA / Super_Scaffold19 / −3692546.9369655945

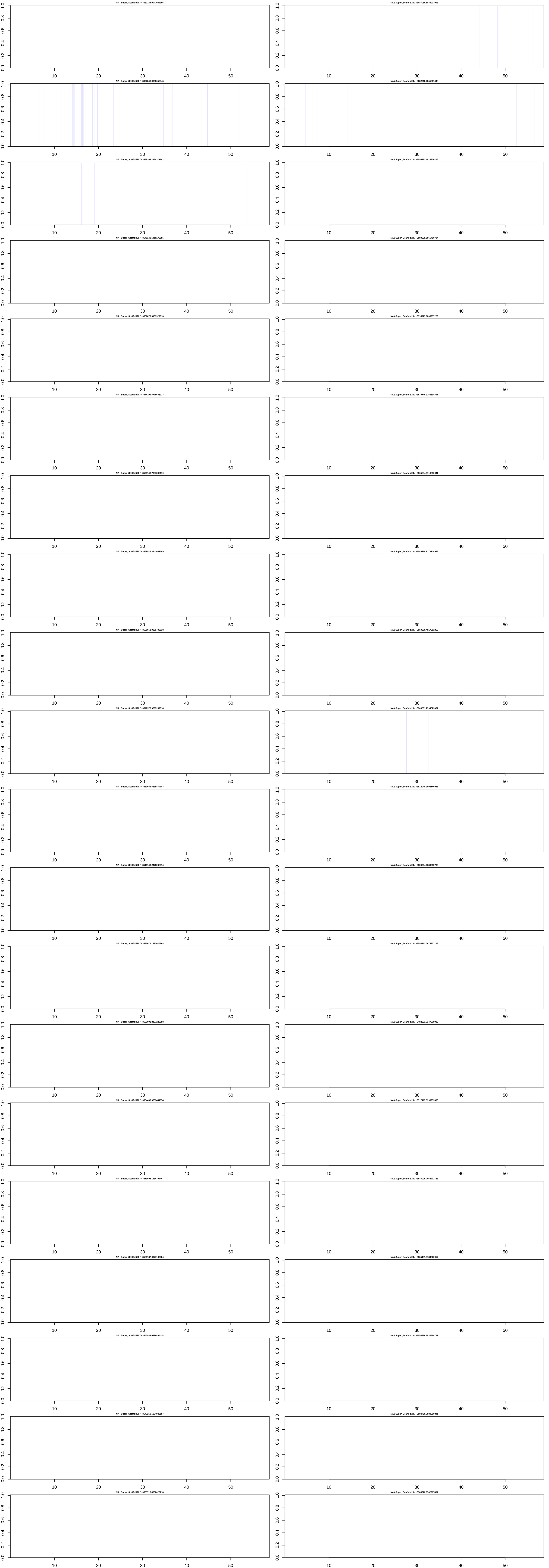
NA / Super_Scaffold20 / −3681283.0047082356
NA / Super_Scaffold20 / −3687089.5885937093
NA / Super_Scaffold20 / −3692546.9369655945
NA / Super_Scaffold20 / −3682313.2555601448
NA / Super_Scaffold20 / −3688264.2134311842
NA / Super_Scaffold20 / −3550722.6423370256
NA / Super_Scaffold20 / −3549149.6410179655
NA / Super_Scaffold20 / −3580539.6966490704
NA / Super_Scaffold20 / −3567079.3103327616
NA / Super_Scaffold20 / −3595775.6808237229
NA / Super_Scaffold20 / −3574191.5778635913
NA / Super_Scaffold20 / −3579749.3159698341
NA / Super_Scaffold20 / −3578148.7007245170
NA / Super_Scaffold20 / −3565584.9716680041
NA / Super_Scaffold20 / −3569922.3243041509
NA / Super_Scaffold20 / −3546278.9373114998
NA / Super_Scaffold20 / −3566651.0069790818
NA / Super_Scaffold20 / −3559896.4517681859
NA / Super_Scaffold20 / −3577376.9697287618
NA / Super_Scaffold20 / −3706581.7594623067
NA / Super_Scaffold20 / −3560944.5338874143
NA / Super_Scaffold20 / −3512048.9998146086
NA / Super_Scaffold20 / −3519143.2479268014
NA / Super_Scaffold20 / −3524364.0039359736
NA / Super_Scaffold20 / −3535071.1392025868
NA / Super_Scaffold20 / −3558712.0674907118
NA / Super_Scaffold20 / −3562093.8127220958
NA / Super_Scaffold20 / −3482033.7247629929
NA / Super_Scaffold20 / −3554433.9869441874
NA / Super_Scaffold20 / −3517117.3390253303
NA / Super_Scaffold20 / −3519595.1284392467
NA / Super_Scaffold20 / −3545005.2664201758
NA / Super_Scaffold20 / −3555187.0977193434
NA / Super_Scaffold20 / −3555181.8784520957
NA / Super_Scaffold20 / −3543939.0926464424
NA / Super_Scaffold20 / −3554926.2830864727
NA / Super_Scaffold20 / −3547269.5094942157
NA / Super_Scaffold20 / −3584756.7980090841
NA / Super_Scaffold20 / −3585718.4362028218
NA / Super_Scaffold20 / −3586272.6762267491

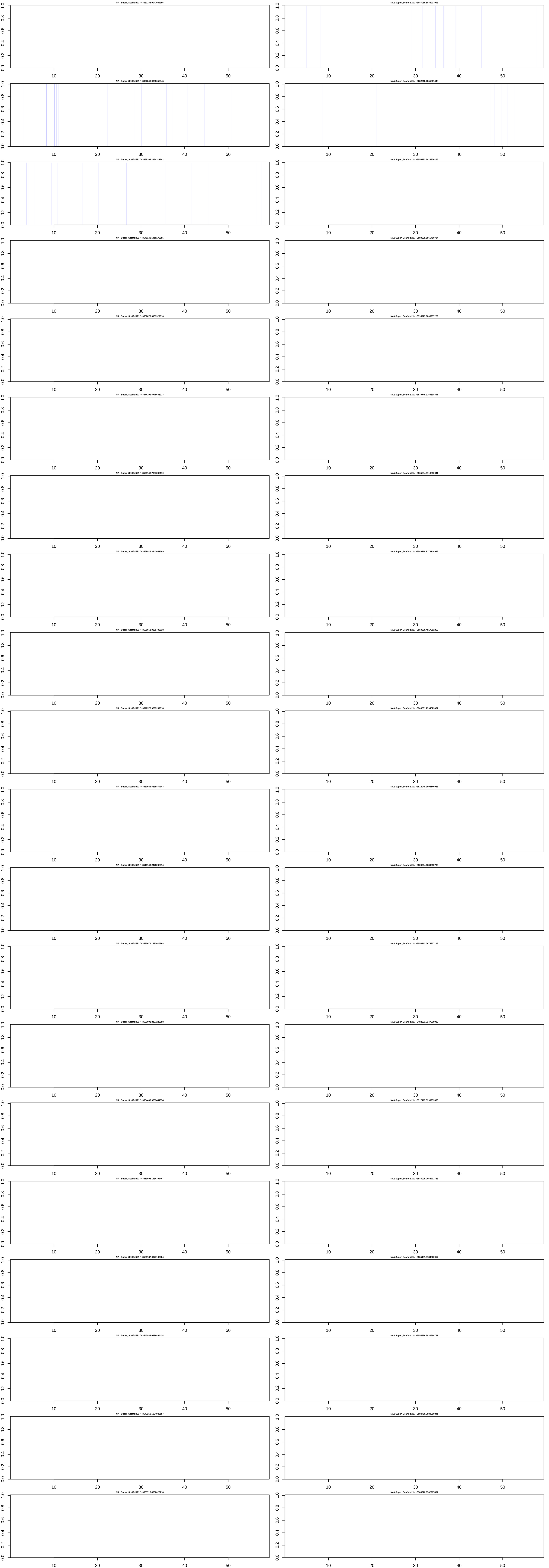

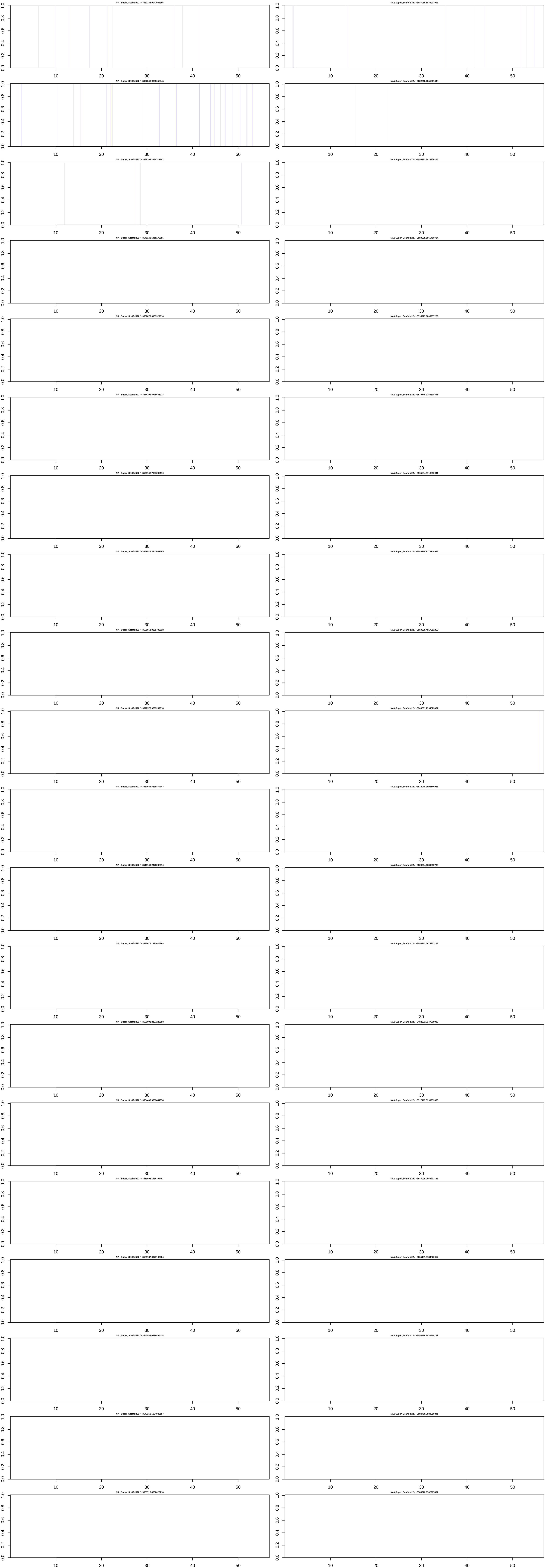

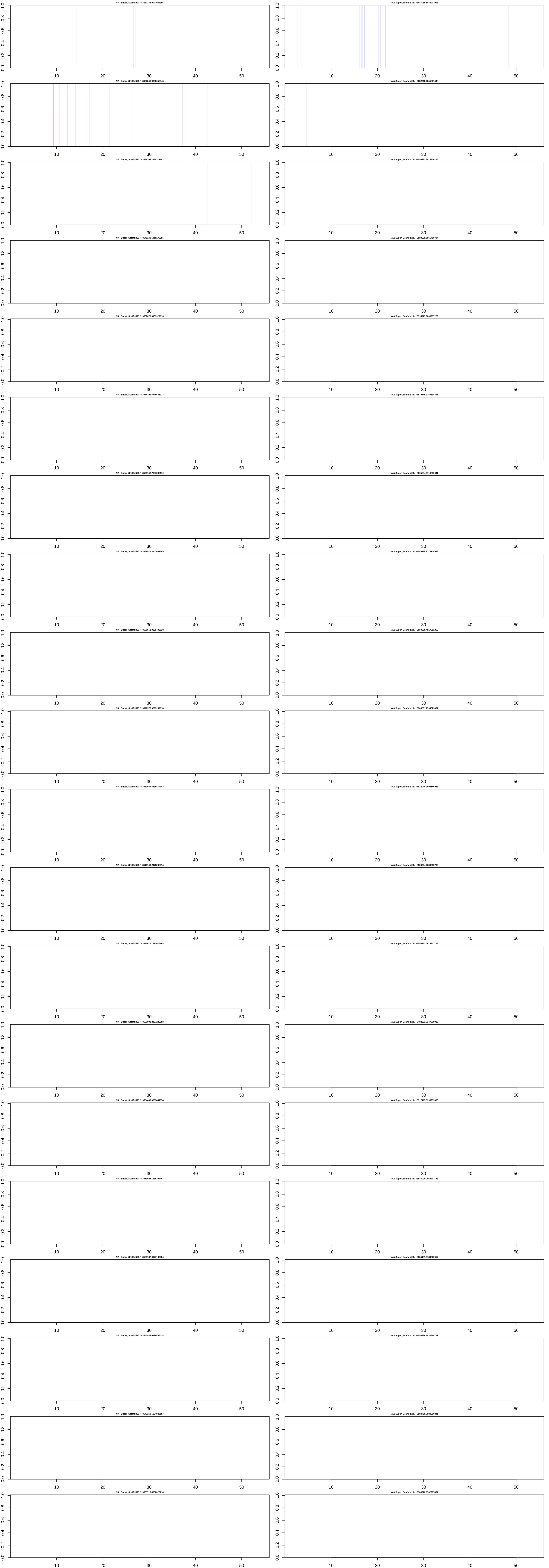

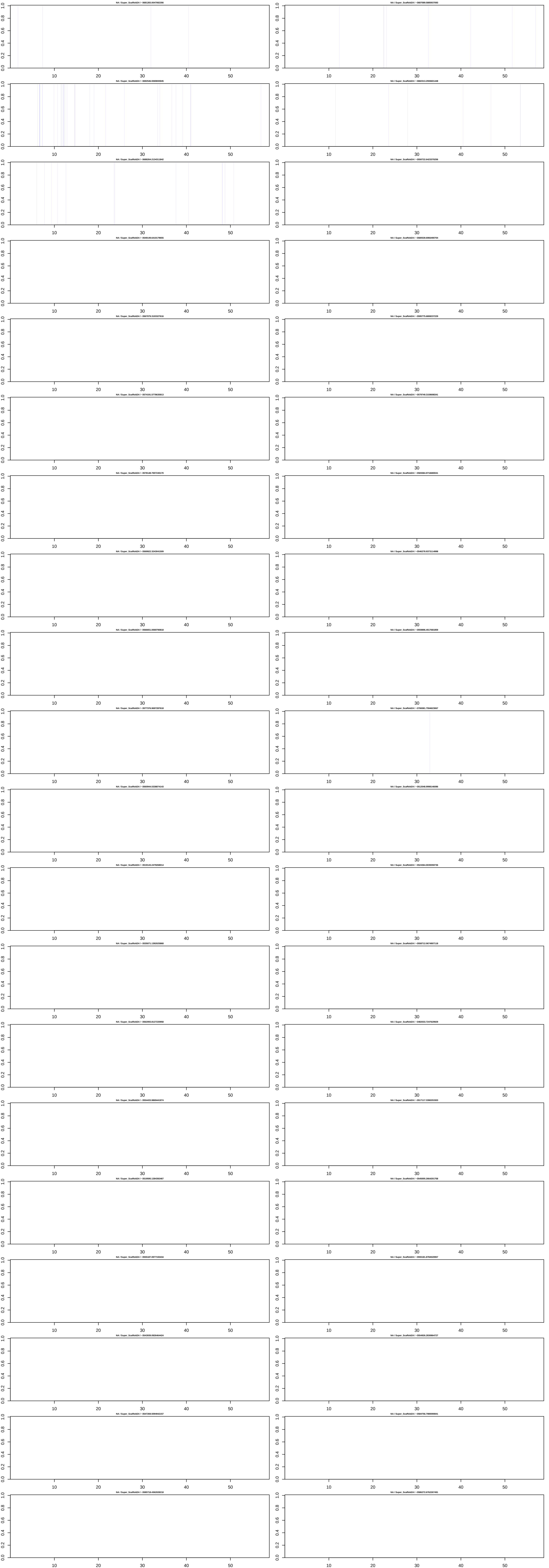

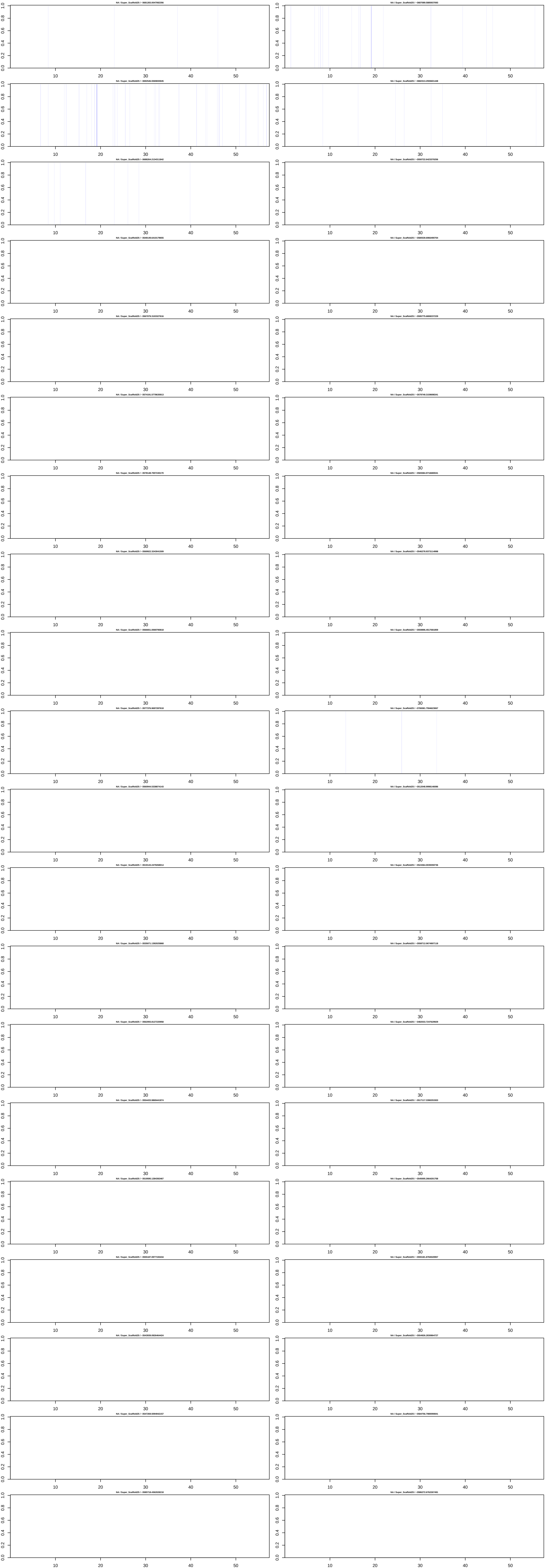
NA / Super_Scaffold25 / −3681283.0047082356
NA / Super_Scaffold25 / −3687089.5885937093
NA / Super_Scaffold25 / −3692546.9369655945
NA / Super_Scaffold25 / −3682313.2555601448
NA / Super_Scaffold25 / −3688264.2134311842
NA / Super_Scaffold25 / −3550722.6423370256
NA / Super_Scaffold25 / −3549149.6410179655
NA / Super_Scaffold25 / −3580539.6966490704
NA / Super_Scaffold25 / −3567079.3103327616
NA / Super_Scaffold25 / −3595775.6808237229
NA / Super_Scaffold25 / −3574191.5778635913
NA / Super_Scaffold25 / −3579749.3159698341
NA / Super_Scaffold25 / −3578148.7007245170
NA / Super_Scaffold25 / −3565584.9716680041
NA / Super_Scaffold25 / −3569922.3243041509
NA / Super_Scaffold25 / −3546278.9373114998
NA / Super_Scaffold25 / −3566651.0069790818
NA / Super_Scaffold25 / −3559896.4517681859
NA / Super_Scaffold25 / −3577376.9697287618
NA / Super_Scaffold25 / −3706581.7594623067
NA / Super_Scaffold25 / −3560944.5338874143
NA / Super_Scaffold25 / −3512048.9998146086
NA / Super_Scaffold25 / −3519143.2479268014
NA / Super_Scaffold25 / −3524364.0039359736
NA / Super_Scaffold25 / −3535071.1392025868
NA / Super_Scaffold25 / −3558712.0674907118
NA / Super_Scaffold25 / −3562093.8127220958
NA / Super_Scaffold25 / −3482033.7247629929
NA / Super_Scaffold25 / −3554433.9869441874
NA / Super_Scaffold25 / −3517117.3390253303
NA / Super_Scaffold25 / −3519595.1284392467
NA / Super_Scaffold25 / −3545005.2664201758
NA / Super_Scaffold25 / −3555187.0977193434
NA / Super_Scaffold25 / −3555181.8784520957
NA / Super_Scaffold25 / −3543939.0926464424
NA / Super_Scaffold25 / −3554926.2830864727
NA / Super_Scaffold25 / −3547269.5094942157
NA / Super_Scaffold25 / −3584756.7980090841
NA / Super_Scaffold25 / −3585718.4362028218
NA / Super_Scaffold25 / −3586272.6762267491

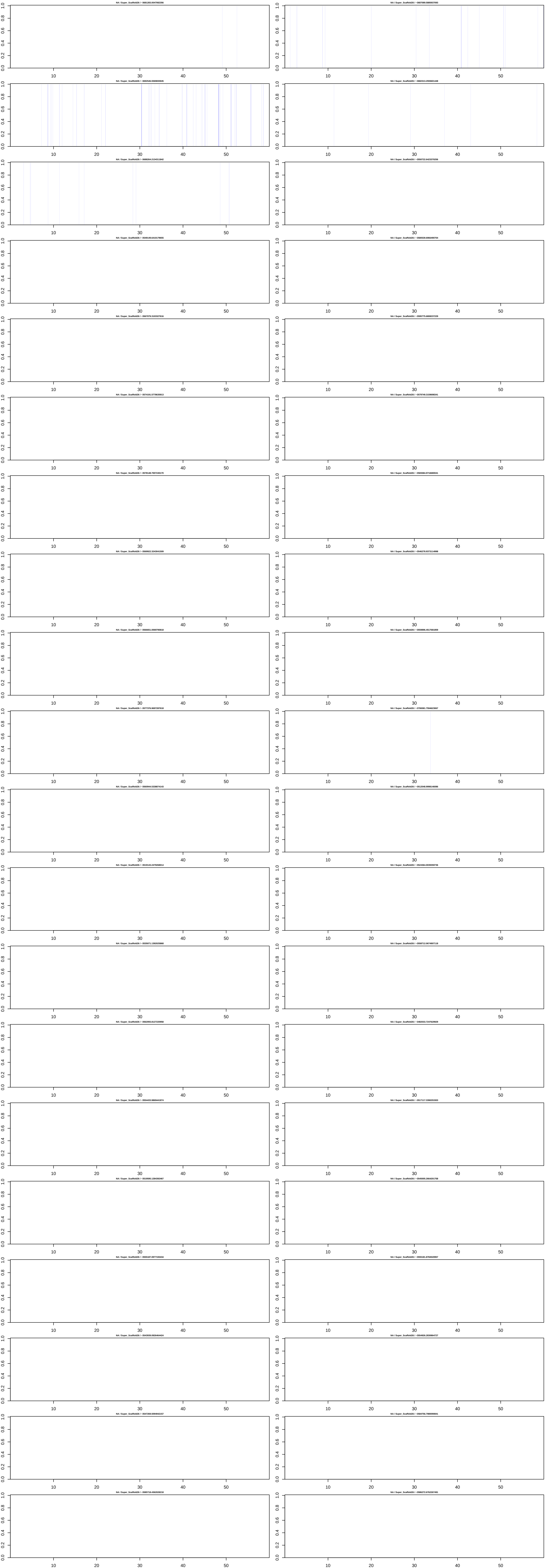

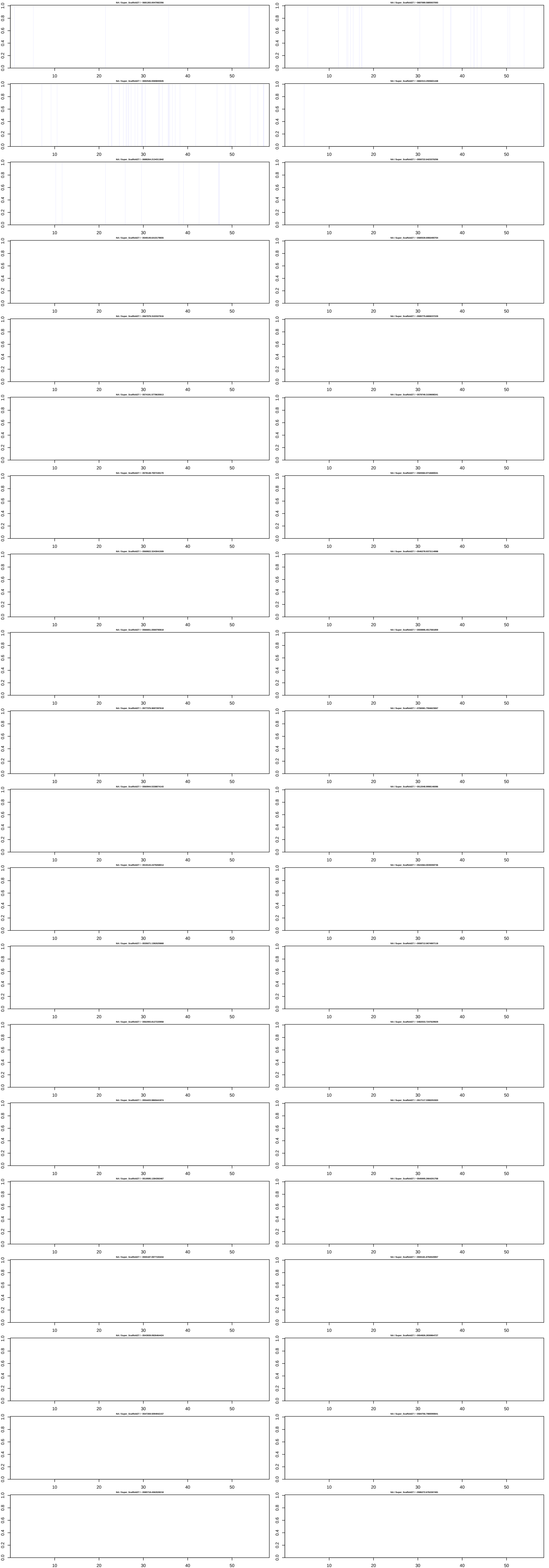
NA / Super_Scaffold27 / −3681283.0047082356
NA / Super_Scaffold27 / −3687089.5885937093
NA / Super_Scaffold27 / −3692546.9369655945
NA / Super_Scaffold27 / −3682313.2555601448
NA / Super_Scaffold27 / −3688264.2134311842
NA / Super_Scaffold27 / −3550722.6423370256
NA / Super_Scaffold27 / −3549149.6410179655
NA / Super_Scaffold27 / −3580539.6966490704
NA / Super_Scaffold27 / −3567079.3103327616
NA / Super_Scaffold27 / −3595775.6808237229
NA / Super_Scaffold27 / −3574191.5778635913
NA / Super_Scaffold27 / −3579749.3159698341
NA / Super_Scaffold27 / −3578148.7007245170
NA / Super_Scaffold27 / −3565584.9716680041
NA / Super_Scaffold27 / −3569922.3243041509
NA / Super_Scaffold27 / −3546278.9373114998
NA / Super_Scaffold27 / −3566651.0069790818
NA / Super_Scaffold27 / −3559896.4517681859
NA / Super_Scaffold27 / −3577376.9697287618
NA / Super_Scaffold27 / −3706581.7594623067
NA / Super_Scaffold27 / −3560944.5338874143
NA / Super_Scaffold27 / −3512048.9998146086
NA / Super_Scaffold27 / −3519143.2479268014
NA / Super_Scaffold27 / −3524364.0039359736
NA / Super_Scaffold27 / −3535071.1392025868
NA / Super_Scaffold27 / −3558712.0674907118
NA / Super_Scaffold27 / −3562093.8127220958
NA / Super_Scaffold27 / −3482033.7247629929
NA / Super_Scaffold27 / −3554433.9869441874
NA / Super_Scaffold27 / −3517117.3390253303
NA / Super_Scaffold27 / −3519595.1284392467
NA / Super_Scaffold27 / −3545005.2664201758
NA / Super_Scaffold27 / −3555187.0977193434
NA / Super_Scaffold27 / −3555181.8784520957
NA / Super_Scaffold27 / −3543939.0926464424
NA / Super_Scaffold27 / −3554926.2830864727
NA / Super_Scaffold27 / −3547269.5094942157
NA / Super_Scaffold27 / −3584756.7980090841
NA / Super_Scaffold27 / −3585718.4362028218
NA / Super_Scaffold27 / −3586272.6762267491

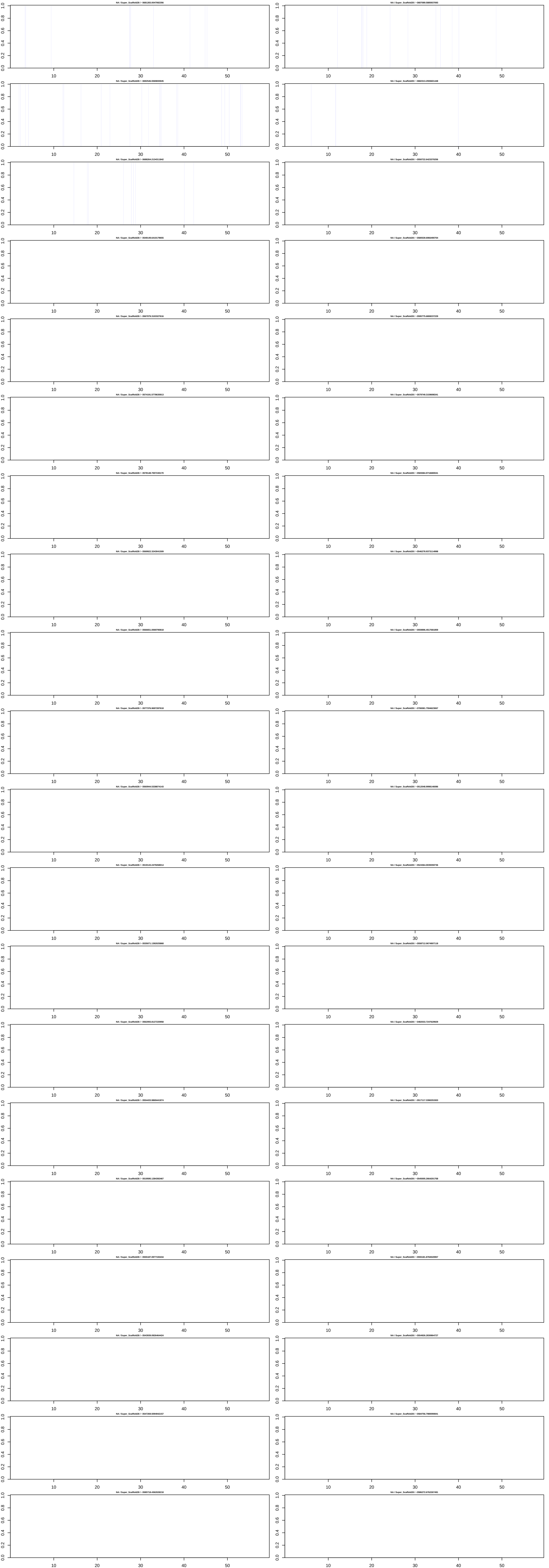

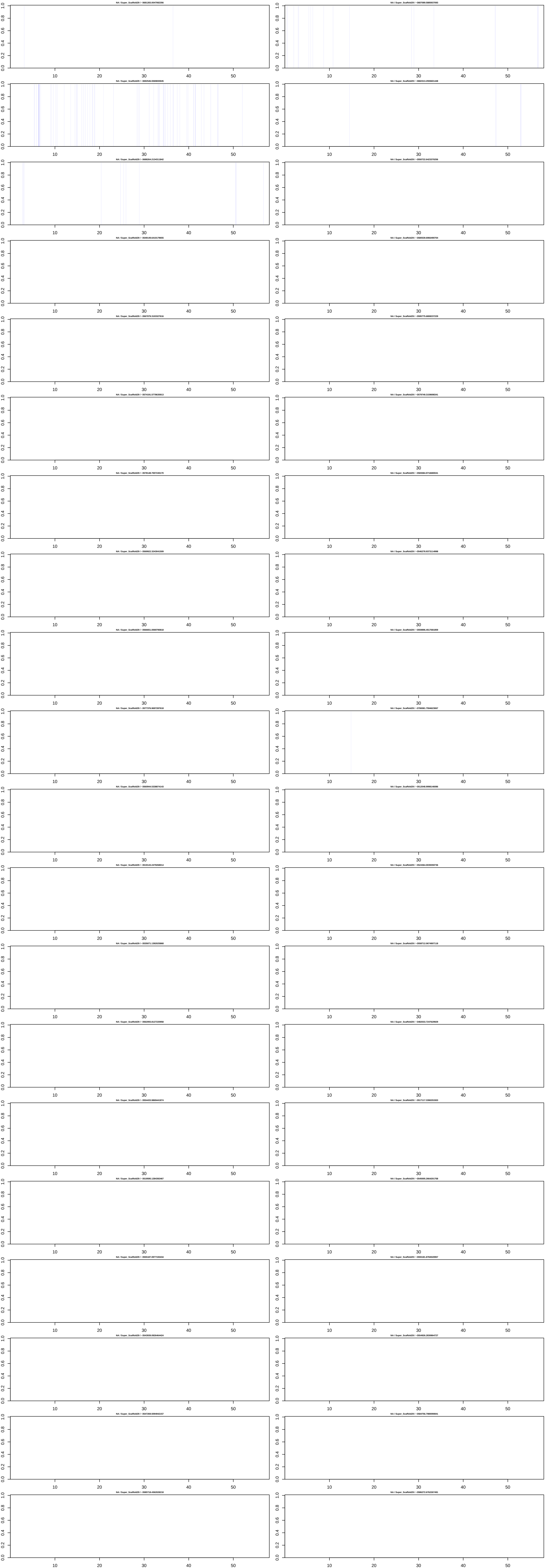

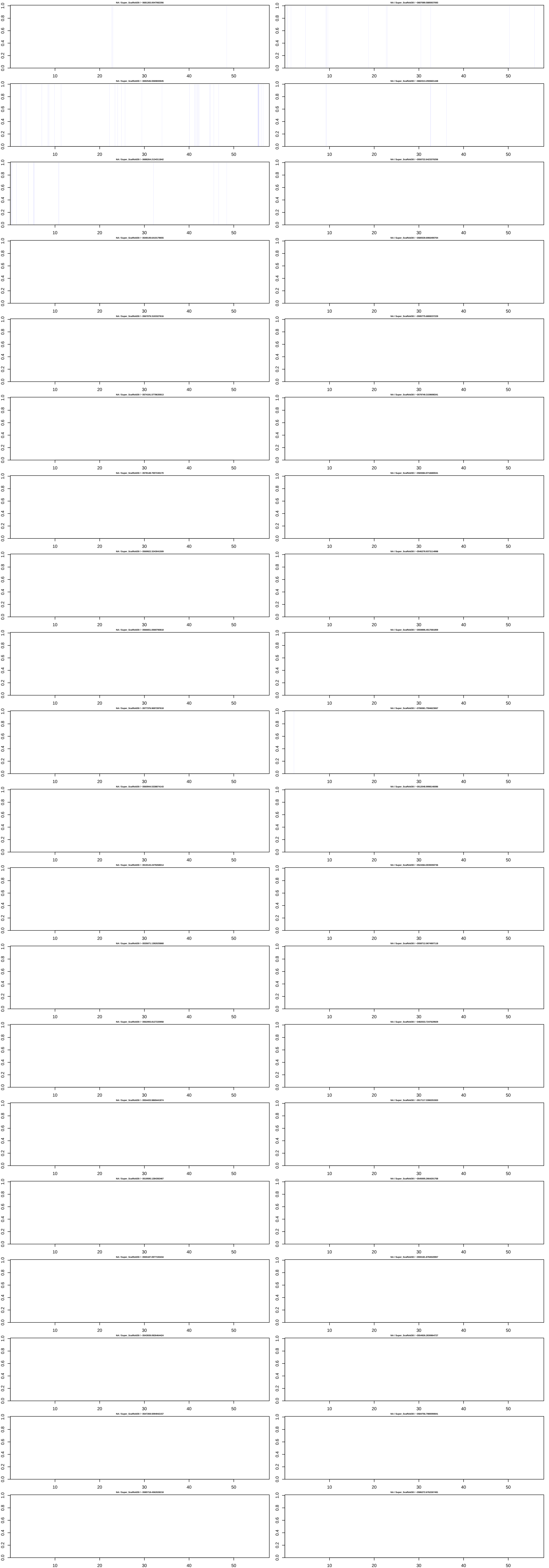

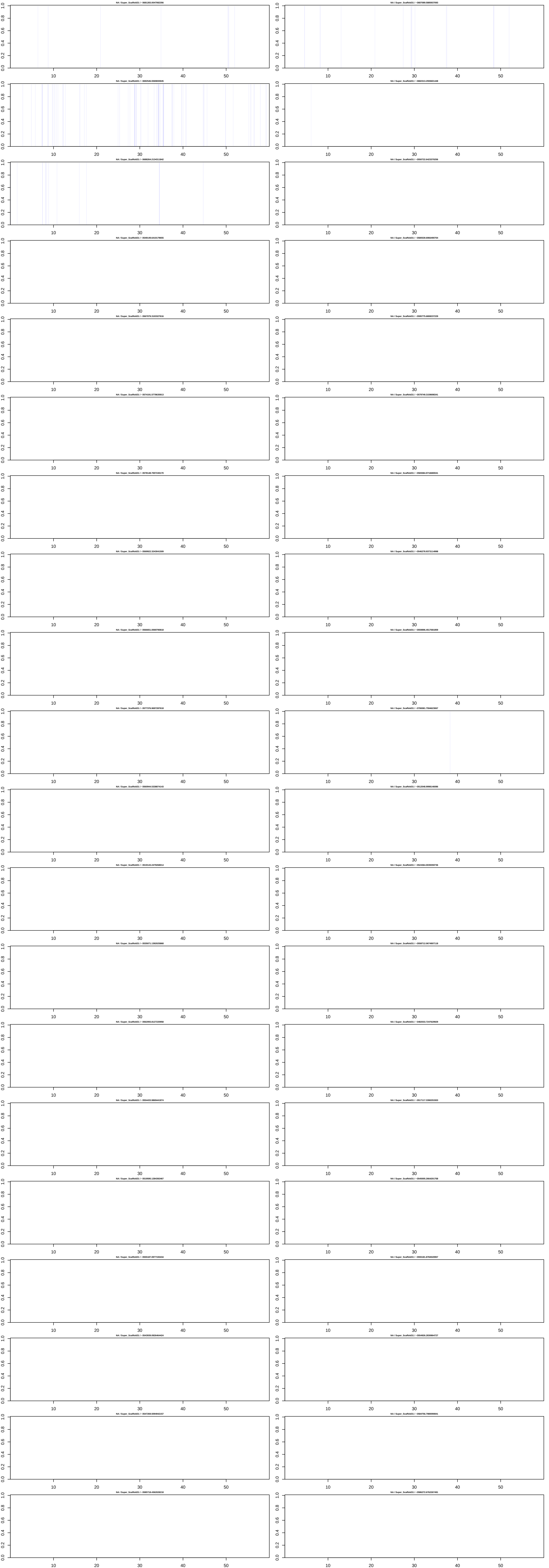

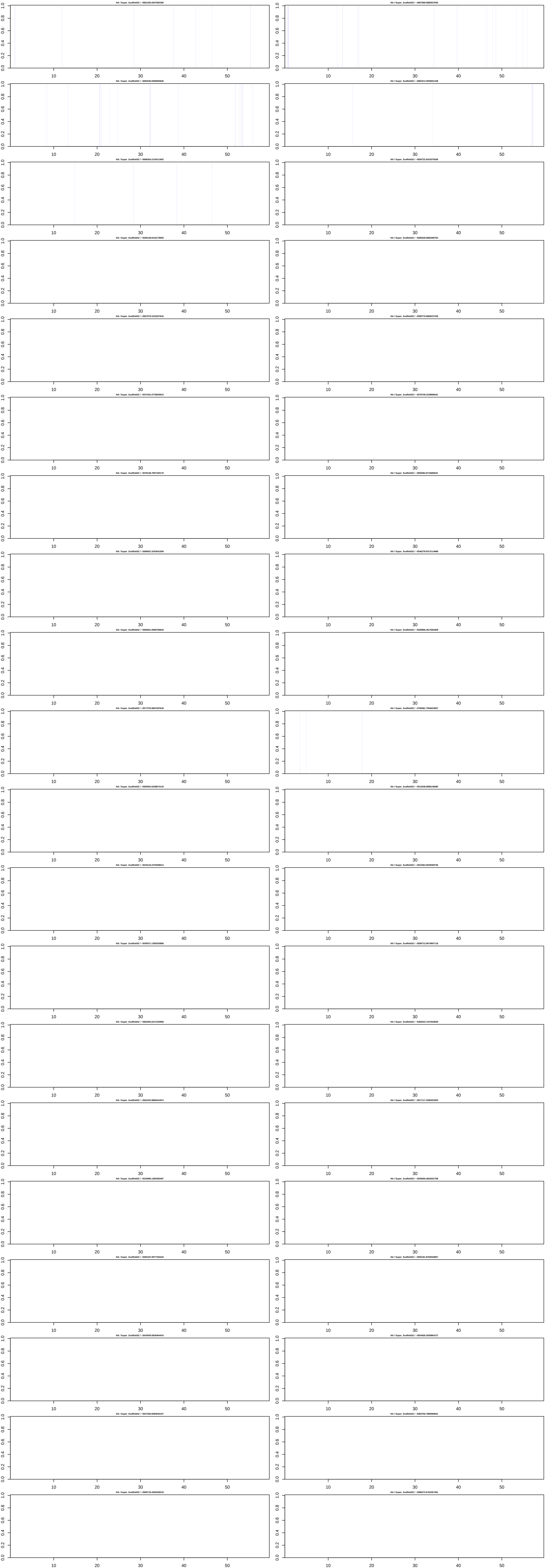

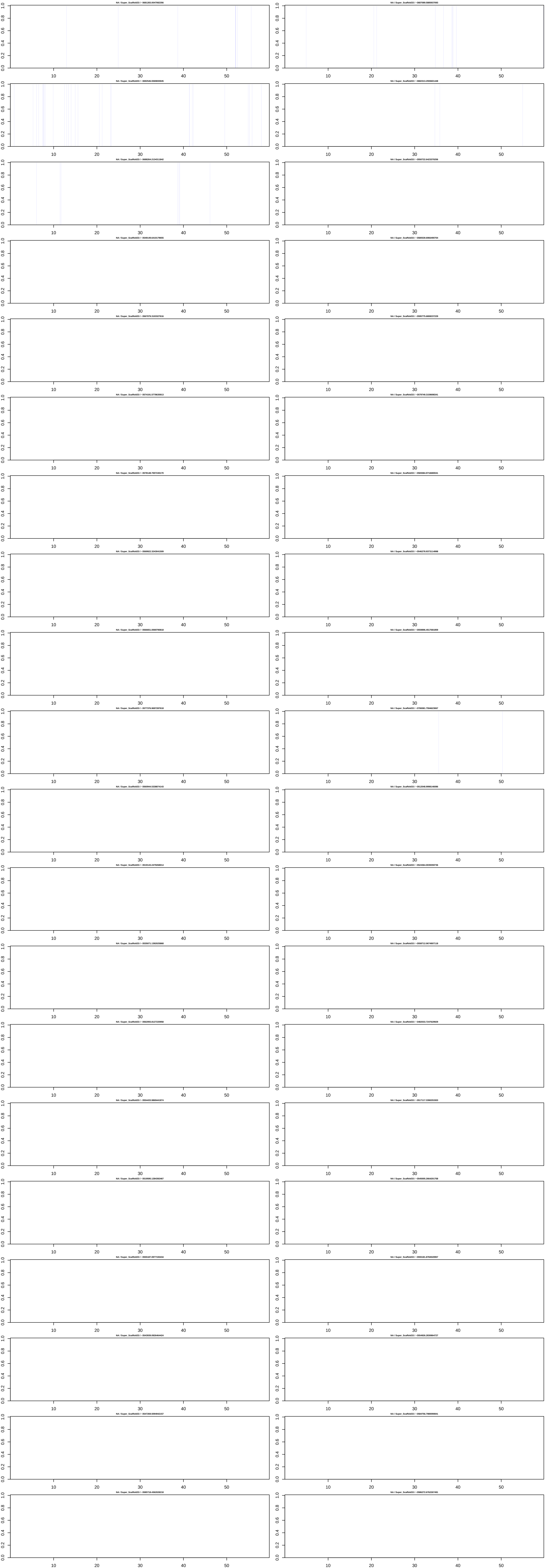

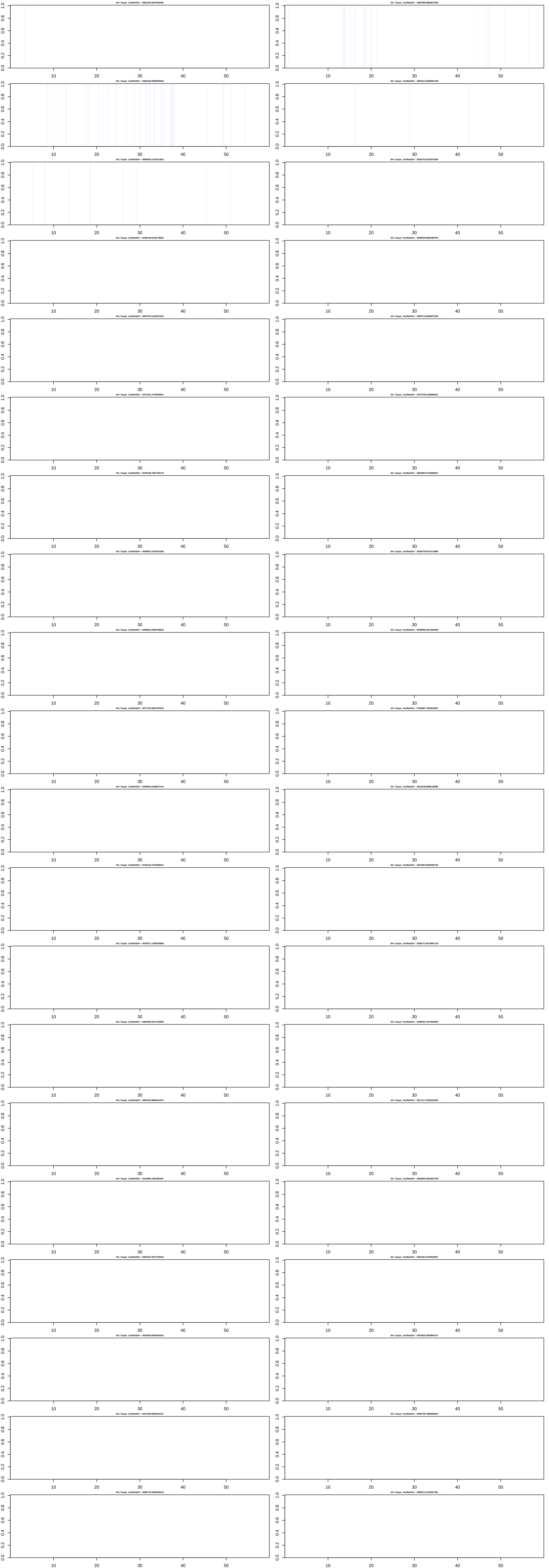
NA / Super_Scaffold34 / −3681283.0047082356
NA / Super_Scaffold34 / −3687089.5885937093
NA / Super_Scaffold34 / −3692546.9369655945
NA / Super_Scaffold34 / −3682313.2555601448
NA / Super_Scaffold34 / −3688264.2134311842
NA / Super_Scaffold34 / −3550722.6423370256
NA / Super_Scaffold34 / −3549149.6410179655
NA / Super_Scaffold34 / −3580539.6966490704
NA / Super_Scaffold34 / −3567079.3103327616
NA / Super_Scaffold34 / −3595775.6808237229
NA / Super_Scaffold34 / −3574191.5778635913
NA / Super_Scaffold34 / −3579749.3159698341
NA / Super_Scaffold34 / −3578148.7007245170
NA / Super_Scaffold34 / −3565584.9716680041
NA / Super_Scaffold34 / −3569922.3243041509
NA / Super_Scaffold34 / −3546278.9373114998
NA / Super_Scaffold34 / −3566651.0069790818
NA / Super_Scaffold34 / −3559896.4517681859
NA / Super_Scaffold34 / −3577376.9697287618
NA / Super_Scaffold34 / −3706581.7594623067
NA / Super_Scaffold34 / −3560944.5338874143
NA / Super_Scaffold34 / −3512048.9998146086
NA / Super_Scaffold34 / −3519143.2479268014
NA / Super_Scaffold34 / −3524364.0039359736
NA / Super_Scaffold34 / −3535071.1392025868
NA / Super_Scaffold34 / −3558712.0674907118
NA / Super_Scaffold34 / −3562093.8127220958
NA / Super_Scaffold34 / −3482033.7247629929
NA / Super_Scaffold34 / −3554433.9869441874
NA / Super_Scaffold34 / −3517117.3390253303
NA / Super_Scaffold34 / −3519595.1284392467
NA / Super_Scaffold34 / −3545005.2664201758
NA / Super_Scaffold34 / −3555187.0977193434
NA / Super_Scaffold34 / −3555181.8784520957
NA / Super_Scaffold34 / −3543939.0926464424
NA / Super_Scaffold34 / −3554926.2830864727
NA / Super_Scaffold34 / −3547269.5094942157
NA / Super_Scaffold34 / −3584756.7980090841
NA / Super_Scaffold34 / −3585718.4362028218
NA / Super_Scaffold34 / −3586272.6762267491

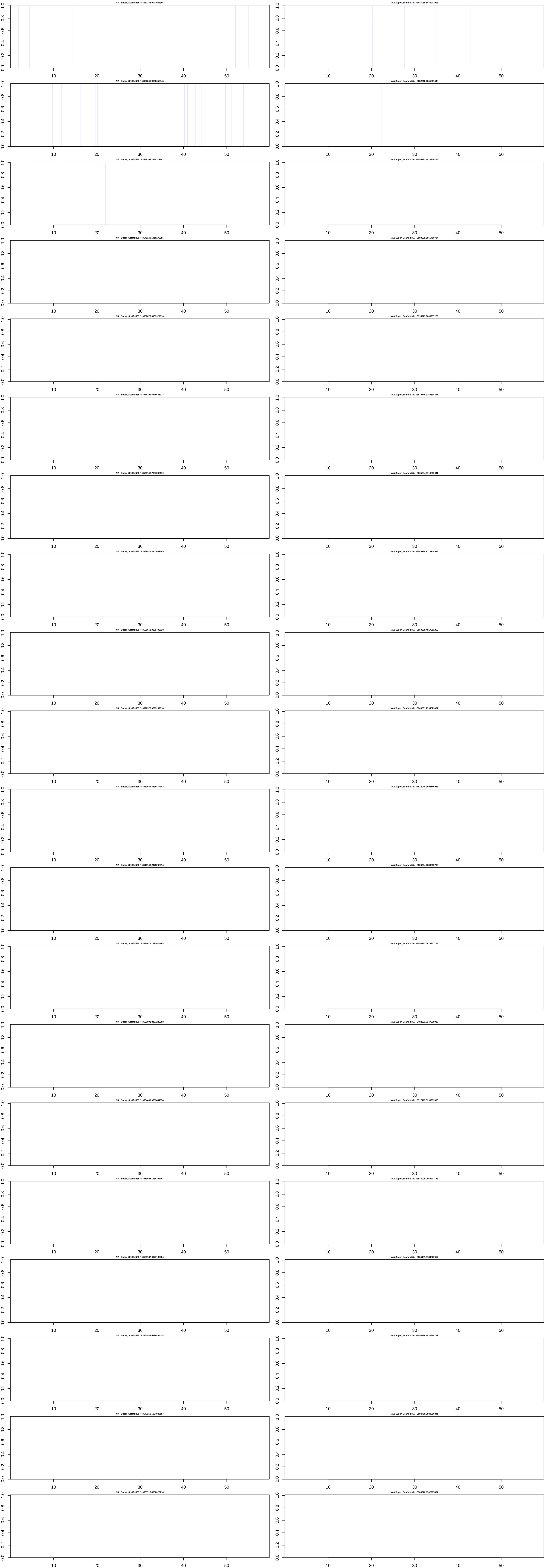

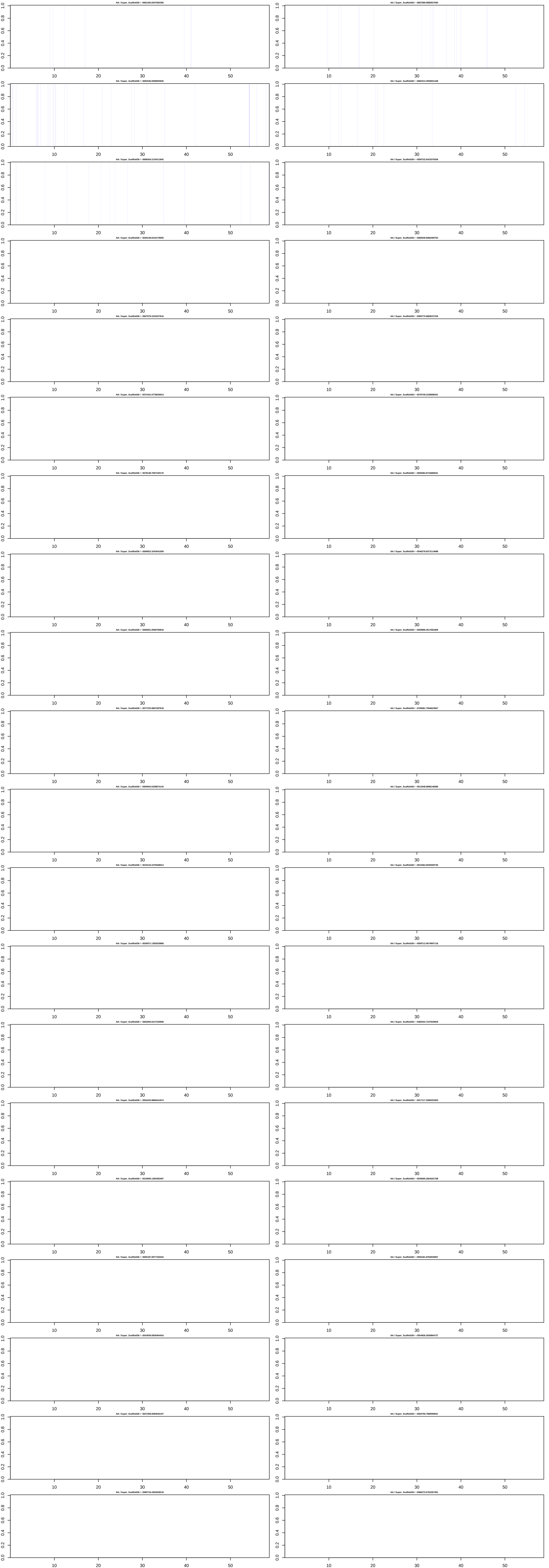

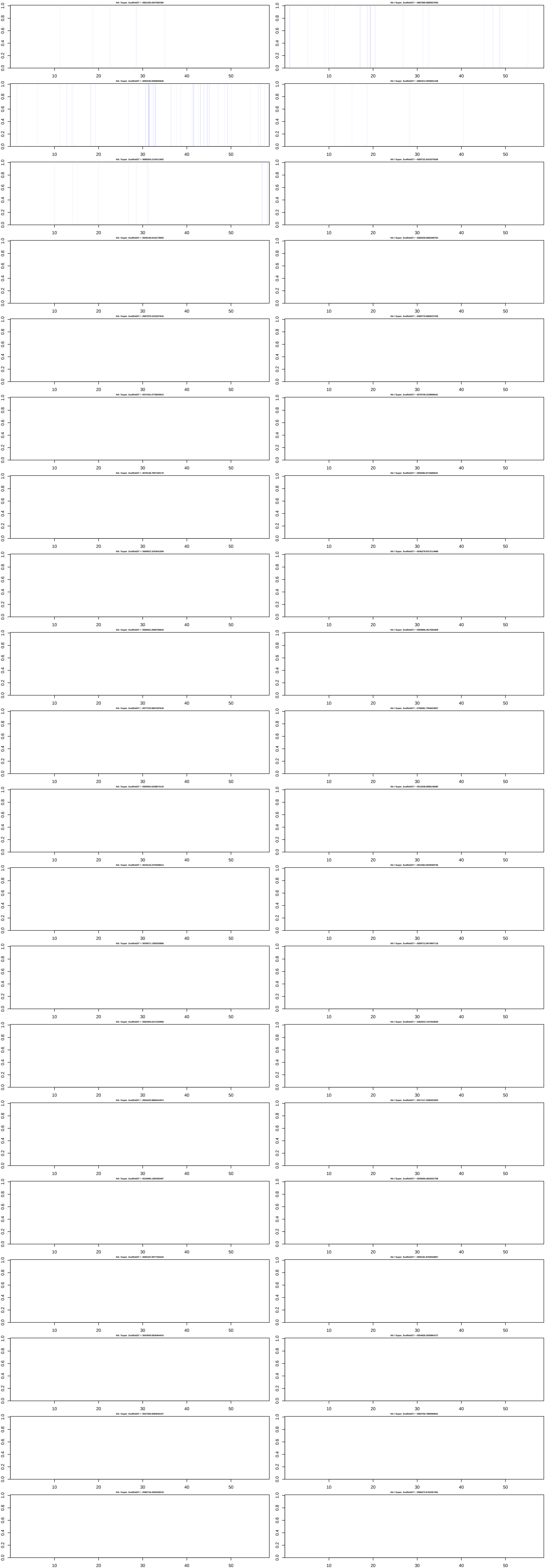

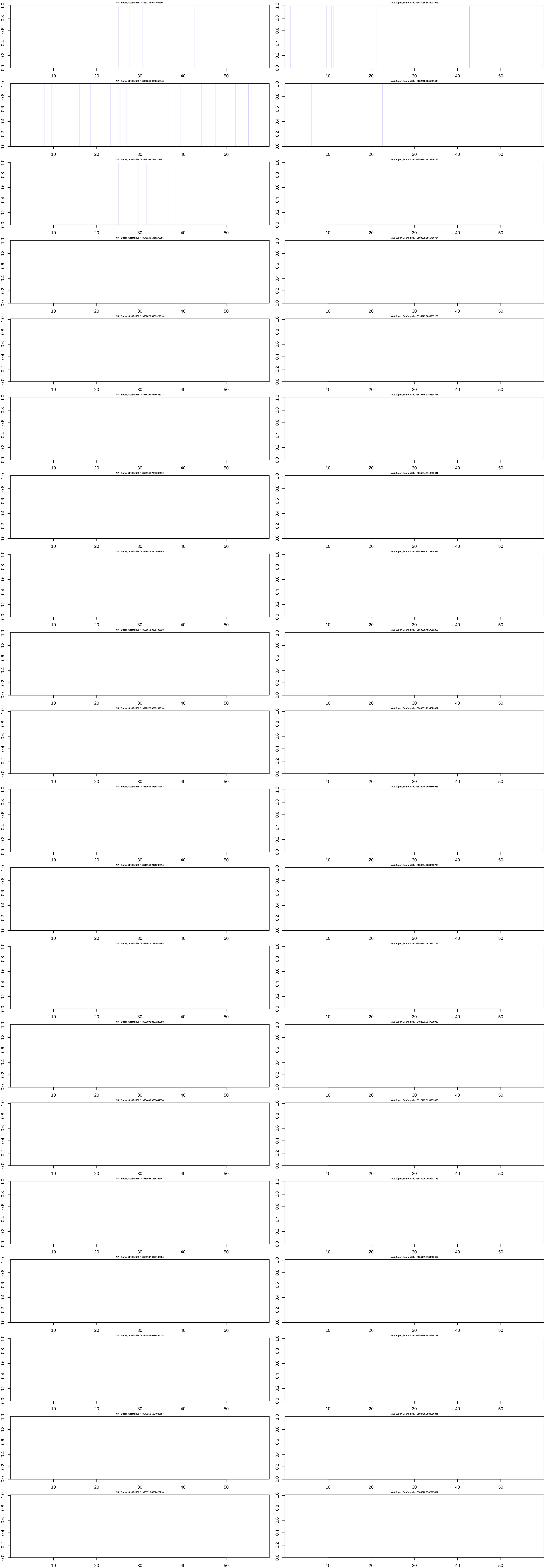

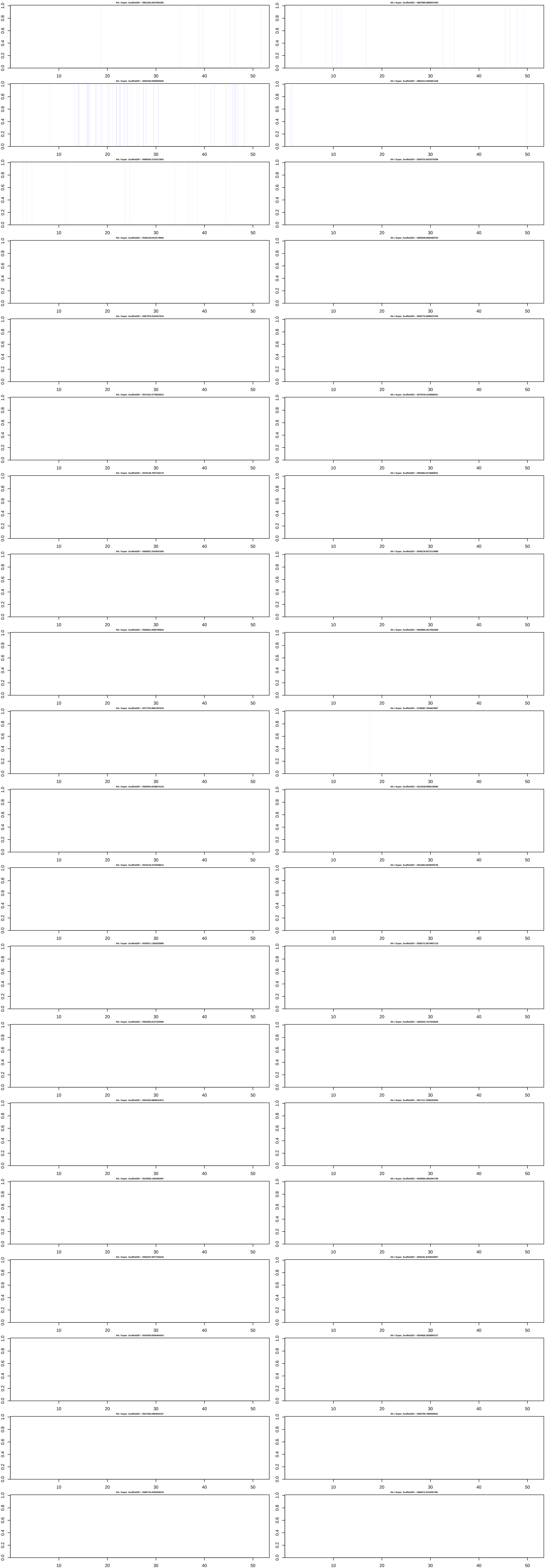

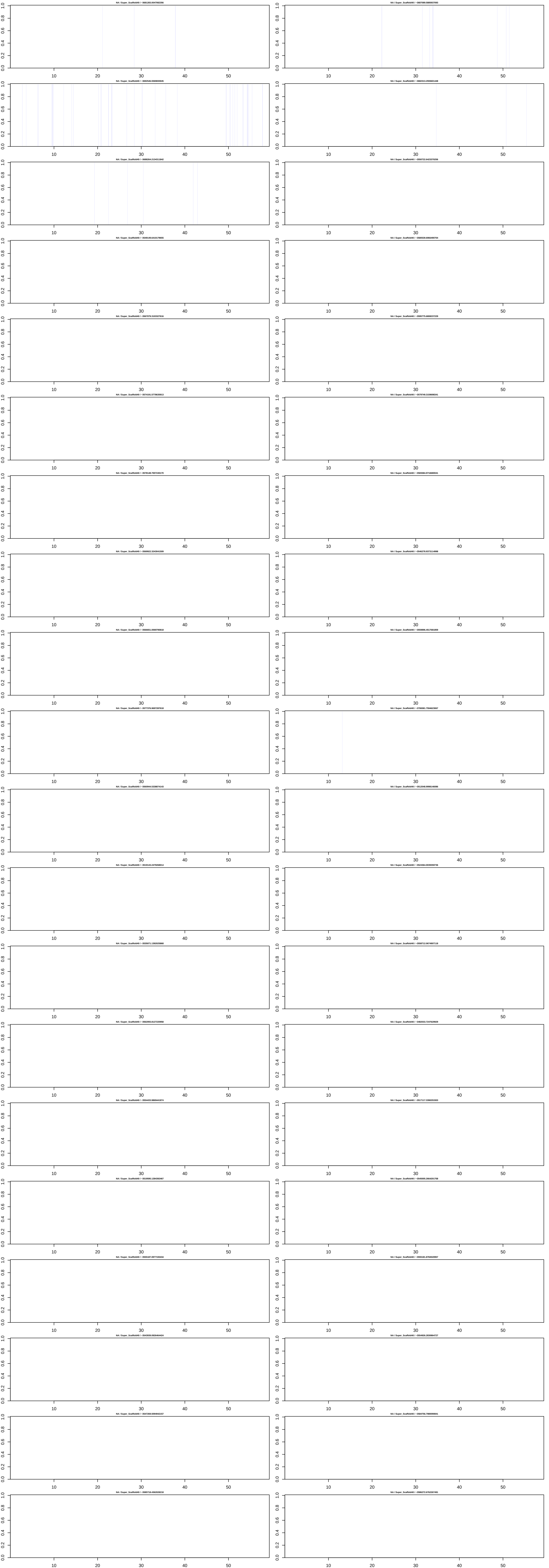

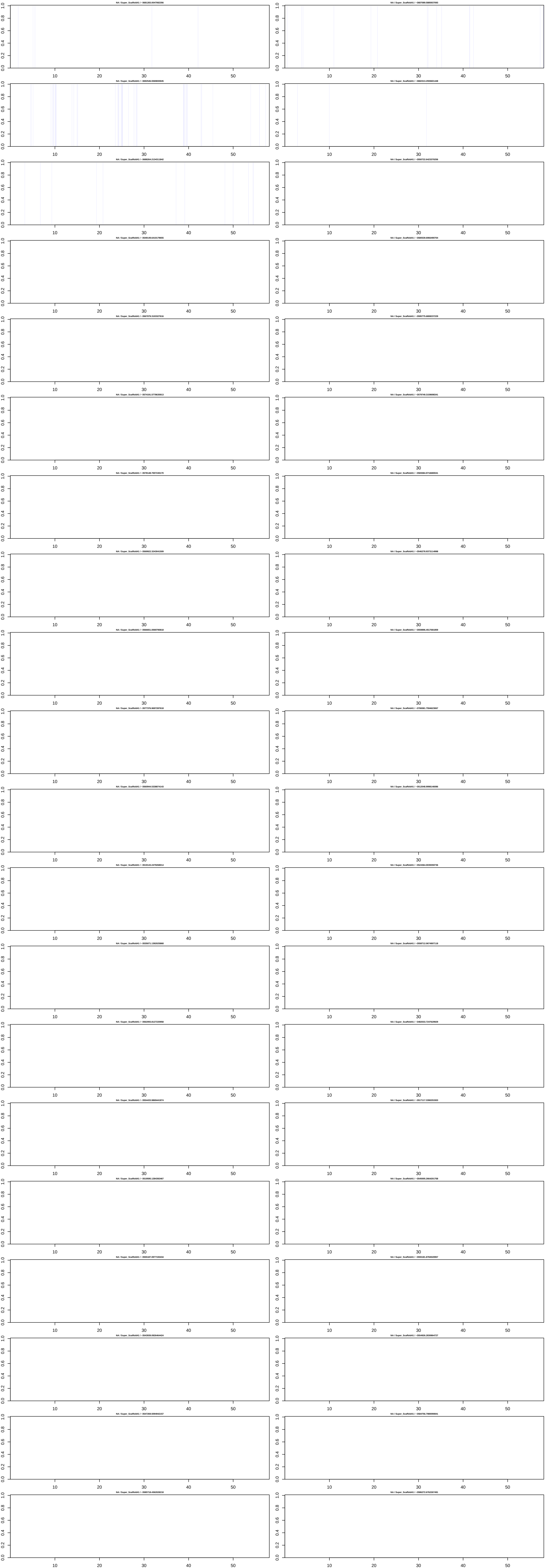
NA / Super_Scaffold41 / −3681283.0047082356
NA / Super_Scaffold41 / −3687089.5885937093
NA / Super_Scaffold41 / −3692546.9369655945
NA / Super_Scaffold41 / −3682313.2555601448
NA / Super_Scaffold41 / −3688264.2134311842
NA / Super_Scaffold41 / −3550722.6423370256
NA / Super_Scaffold41 / −3549149.6410179655
NA / Super_Scaffold41 / −3580539.6966490704
NA / Super_Scaffold41 / −3567079.3103327616
NA / Super_Scaffold41 / −3595775.6808237229
NA / Super_Scaffold41 / −3574191.5778635913
NA / Super_Scaffold41 / −3579749.3159698341
NA / Super_Scaffold41 / −3578148.7007245170
NA / Super_Scaffold41 / −3565584.9716680041
NA / Super_Scaffold41 / −3569922.3243041509
NA / Super_Scaffold41 / −3546278.9373114998
NA / Super_Scaffold41 / −3566651.0069790818
NA / Super_Scaffold41 / −3559896.4517681859
NA / Super_Scaffold41 / −3577376.9697287618
NA / Super_Scaffold41 / −3706581.7594623067
NA / Super_Scaffold41 / −3560944.5338874143
NA / Super_Scaffold41 / −3512048.9998146086
NA / Super_Scaffold41 / −3519143.2479268014
NA / Super_Scaffold41 / −3524364.0039359736
NA / Super_Scaffold41 / −3535071.1392025868
NA / Super_Scaffold41 / −3558712.0674907118
NA / Super_Scaffold41 / −3562093.8127220958
NA / Super_Scaffold41 / −3482033.7247629929
NA / Super_Scaffold41 / −3554433.9869441874
NA / Super_Scaffold41 / −3517117.3390253303
NA / Super_Scaffold41 / −3519595.1284392467
NA / Super_Scaffold41 / −3545005.2664201758
NA / Super_Scaffold41 / −3555187.0977193434
NA / Super_Scaffold41 / −3555181.8784520957
NA / Super_Scaffold41 / −3543939.0926464424
NA / Super_Scaffold41 / −3554926.2830864727
NA / Super_Scaffold41 / −3547269.5094942157
NA / Super_Scaffold41 / −3584756.7980090841
NA / Super_Scaffold41 / −3585718.4362028218
NA / Super_Scaffold41 / −3586272.6762267491

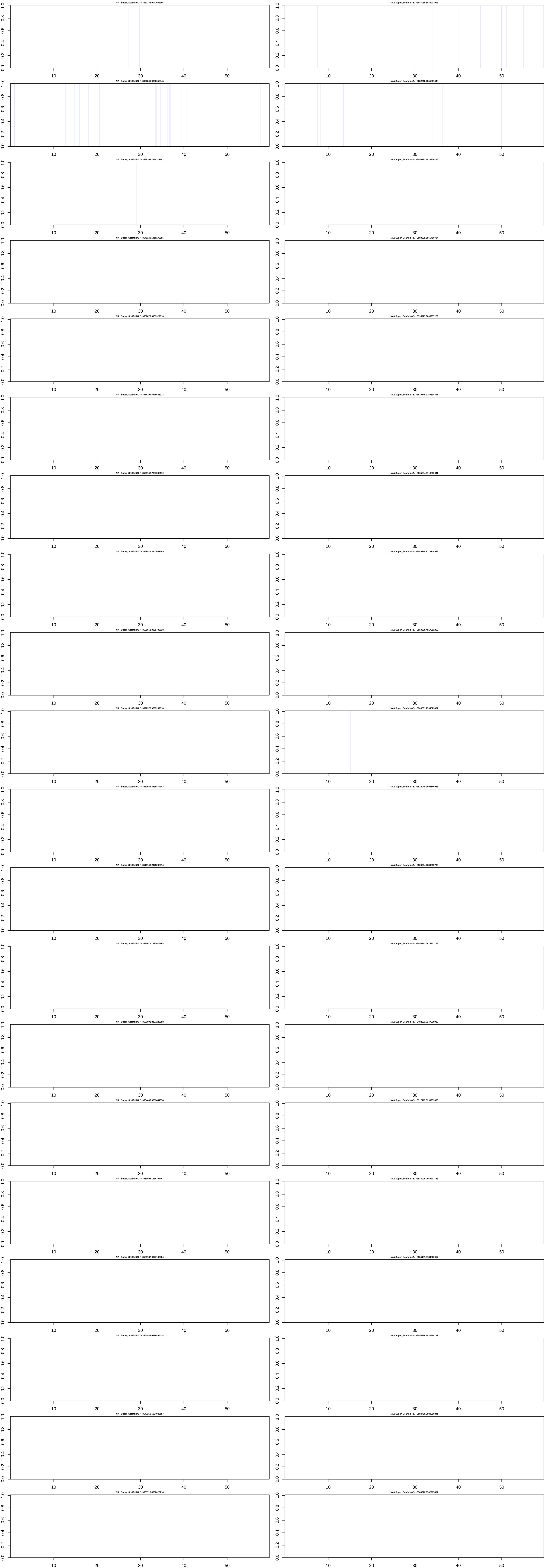

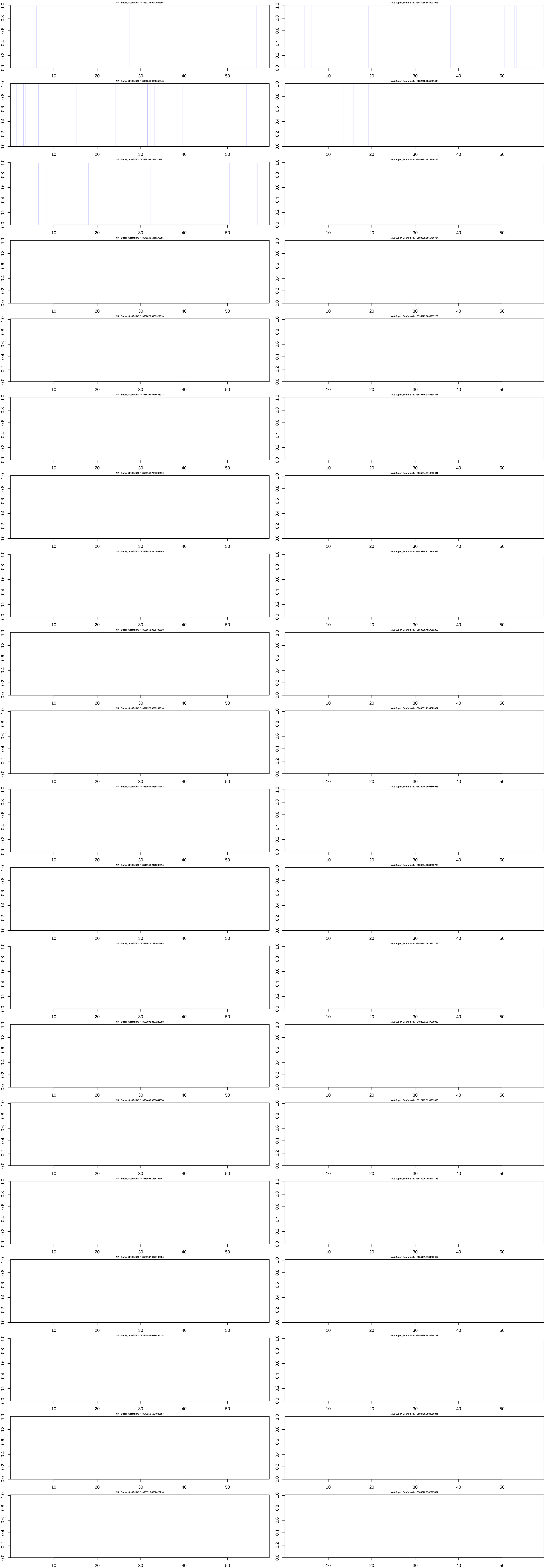

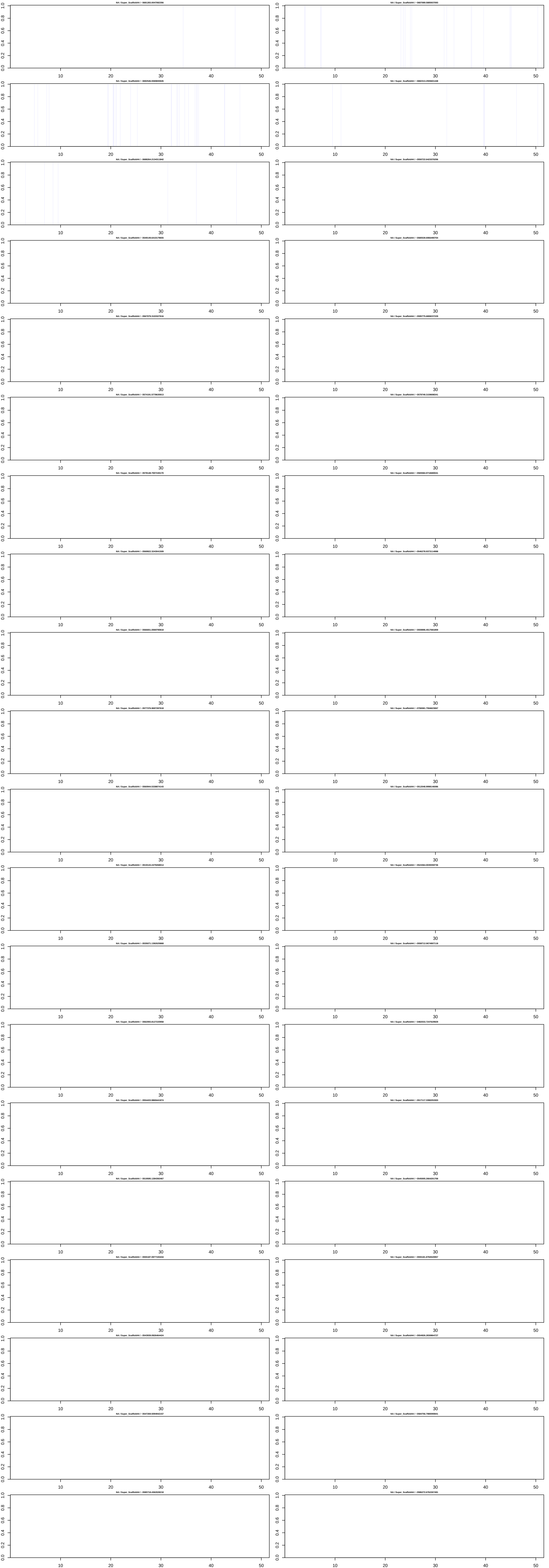

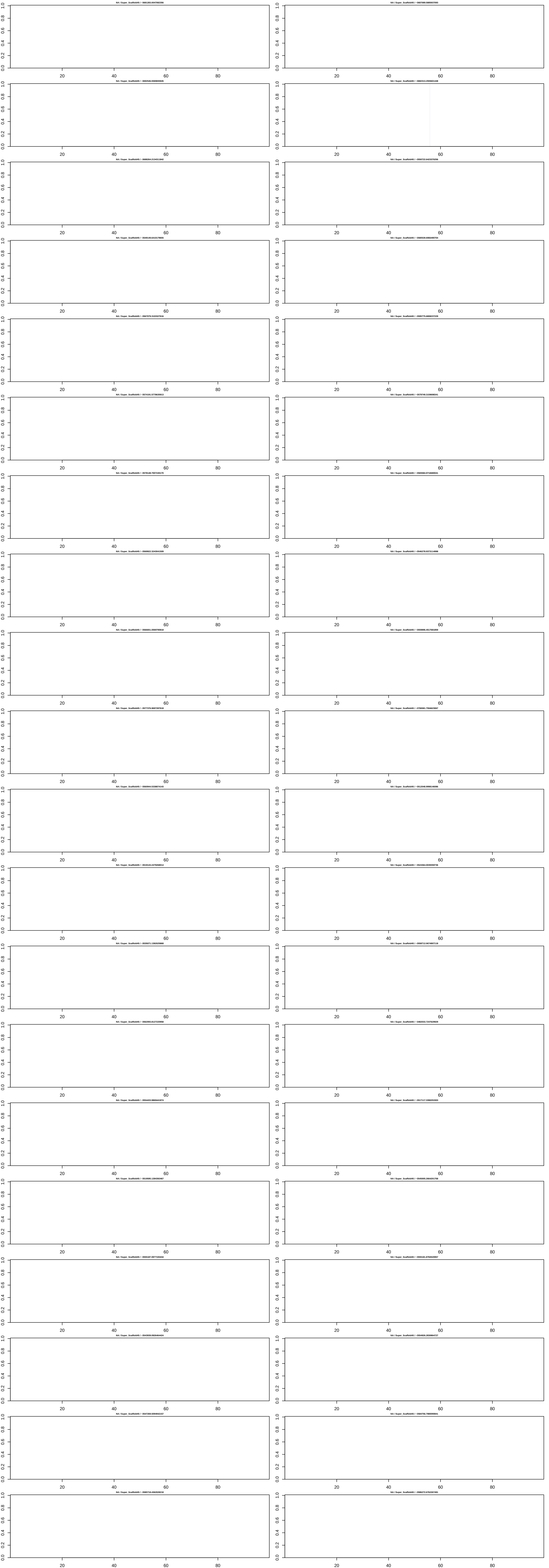

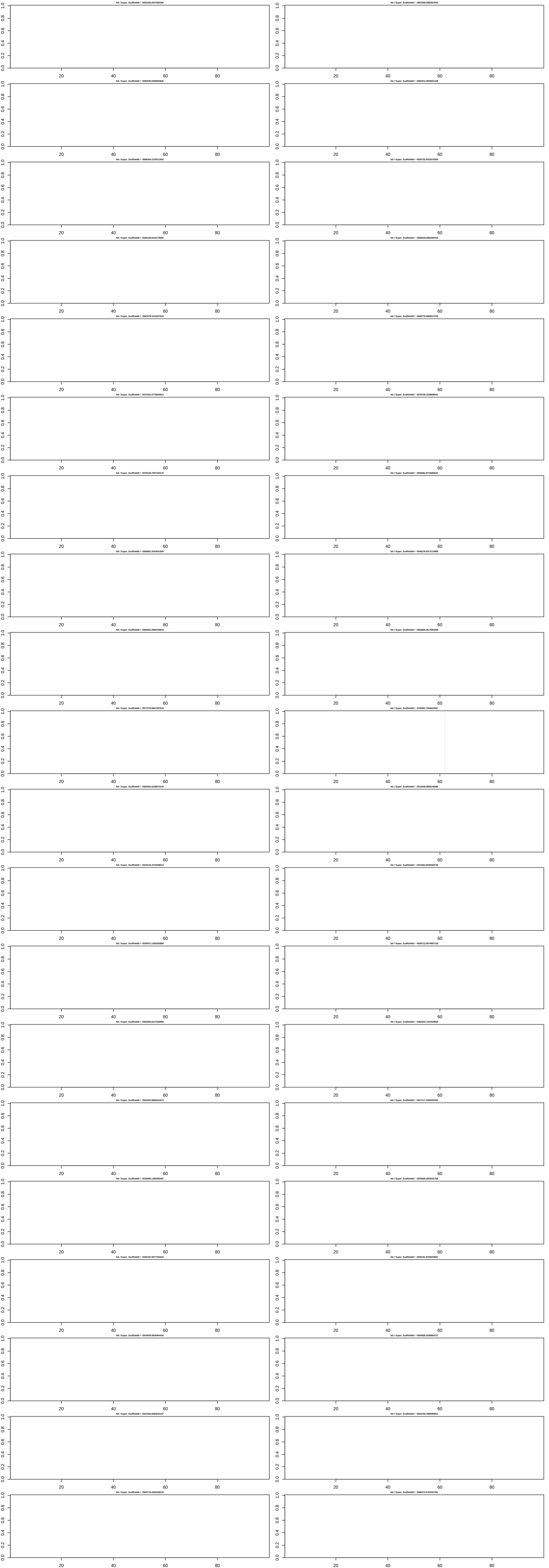
NA / Super_Scaffold46 / −3681283.0047082356
NA / Super_Scaffold46 / −3687089.5885937093
NA / Super_Scaffold46 / −3692546.9369655945
NA / Super_Scaffold46 / −3682313.2555601448
NA / Super_Scaffold46 / −3688264.2134311842
NA / Super_Scaffold46 / −3550722.6423370256
NA / Super_Scaffold46 / −3549149.6410179655
NA / Super_Scaffold46 / −3580539.6966490704
NA / Super_Scaffold46 / −3567079.3103327616
NA / Super_Scaffold46 / −3595775.6808237229
NA / Super_Scaffold46 / −3574191.5778635913
NA / Super_Scaffold46 / −3579749.3159698341
NA / Super_Scaffold46 / −3578148.7007245170
NA / Super_Scaffold46 / −3565584.9716680041
NA / Super_Scaffold46 / −3569922.3243041509
NA / Super_Scaffold46 / −3546278.9373114998
NA / Super_Scaffold46 / −3566651.0069790818
NA / Super_Scaffold46 / −3559896.4517681859
NA / Super_Scaffold46 / −3577376.9697287618
NA / Super_Scaffold46 / −3706581.7594623067
NA / Super_Scaffold46 / −3560944.5338874143
NA / Super_Scaffold46 / −3512048.9998146086
NA / Super_Scaffold46 / −3519143.2479268014
NA / Super_Scaffold46 / −3524364.0039359736
NA / Super_Scaffold46 / −3535071.1392025868
NA / Super_Scaffold46 / −3558712.0674907118
NA / Super_Scaffold46 / −3562093.8127220958
NA / Super_Scaffold46 / −3482033.7247629929
NA / Super_Scaffold46 / −3554433.9869441874
NA / Super_Scaffold46 / −3517117.3390253303
NA / Super_Scaffold46 / −3519595.1284392467
NA / Super_Scaffold46 / −3545005.2664201758
NA / Super_Scaffold46 / −3555187.0977193434
NA / Super_Scaffold46 / −3555181.8784520957
NA / Super_Scaffold46 / −3543939.0926464424
NA / Super_Scaffold46 / −3554926.2830864727
NA / Super_Scaffold46 / −3547269.5094942157
NA / Super_Scaffold46 / −3584756.7980090841
NA / Super_Scaffold46 / −3585718.4362028218
NA / Super_Scaffold46 / −3586272.6762267491

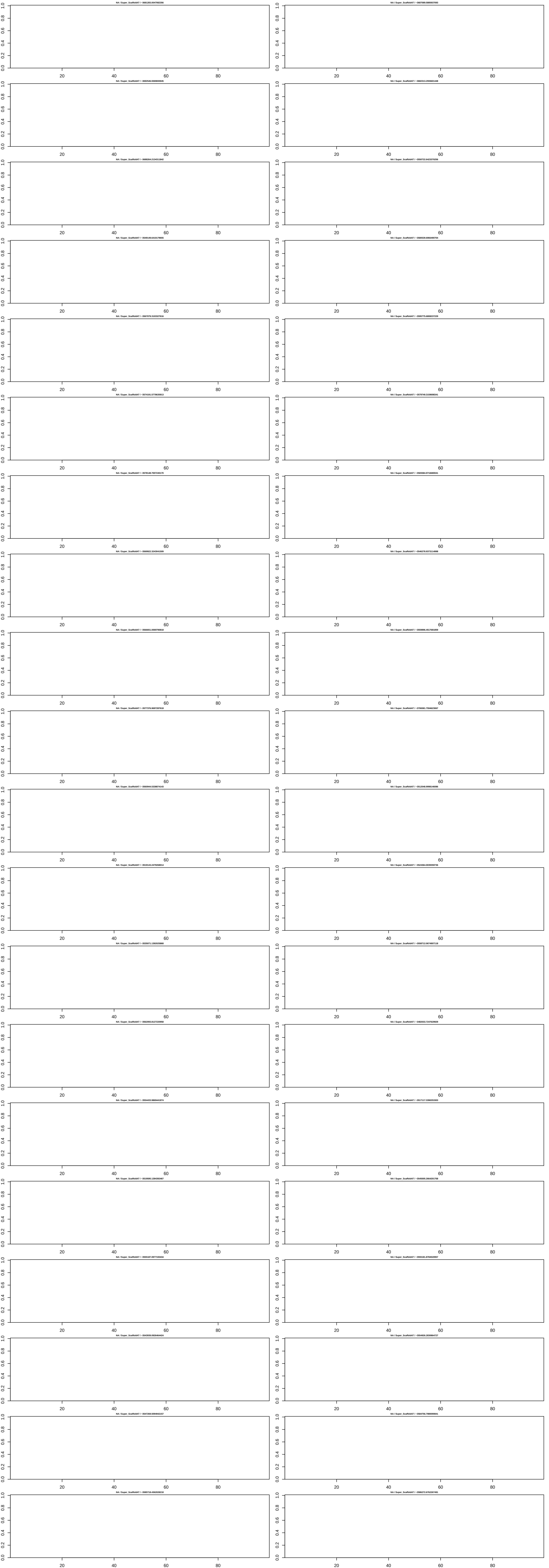
NA / Super_Scaffold47 / −3681283.0047082356
NA / Super_Scaffold47 / −3687089.5885937093
NA / Super_Scaffold47 / −3692546.9369655945
NA / Super_Scaffold47 / −3682313.2555601448
NA / Super_Scaffold47 / −3688264.2134311842
NA / Super_Scaffold47 / −3550722.6423370256
NA / Super_Scaffold47 / −3549149.6410179655
NA / Super_Scaffold47 / −3580539.6966490704
NA / Super_Scaffold47 / −3567079.3103327616
NA / Super_Scaffold47 / −3595775.6808237229
NA / Super_Scaffold47 / −3574191.5778635913
NA / Super_Scaffold47 / −3579749.3159698341
NA / Super_Scaffold47 / −3578148.7007245170
NA / Super_Scaffold47 / −3565584.9716680041
NA / Super_Scaffold47 / −3569922.3243041509
NA / Super_Scaffold47 / −3546278.9373114998
NA / Super_Scaffold47 / −3566651.0069790818
NA / Super_Scaffold47 / −3559896.4517681859
NA / Super_Scaffold47 / −3577376.9697287618
NA / Super_Scaffold47 / −3706581.7594623067
NA / Super_Scaffold47 / −3560944.5338874143
NA / Super_Scaffold47 / −3512048.9998146086
NA / Super_Scaffold47 / −3519143.2479268014
NA / Super_Scaffold47 / −3524364.0039359736
NA / Super_Scaffold47 / −3535071.1392025868
NA / Super_Scaffold47 / −3558712.0674907118
NA / Super_Scaffold47 / −3562093.8127220958
NA / Super_Scaffold47 / −3482033.7247629929
NA / Super_Scaffold47 / −3554433.9869441874
NA / Super_Scaffold47 / −3517117.3390253303
NA / Super_Scaffold47 / −3519595.1284392467
NA / Super_Scaffold47 / −3545005.2664201758
NA / Super_Scaffold47 / −3555187.0977193434
NA / Super_Scaffold47 / −3555181.8784520957
NA / Super_Scaffold47 / −3543939.0926464424
NA / Super_Scaffold47 / −3554926.2830864727
NA / Super_Scaffold47 / −3547269.5094942157
NA / Super_Scaffold47 / −3584756.7980090841
NA / Super_Scaffold47 / −3585718.4362028218
NA / Super_Scaffold47 / −3586272.6762267491

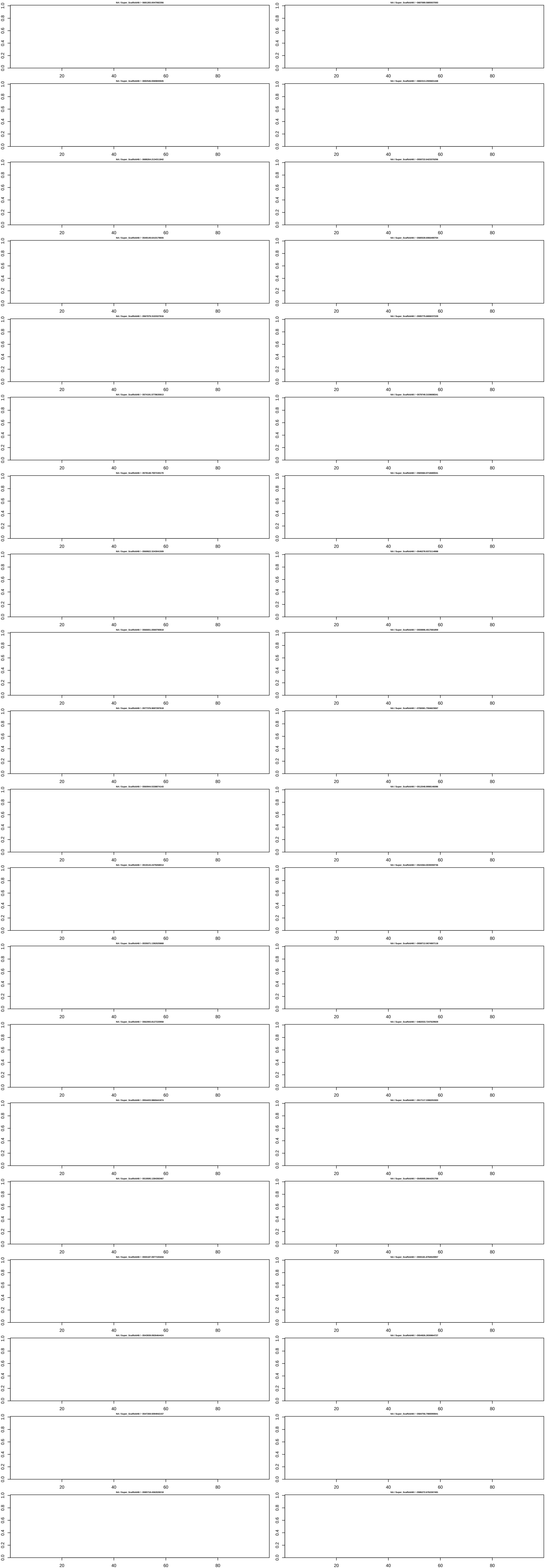
NA / Super_Scaffold48 / −3681283.0047082356
NA / Super_Scaffold48 / −3687089.5885937093
NA / Super_Scaffold48 / −3692546.9369655945
NA / Super_Scaffold48 / −3682313.2555601448
NA / Super_Scaffold48 / −3688264.2134311842
NA / Super_Scaffold48 / −3550722.6423370256
NA / Super_Scaffold48 / −3549149.6410179655
NA / Super_Scaffold48 / −3580539.6966490704
NA / Super_Scaffold48 / −3567079.3103327616
NA / Super_Scaffold48 / −3595775.6808237229
NA / Super_Scaffold48 / −3574191.5778635913
NA / Super_Scaffold48 / −3579749.3159698341
NA / Super_Scaffold48 / −3578148.7007245170
NA / Super_Scaffold48 / −3565584.9716680041
NA / Super_Scaffold48 / −3569922.3243041509
NA / Super_Scaffold48 / −3546278.9373114998
NA / Super_Scaffold48 / −3566651.0069790818
NA / Super_Scaffold48 / −3559896.4517681859
NA / Super_Scaffold48 / −3577376.9697287618
NA / Super_Scaffold48 / −3706581.7594623067
NA / Super_Scaffold48 / −3560944.5338874143
NA / Super_Scaffold48 / −3512048.9998146086
NA / Super_Scaffold48 / −3519143.2479268014
NA / Super_Scaffold48 / −3524364.0039359736
NA / Super_Scaffold48 / −3535071.1392025868
NA / Super_Scaffold48 / −3558712.0674907118
NA / Super_Scaffold48 / −3562093.8127220958
NA / Super_Scaffold48 / −3482033.7247629929
NA / Super_Scaffold48 / −3554433.9869441874
NA / Super_Scaffold48 / −3517117.3390253303
NA / Super_Scaffold48 / −3519595.1284392467
NA / Super_Scaffold48 / −3545005.2664201758
NA / Super_Scaffold48 / −3555187.0977193434
NA / Super_Scaffold48 / −3555181.8784520957
NA / Super_Scaffold48 / −3543939.0926464424
NA / Super_Scaffold48 / −3554926.2830864727
NA / Super_Scaffold48 / −3547269.5094942157
NA / Super_Scaffold48 / −3584756.7980090841
NA / Super_Scaffold48 / −3585718.4362028218
NA / Super_Scaffold48 / −3586272.6762267491

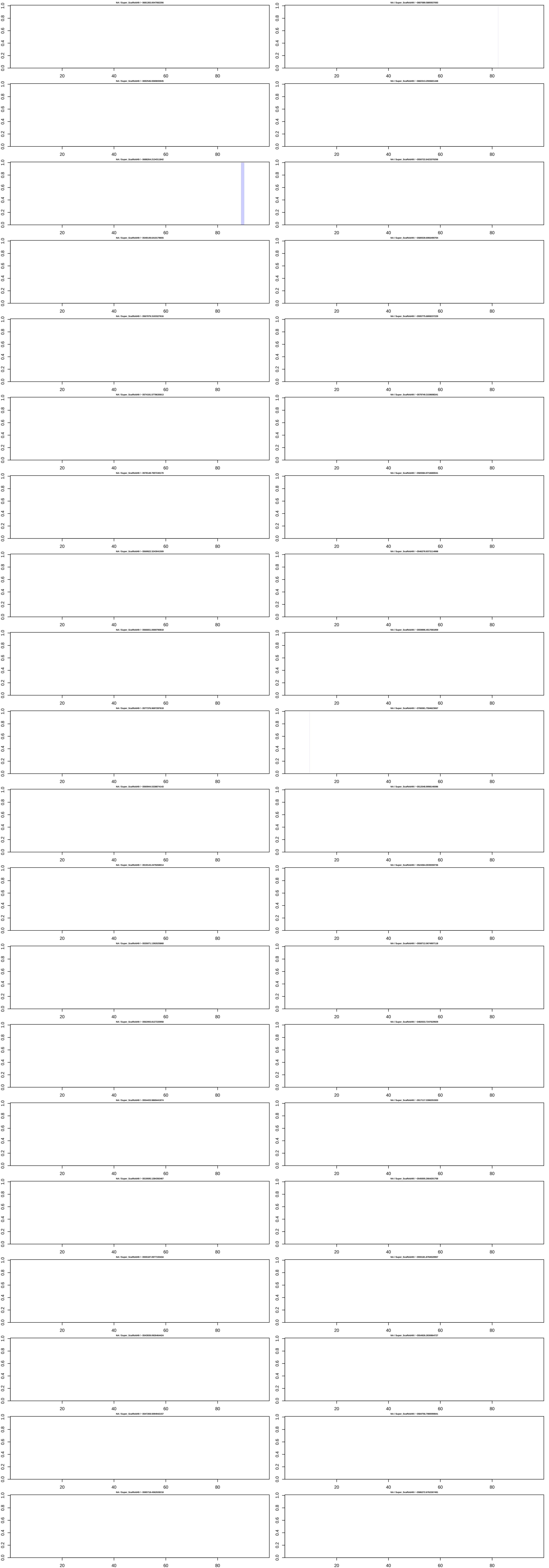

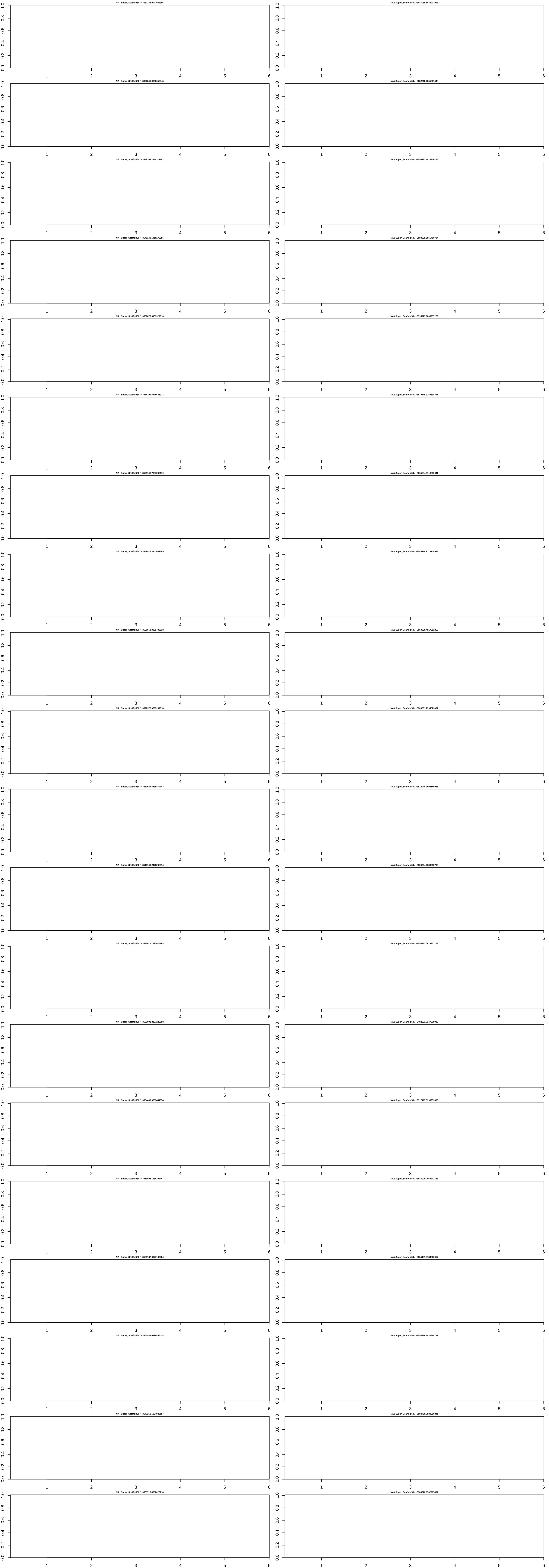
NA / Super_Scaffold50 / −3681283.0047082356
NA / Super_Scaffold50 / −3687089.5885937093
NA / Super_Scaffold50 / −3692546.9369655945
NA / Super_Scaffold50 / −3682313.2555601448
NA / Super_Scaffold50 / −3688264.2134311842
NA / Super_Scaffold50 / −3550722.6423370256
NA / Super_Scaffold50 / −3549149.6410179655
NA / Super_Scaffold50 / −3580539.6966490704
NA / Super_Scaffold50 / −3567079.3103327616
NA / Super_Scaffold50 / −3595775.6808237229
NA / Super_Scaffold50 / −3574191.5778635913
NA / Super_Scaffold50 / −3579749.3159698341
NA / Super_Scaffold50 / −3578148.7007245170
NA / Super_Scaffold50 / −3565584.9716680041
NA / Super_Scaffold50 / −3569922.3243041509
NA / Super_Scaffold50 / −3546278.9373114998
NA / Super_Scaffold50 / −3566651.0069790818
NA / Super_Scaffold50 / −3559896.4517681859
NA / Super_Scaffold50 / −3577376.9697287618
NA / Super_Scaffold50 / −3706581.7594623067
NA / Super_Scaffold50 / −3560944.5338874143
NA / Super_Scaffold50 / −3512048.9998146086
NA / Super_Scaffold50 / −3519143.2479268014
NA / Super_Scaffold50 / −3524364.0039359736
NA / Super_Scaffold50 / −3535071.1392025868
NA / Super_Scaffold50 / −3558712.0674907118
NA / Super_Scaffold50 / −3562093.8127220958
NA / Super_Scaffold50 / −3482033.7247629929
NA / Super_Scaffold50 / −3554433.9869441874
NA / Super_Scaffold50 / −3517117.3390253303
NA / Super_Scaffold50 / −3519595.1284392467
NA / Super_Scaffold50 / −3545005.2664201758
NA / Super_Scaffold50 / −3555187.0977193434
NA / Super_Scaffold50 / −3555181.8784520957
NA / Super_Scaffold50 / −3543939.0926464424
NA / Super_Scaffold50 / −3554926.2830864727
NA / Super_Scaffold50 / −3547269.5094942157
NA / Super_Scaffold50 / −3584756.7980090841
NA / Super_Scaffold50 / −3585718.4362028218
NA / Super_Scaffold50 / −3586272.6762267491

**Supplementary Fig. 3. Inbreeding tracts, or identical by descent (IBD), plots.** The plots were produced using the program ngsF-HMM for all super-scaffolds and all buffalo samples. The x-axis indicates the sites along the super-scaffold and the y-axis indicates whether the site was IBD (1) or not (0), as shown by the blue bars. Given the scale of the x-axis (~60 Mb), an extended IBD run is necessary in order for the blue bar to become visible.

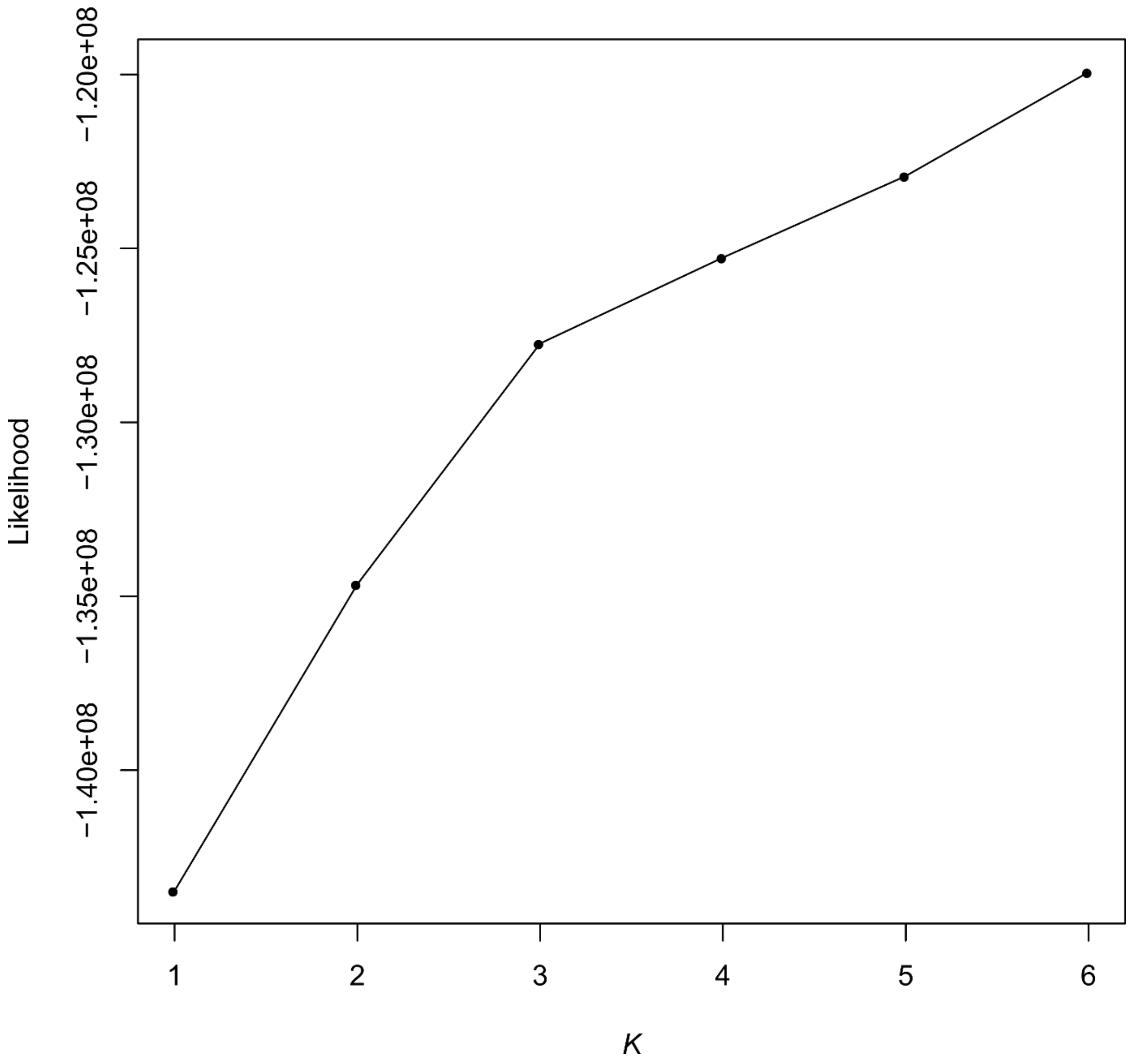


**Supplementary Fig. 4. Best likelihood for *K* = 1 to *K* = 6 of the NGSadmix analysis.**

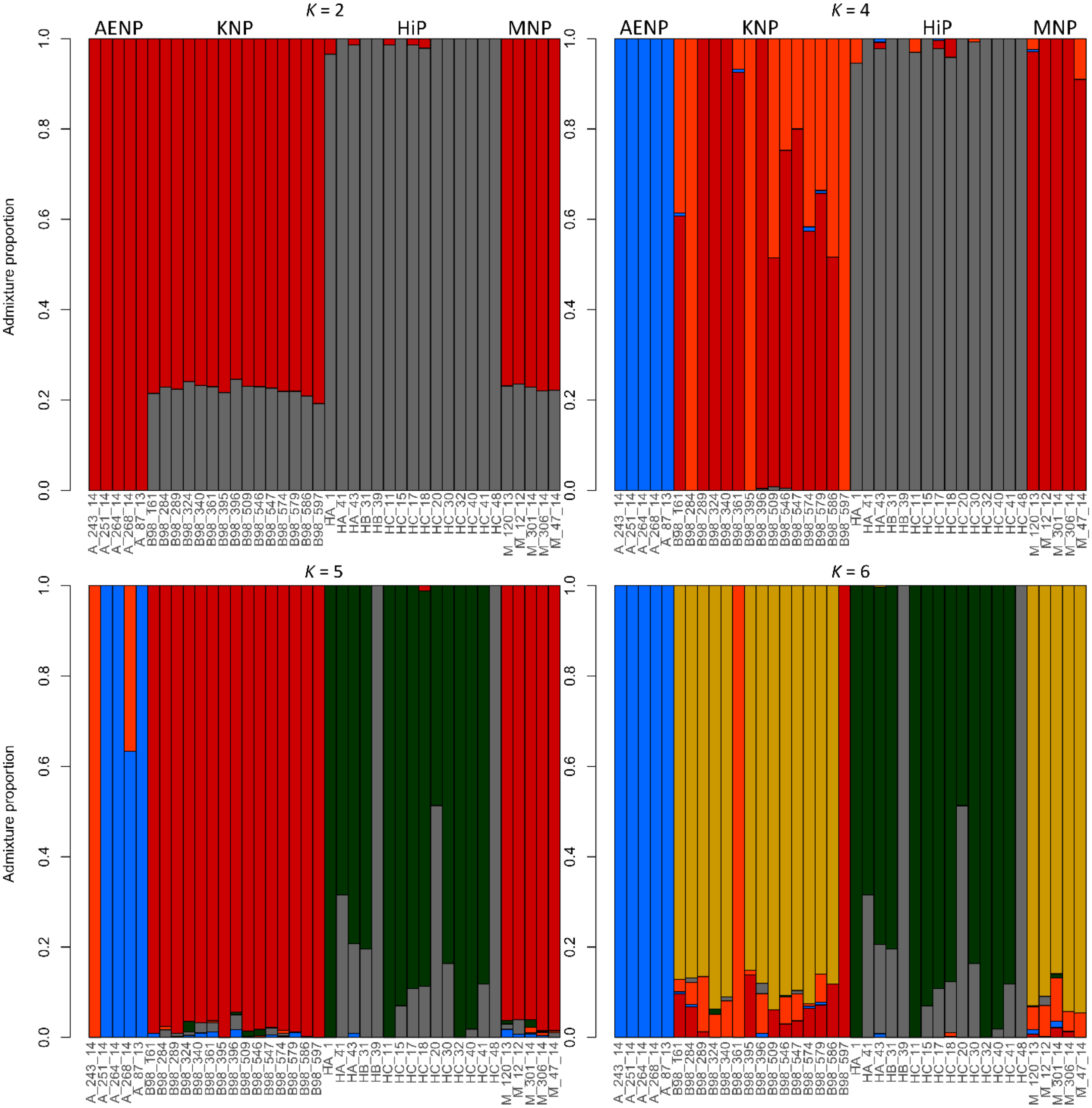


**Supplementary Fig. 5. Individual assignment plots of the NGSadmix analysis at *K* = 2, 4, 5 and 6.**

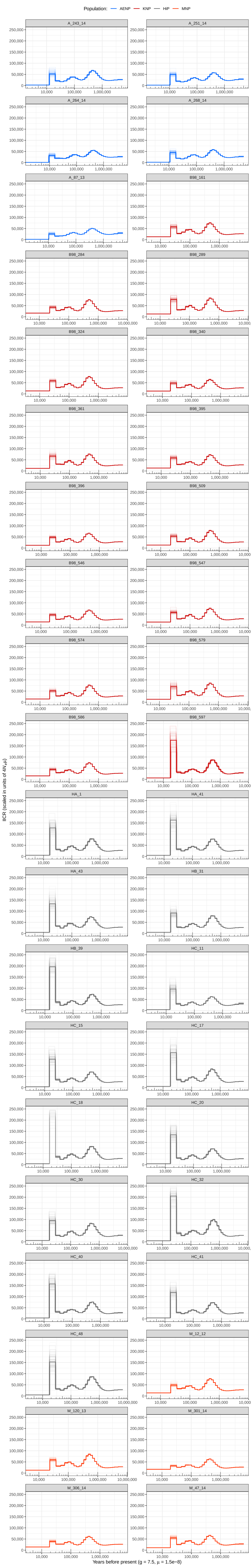

Population: AENP KNP HiP MNP
A_243_14
A_251_14
A_264_14
A_268_14
A_87_13
B98_161
B98_284
B98_289
B98_324
B98_340
B98_361
B98_395
B98_396
B98_509
B98_546
B98_547
B98_574
B98_579
B98_586
B98_597
HA_1
HA_41
HA_43
HB_31
HB_39
HC_11
HC_15
HC_17
HC_18
HC_20
HC_30
HC_32
HC_40
HC_41
HC_48
M_12_12
M_120_13
M_301_14
M_306_14
M_47_14
IICR (scaled in units of 4N0μ)
Years before present (g = 7.5, μ = 1.5e−8)

**Supplementary Fig. 6. Pairwise sequentially Markovian coalescent (PSMC) inference of the inverse instantaneous coalescent rate (IICR) in each Cape buffalo sample, plotted by sampling locality.** The x-axis represents time in years, calibrated using a generation time (g) of 7.5 years and a per site per generation mutation rate (μ) of 1.5e-8. The y-axis shows IICR, which can be interpreted as $N_e$ in an unstructured population. The faded lines show the 100 bootstrap replicates for each sample.

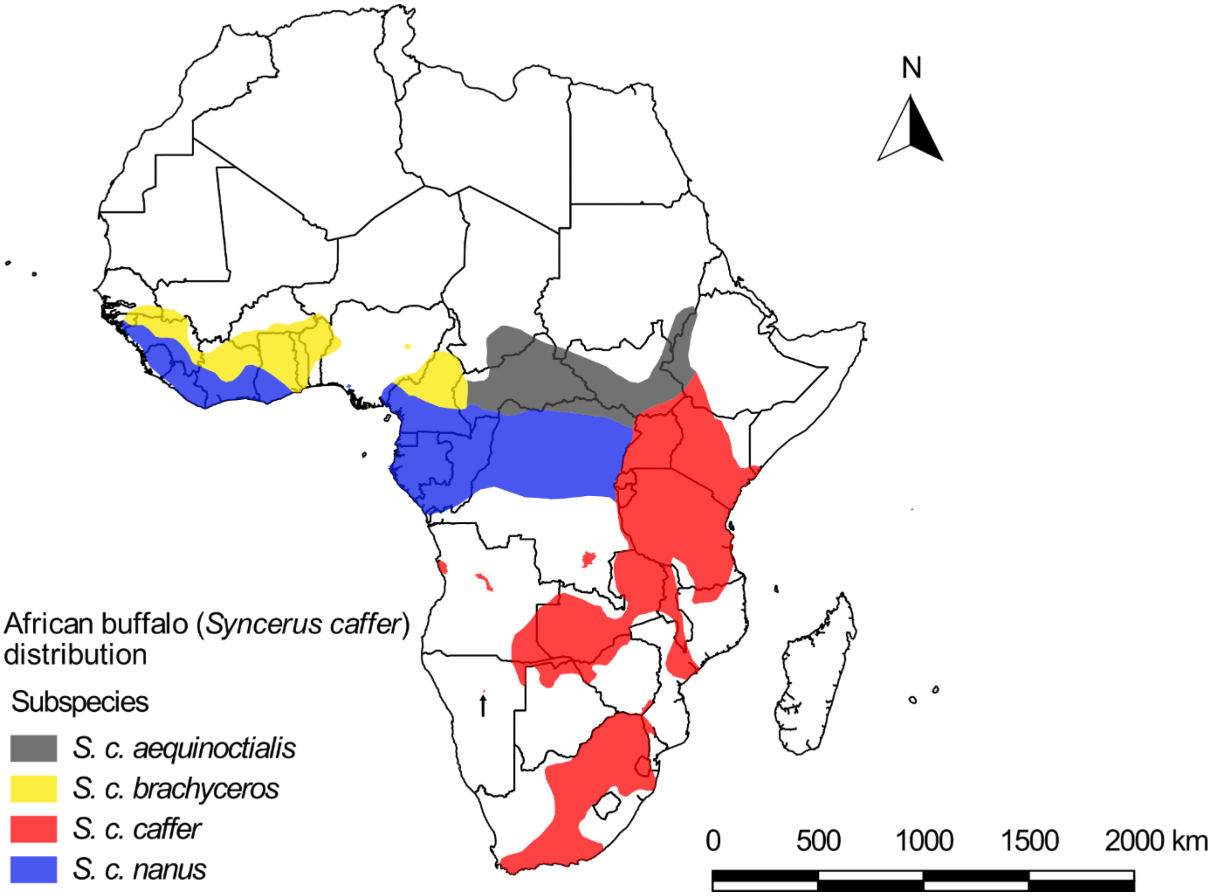


**Supplementary Fig. 7. Distribution map of African buffalo (*Syncerus caffer*) subspecies.** The arrow indicates an introduced population of Cape buffalo in Waterberg Plateau National Park, Namibia. Distribution data were obtained from the IUCN Red List database (https://www.iucnredlist.org/species/21251/50195031). The map was created in QGIS v3.4[1] (https://www.qgis.org/en/site/).

**Supplementary Table 1. Summary of sequencing report from Novogene and individual NCBI accession numbers for BioProject PRJNA645266.**

| Sample | Sampling location | Raw data (Gb) | GC Content (%) | NCBI accession |
|---|---|---|---|---|
| **A_243_14** | Addo Elephant National Park | 29.26 | 42.77 | SAMN15501057 |
| **A_251_14** | Addo Elephant National Park | 27.4 | 42.91 | SAMN15501058 |
| **A_264_14** | Addo Elephant National Park | 31 | 43.23 | SAMN15501059 |
| **A_268_14** | Addo Elephant National Park | 28.44 | 43.13 | SAMN15501060 |
| **A_87_13** | Addo Elephant National Park | 28.27 | 43.31 | SAMN15501061 |
| **B98_161** | Kruger National Park | 29.72 | 42.17 | SAMN15501062 |
| **B98_284** | Kruger National Park | 31.61 | 42.40 | SAMN15501063 |
| **B98_289** | Kruger National Park | 33.23 | 42.09 | SAMN15501064 |
| **B98_324** | Kruger National Park | 31.13 | 42.09 | SAMN15501065 |
| **B98_340** | Kruger National Park | 28.94 | 41.82 | SAMN15501066 |
| **B98_361** | Kruger National Park | 31.74 | 41.83 | SAMN15501067 |
| **B98_395** | Kruger National Park | 32.97 | 41.62 | SAMN15501068 |
| **B98_396** | Kruger National Park | 29.3 | 41.93 | SAMN15501069 |
| **B98_509** | Kruger National Park | 34.36 | 41.65 | SAMN15501070 |
| **B98_546** | Kruger National Park | 30.32 | 41.51 | SAMN15501071 |
| **B98_547** | Kruger National Park | 31.25 | 41.68 | SAMN15501072 |
| **B98_574** | Kruger National Park | 33.14 | 41.8 | SAMN15501073 |
| **B98_579** | Kruger National Park | 31.57 | 43.14 | SAMN15501074 |
| **B98_586** | Kruger National Park | 29.09 | 43.02 | SAMN15501075 |
| **B98_597** | Kruger National Park | 32.4 | 42.33 | SAMN15501076 |
| **HA_1** | Hluhluwe-iMfolozi Park | 31.1 | 42.82 | SAMN15501077 |
| **HA_41** | Hluhluwe-iMfolozi Park | 30.91 | 42.86 | SAMN15501078 |
| **HA_43** | Hluhluwe-iMfolozi Park | 29.33 | 42.77 | SAMN15501079 |
| **HB_31** | Hluhluwe-iMfolozi Park | 31.88 | 42.61 | SAMN15501080 |
| **HB_39** | Hluhluwe-iMfolozi Park | 29.32 | 42.57 | SAMN15501081 |
| **HC_11** | Hluhluwe-iMfolozi Park | 28.15 | 42.56 | SAMN15501082 |
| **HC_15** | Hluhluwe-iMfolozi Park | 29.52 | 42.37 | SAMN15501083 |
| **HC_17** | Hluhluwe-iMfolozi Park | 30.6 | 42.67 | SAMN15501084 |
| **HC_18** | Hluhluwe-iMfolozi Park | 30.99 | 42.09 | SAMN15501085 |
| **HC_20** | Hluhluwe-iMfolozi Park | 28.56 | 42.11 | SAMN15501086 |
| **HC_30** | Hluhluwe-iMfolozi Park | 31.86 | 42.47 | SAMN15501087 |
| **HC_32** | Hluhluwe-iMfolozi Park | 36.5 | 42.96 | SAMN15501088 |
| **HC_40** | Hluhluwe-iMfolozi Park | 29.78 | 42.74 | SAMN15501089 |
| **HC_41** | Hluhluwe-iMfolozi Park | 31.23 | 42.15 | SAMN15501090 |
| **HC_48** | Hluhluwe-iMfolozi Park | 32.5 | 42.52 | SAMN15501091 |
| **M_120_13** | Mokala National Park | 32.64 | 42.00 | SAMN15501092 |
| **M_12_12** | Mokala National Park | 30.8 | 42.23 | SAMN15501093 |
| **M_301_14** | Mokala National Park | 27.51 | 42.89 | SAMN15501094 |
| **M_306_14** | Mokala National Park | 26.93 | 42.84 | SAMN15501095 |
| **M_47_14** | Mokala National Park | 26.67 | 42.69 | SAMN15501096 |
| **Mean (SD)** | | **30.55 (2.09)** | **42.43 (0.49)** | |

**Supplementary Table 2. Number of sequences before and after read quality filtering and trimming.** Each row represents one sequencing lane. Some samples were sequenced across multiple lanes or multiple sequencing machines and thus appear in multiple rows

| Sample read pairs | #Raw paired sequences | #Paired sequences retained (%) | Forward only surviving (%) | Reverse only surviving (%) | #Sequences dropped (%) |
|---|---|---|---|---|---|
| **A_243_14_DSW37619_HCT32ALXX_L8** | 97 533 173 | 87 788 796 (90.01) | 7 915 259 (8.12) | 851 657 (0.87) | 977 461 (1.00) |
| **A_251_14_DSW37618_HCT32ALXX_L7** | 91 325 771 | 82 363 634 (90.19) | 7 180 128 (7.86) | 835 079 (0.91) | 946 930 (1.04) |
| **A_264_14_DSW37616_HCT32ALXX_L7** | 103 326 185 | 94 029 744 (91.00) | 7 237 136 (7.00) | 1 056 476 (1.02) | 1 002 829 (0.97) |
| **A_268_14_DSW37617_HCT32ALXX_L7** | 94 791 680 | 82 081 893 (86.59) | 10 807 890 (11.40) | 673 365 (0.71) | 1 228 532 (1.30) |
| **A_87_13_DSW37615_HCT32ALXX_L7** | 94 233 009 | 84 333 051 (89.49) | 8 020 895 (8.51) | 832 411 (0.88) | 1 046 652 (1.11) |
| **B98_161_DSW37625_HJMJMALXX_L2** | 48 942 673 | 45 009 136 (91.96) | 3 166 596 (6.47) | 338 485 (0.69) | 428 456 (0.88) |
| **B98_161_DSW37625_HJMJMALXX_L3** | 50 107 493 | 46 911 732 (93.62) | 2 559 724 (5.11) | 319 490 (0.64) | 316 547 (0.63) |
| **B98_284_DSW37626_HJMJMALXX_L2** | 50 869 584 | 46 844 232 (92.09) | 3 246 197 (6.38) | 348 159 (0.68) | 430 996 (0.85) |
| **B98_284_DSW37626_HJMJMALXX_L3** | 54 509 792 | 51 143 499 (93.82) | 2 692 956 (4.94) | 343 118 (0.63) | 330 219 (0.61) |
| **B98_289_DSW37627_HJMJMALXX_L2** | 53 090 353 | 49 389 067 (93.03) | 2 926 266 (5.51) | 376 481 (0.71) | 398 539 (0.75) |
| **B98_289_DSW37627_HJMJMALXX_L3** | 57 678 152 | 54 415 043 (94.34) | 2 557 446 (4.43) | 381 722 (0.66) | 323 941 (0.56) |
| **B98_324_DSW37628_HJMJMALXX_L2** | 49 603 030 | 45 577 523 (91.88) | 3 305 833 (6.66) | 314 237 (0.63) | 405 437 (0.82) |
| **B98_324_DSW37628_HJMJMALXX_L3** | 54 175 329 | 50 695 077 (93.58) | 2 820 210 (5.21) | 327 054 (0.60) | 332 988 (0.61) |
| **B98_340_DSW37629_HJMJMALXX_L1** | 48 219 143 | 45 193 442 (93.73) | 2 536 668 (5.26) | 230 810 (0.48) | 258 223 (0.54) |
| **B98_340_DSW37629_HJMJMALXX_L4** | 48 253 442 | 45 055 218 (93.37) | 2 604 881 (5.40) | 291 167 (0.60) | 302 176 (0.63) |

| Sample read pairs | #Raw paired sequences | #Paired sequences retained (%) | Forward only surviving (%) | Reverse only surviving (%) | #Sequences dropped (%) |
|---|---|---|---|---|---|
| **B98_361_DSW37630_HJMJMALXX_L1** | 50 884 775 | 47 864 456 (94.06) | 2 518 558 (4.95) | 238 121 (0.47) | 263 640 (0.52) |
| **B98_361_DSW37630_HJMJMALXX_L4** | 54 904 545 | 51 453 130 (93.71) | 2 793 255 (5.09) | 335 926 (0.61) | 322 234 (0.59) |
| **B98_395_DSW37631_HJMJMALXX_L1** | 55 533 629 | 52 792 974 (95.06) | 2 228 778 (4.01) | 264 591 (0.48) | 247 286 (0.45) |
| **B98_395_DSW37631_HJMJMALXX_L4** | 54 352 594 | 51 541 305 (94.83) | 2 201 629 (4.05) | 337 271 (0.62) | 272 389 (0.50) |
| **B98_396_DSW37632_HJMJMALXX_L1** | 46 512 118 | 43 832 604 (94.24) | 2 224 207 (4.78) | 218 861 (0.47) | 236 446 (0.51) |
| **B98_396_DSW37632_HJMJMALXX_L4** | 51 159 167 | 48 069 745 (93.96) | 2 478 159 (4.84) | 316 909 (0.62) | 294 354 (0.58) |
| **B98_509_DSW37633_HJMJMALXX_L1** | 59 669 150 | 56 481 893 (94.66) | 2 625 253 (4.40) | 279 387 (0.47) | 282 617 (0.47) |
| **B98_509_DSW37633_HJMJMALXX_L4** | 54 877 165 | 51 845 358 (94.48) | 2 405 529 (4.38) | 335 310 (0.61) | 290 968 (0.53) |
| **B98_546_DSW37634_HJMJMALXX_L1** | 48 766 165 | 46 123 916 (94.58) | 2 175 626 (4.46) | 228 207 (0.47) | 238 416 (0.49) |
| **B98_546_DSW37634_HJMJMALXX_L4** | 52 286 079 | 49 281 350 (94.25) | 2 393 054 (4.58) | 321 829 (0.62) | 289 846 (0.55) |
| **B98_547_DSW37635_HJMJMALXX_L3** | 52 097 331 | 48 991 406 (94.04) | 2 453 293 (4.71) | 349 813 (0.67) | 302 819 (0.58) |
| **B98_547_DSW37635_HJMJMALXX_L4** | 52 064 264 | 49 195 161 (94.49) | 2 271 495 (4.36) | 323 927 (0.62) | 273 681 (0.53) |
| **B98_574_DSW37636_HJMJMALXX_L3** | 56 336 753 | 53 029 657 (94.13) | 2 591 456 (4.60) | 386 063 (0.69) | 329 577 (0.59) |
| **B98_574_DSW37636_HJMJMALXX_L4** | 54 144 293 | 51 112 158 (94.40) | 2 383 110 (4.40) | 351 552 (0.65) | 297 473 (0.55) |
| **B98_579_DSW37637_HG5K3ALXX_L5** | 105 231 934 | 96 589 261 (91.79) | 7 068 077 (6.72) | 647 366 (0.62) | 927 230 (0.88) |
| **B98_586_DSW37638_HG5K3ALXX_L5** | 96 969 332 | 89 640 909 (92.44) | 5 875 744 (6.06) | 638 136 (0.66) | 814 543 (0.84) |

| Sample read pairs | #Raw paired sequences | #Paired sequences retained (%) | Forward only surviving (%) | Reverse only surviving (%) | #Sequences dropped (%) |
|---|---|---|---|---|---|
| **B98_597_DSW37639_HG5K3ALXX_L5** | 107 991 879 | 101 215 035 (93.72) | 5 232 495 (4.85) | 744 545 (0.69) | 799 804 (0.74) |
| **HA_1_DSW37640_HG2LKALXX_L1** | 103 658 620 | 96 376 518 (92.97) | 5 598 608 (5.40) | 806 454 (0.78) | 877 040 (0.85) |
| **HA_41_DSW37641_HG2LKALXX_L1** | 103 033 897 | 96 258 628 (93.42) | 5 175 531 (5.02) | 774 810 (0.75) | 824 928 (0.80) |
| **HA_43_DSW37642_HG2LKALXX_L4** | 97 771 592 | 90 406 297 (92.47) | 5 602 794 (5.73) | 839 587 (0.86) | 922 914 (0.94) |
| **HB_31_DSW37643_HG2LKALXX_L4** | 106 264 773 | 97 379 753 (91.64) | 6 926 439 (6.52) | 877 442 (0.83) | 1 081 139 (1.02) |
| **HB_39_DSW37644_HG2LKALXX_L4** | 97 747 948 | 88 975 121 (91.03) | 6 992 484 (7.15) | 732 297 (0.75) | 1 048 046 (1.07) |
| **HC_11_DSW37645_HG2LKALXX_L5** | 93 834 066 | 89 053 854 (94.91) | 3 545 228 (3.78) | 635 837 (0.68) | 599 147 (0.64) |
| **HC_15_DSW37646_HG2LKALXX_L5** | 98 409 683 | 92 351 308 (93.84) | 4 757 265 (4.83) | 586 152 (0.60) | 714 958 (0.73) |
| **HC_17_DSW37647_HG2LKALXX_L7** | 101 996 967 | 94 310 754 (92.46) | 5 876 710 (5.76) | 867 914 (0.85) | 941 589 (0.92) |
| **HC_18_DSW37648_HG2LKALXX_L7** | 103 294 056 | 94 595 060 (91.58) | 6 799 725 (6.58) | 855 991 (0.83) | 1 043 280 (1.01) |
| **HC_20_DSW37649_HG2LKALXX_L7** | 95 208 985 | 87 894 702 (92.32) | 5 678 820 (5.96) | 781 698 (0.82) | 853 765 (0.90) |
| **HC_30_DSW37650_HG2LKALXX_L8** | 73 421 834 | 66 575 994 (90.68) | 5 500 040 (7.49) | 525 306 (0.72) | 820 494 (1.12) |
| **HC_30_DSW37650_HGLY3ALXX_L6** | 32 794 197 | 29 825 063 (90.95) | 2 443 922 (7.45) | 200 087 (0.61) | 325 125 (0.99) |
| **HC_32_DSW37651_HCT3HALXX_L8** | 121 651 003 | 114 371 786 (94.02) | 5 675 879 (4.67) | 804 513 (0.66) | 798 825 (0.66) |
| **HC_40_DSW37652_HCT3HALXX_L6** | 80 470 337 | 75 087 671 (93.31) | 4 405 704 (5.47) | 462 972 (0.58) | 513 990 (0.64) |
| **HC_40_DSW37652_HGLY3ALXX_L6** | 18 797 213 | 17 035 796 (90.63) | 1 462 655 (7.78) | 116 155 (0.62) | 182 607 (0.97) |

| Sample read pairs | #Raw paired sequences | #Paired sequences retained (%) | Forward only surviving (%) | Reverse only surviving (%) | #Sequences dropped (%) |
|---|---|---|---|---|---|
| **HC_41_DSW37653_HJLV2ALXX_L7** | 104 092 531 | 90 758 622 (87.19) | 11 432 619 (10.98) | 640 386 (0.62) | 1 260 904 (1.21) |
| **HC_48_DSW37654_HG2LKALXX_L8** | 72 692 491 | 65 450 806 (90.04) | 6 006 404 (8.26) | 444 995 (0.61) | 790 286 (1.09) |
| **HC_48_DSW37654_HGLY3ALXX_L6** | 35 649 538 | 32 142 444 (90.16) | 2 949 865 (8.27) | 198 022 (0.56) | 359 207 (1.01) |
| **M_120_13_DSW37623_HJMJMALXX_L2** | 52 526 467 | 48 071 690 (91.52) | 3 613 215 (6.88) | 355 607 (0.68) | 485 955 (0.93) |
| **M_120_13_DSW37623_HJMJMALXX_L3** | 56 272 060 | 52 609 116 (93.49) | 2 953 811 (5.25) | 347 986 (0.62) | 361 147 (0.64) |
| **M_12_12_DSW37624_HJMJMALXX_L2** | 49 298 104 | 45 990 453 (93.29) | 2 652 419 (5.38) | 325 904 (0.66) | 329 328 (0.67) |
| **M_12_12_DSW37624_HJMJMALXX_L3** | 53 382 105 | 50 377 468 (94.37) | 2 366 625 (4.43) | 344 059 (0.64) | 293 953 (0.55) |
| **M_301_14_DSW37621_HCT32ALXX_L8** | 91 705 778 | 82 335 116 (89.78) | 7 564 765 (8.25) | 844 669 (0.92) | 961 228 (1.05) |
| **M_306_14_DSW37620_HCT32ALXX_L8** | 89 764 545 | 81 003 388 (90.24) | 7 126 699 (7.94) | 782 212 (0.87) | 852 246 (0.95) |
| **M_47_14_DSW37622_HCT32ALXX_L8** | 88 910 066 | 80 321 009 (90.34) | 6 914 488 (7.78) | 799 374 (0.90) | 875 195 (0.98) |
| **Total** | **4 073 088 838** | **3 759 449 822 (92.30)** | **251 520 517 (6.18)** | **28 787 954 (0.71)** | **33 330 545 (0.82)** |

**Supplementary Table 3. Summary of genome coverage before and after duplicates were marked (DM).** Statistics generated via the CollectWgsMetrics tool in Picard.

| Sample | Genome territory | Mean coverage | SD coverage | Exc dup (%) |
|---|---|---|---|---|
| **A_243_14** | 2612863443 | 7.72 | 5.75 | 0 |
| **A_243_14_DM** | 2612863443 | 6.86 | 5.24 | 10.84 |
| **A_251_14** | 2612863443 | 7.20 | 5.72 | 0 |
| **A_251_14_DM** | 2612863443 | 6.42 | 5.23 | 10.50 |
| **A_264_14** | 2612863443 | 7.89 | 5.94 | 0 |
| **A_264_14_DM** | 2612863443 | 6.93 | 5.34 | 11.67 |
| **A_268_14** | 2612863443 | 7.02 | 5.33 | 0 |
| **A_268_14_DM** | 2612863443 | 6.30 | 4.87 | 9.83 |
| **A_87_13** | 2612863443 | 7.22 | 5.50 | 0 |
| **A_87_13_DM** | 2612863443 | 6.43 | 5.00 | 10.64 |
| **B98_161** | 2612863443 | 8.54 | 6.10 | 0 |
| **B98_161_DM*** | 2612863443 | 6.97 | 5.13 | 17.55 |
| **B98_284** | 2612863443 | 8.96 | 6.40 | 0 |
| **B98_284_DM*** | 2612863443 | 7.28 | 5.41 | 17.95 |
| **B98_289** | 2612863443 | 9.44 | 6.57 | 0 |
| **B98_289_DM*** | 2612863443 | 7.51 | 5.41 | 19.52 |
| **B98_324** | 2612863443 | 8.82 | 6.22 | 0 |
| **B98_324_DM*** | 2612863443 | 7.08 | 5.16 | 18.79 |
| **B98_340** | 2612863443 | 8.34 | 6.13 | 0 |
| **B98_340_DM*** | 2612863443 | 6.39 | 4.89 | 22.08 |
| **B98_361** | 2612863443 | 9.18 | 6.60 | 0 |
| **B98_361_DM*** | 2612863443 | 6.93 | 5.21 | 23.12 |
| **B98_395** | 2612863443 | 9.47 | 6.84 | 0 |
| **B98_395_DM*** | 2612863443 | 7.11 | 5.41 | 23.54 |
| **B98_396** | 2612863443 | 8.49 | 6.20 | 0 |
| **B98_396_DM*** | 2612863443 | 6.50 | 4.92 | 22.16 |
| **B98_509** | 2612863443 | 10.09 | 6.91 | 0 |
| **B98_509_DM*** | 2612863443 | 7.46 | 5.31 | 24.55 |
| **B98_546** | 2612863443 | 8.92 | 6.52 | 0 |
| **B98_546_DM*** | 2612863443 | 6.77 | 5.17 | 22.72 |
| **B98_547** | 2612863443 | 9.11 | 6.58 | 0 |
| **B98_547_DM*** | 2612863443 | 7.05 | 5.30 | 21.41 |
| **B98_574** | 2612863443 | 9.54 | 6.88 | 0 |
| **B98_574_DM*** | 2612863443 | 7.34 | 5.60 | 21.69 |
| **B98_579** | 2612863443 | 8.66 | 5.94 | 0 |
| **B98_579_DM** | 2612863443 | 7.71 | 5.39 | 10.88 |
| **B98_586** | 2612863443 | 8.06 | 5.68 | 0 |
| **B98_586_DM** | 2612863443 | 7.16 | 5.14 | 11.10 |
| **B98_597** | 2612863443 | 9.13 | 6.38 | 0 |
| **B98_597_DM** | 2612863443 | 8.07 | 5.78 | 11.35 |
| **HA_1** | 2612863443 | 8.56 | 6.12 | 0 |
| **HA_1_DM** | 2612863443 | 7.49 | 5.50 | 12.30 |

| Sample | Genome territory | Mean coverage | SD coverage | Exc dup (%) |
|---|---|---|---|---|
| **HA_41** | 2612863443 | 8.61 | 6.03 | 0 |
| **HA_41_DM** | 2612863443 | 7.51 | 5.39 | 12.46 |
| **HA_43** | 2612863443 | 7.97 | 5.84 | 0 |
| **HA_43_DM** | 2612863443 | 6.95 | 5.22 | 12.46 |
| **HB_31** | 2612863443 | 8.68 | 6.16 | 0 |
| **HB_31_DM** | 2612863443 | 7.57 | 5.51 | 12.35 |
| **HB_39** | 2612863443 | 7.91 | 5.82 | 0 |
| **HB_39_DM** | 2612863443 | 6.84 | 5.16 | 13.02 |
| **HC_11** | 2612863443 | 7.77 | 5.90 | 0 |
| **HC_11_DM** | 2612863443 | 6.39 | 5.03 | 17.05 |
| **HC_15** | 2612863443 | 8.20 | 6.15 | 0 |
| **HC_15_DM** | 2612863443 | 6.76 | 5.23 | 16.94 |
| **HC_17** | 2612863443 | 8.45 | 6.11 | 0 |
| **HC_17_DM** | 2612863443 | 7.55 | 5.59 | 10.61 |
| **HC_18** | 2612863443 | 8.52 | 6.20 | 0 |
| **HC_18_DM** | 2612863443 | 7.59 | 5.67 | 10.62 |
| **HC_20** | 2612863443 | 7.92 | 5.66 | 0 |
| **HC_20_DM** | 2612863443 | 7.10 | 5.15 | 10.14 |
| **HC_30** | 2612863443 | 8.78 | 6.04 | 0 |
| **HC_30_DM‡** | 2612863443 | 7.87 | 5.51 | 10.15 |
| **HC_32** | 2612863443 | 10.32 | 6.58 | 0 |
| **HC_32_DM** | 2612863443 | 9.06 | 5.89 | 12.00 |
| **HC_40** | 2612863443 | 8.35 | 6.13 | 0 |
| **HC_40_DM‡** | 2612863443 | 7.37 | 5.58 | 11.55 |
| **HC_41** | 2612863443 | 8.11 | 5.80 | 0 |
| **HC_41_DM** | 2612863443 | 7.25 | 5.28 | 10.23 |
| **HC_48** | 2612863443 | 8.85 | 6.27 | 0 |
| **HC_48_DM‡** | 2612863443 | 7.90 | 5.73 | 10.62 |
| **M_120_13** | 2612863443 | 9.35 | 6.61 | 0 |
| **M_120_13_DM*** | 2612863443 | 7.65 | 5.64 | 17.35 |
| **M_12_12** | 2612863443 | 8.84 | 6.47 | 0 |
| **M_12_12_DM*** | 2612863443 | 7.14 | 5.45 | 18.31 |
| **M_301_14** | 2612863443 | 7.29 | 5.48 | 0 |
| **M_301_14_DM** | 2612863443 | 6.50 | 5.00 | 10.61 |
| **M_306_14** | 2612863443 | 7.17 | 5.24 | 0 |
| **M_306_14_DM** | 2612863443 | 6.37 | 4.74 | 10.91 |
| **M_47_14** | 2612863443 | 7.16 | 5.41 | 0 |
| **M_47_14_DM** | 2612863443 | 6.40 | 4.93 | 10.40 |
| **Mean coverage before DM** | | **8.47** | **6.11** | |
| **Mean coverage after DM** | | **7.14** | **5.30** | |
| **Mean read duplicates (Exc Dup)** | | | | **14.80** |

Genome territory: The number of non-N bases in the genome reference over which coverage was evaluated.

Mean coverage: The mean coverage in bases of the genome territory, after all filters were applied.
SD coverage: The standard deviation of coverage of the genome after all filters were applied.
Exc dup: The fraction of aligned bases that were filtered out because they were in reads marked as duplicates.
*Samples sequenced across two lanes of the same sequencing instrument.
‡Samples sequenced across two lanes, but on different sequencing instruments.

**Supplementary Table 4. Alignment and coverage statistics per sampling locality.** Statistics generated using the AlignmentSummaryMetrics tool from Picard.

| Sampling locality | Proportion reads aligned (SD) | Proportion HQ (Q20) reads aligned (SD) | Strand balance (SD) | Mean genome-wide coverage (mean SD) |
|---|---|---|---|---|
| **AENP** | 0.994 (0.003) | 0.909 (0.009) | 0.50 (2.42652E-05) | 6.59 (5.14) |
| **KNP** | 0.997 (0.002) | 0.921 (0.001) | 0.50 (2.15592E-05) | 7.15 (5.28) |
| **HiP** | 0.997 (0.001) | 0.919 (0.001) | 0.50 (2.94245E-05) | 7.41 (5.43) |
| **MNP** | 0.998 (0.000) | 0.919 (0.001) | 0.50 (1.29306E-05) | 6.81 (5.15) |

**Supplementary Table 5. Summary of variant metrics before and after filtering.**

| Data set | No. of SNPs | No. of INDELs | No. of multi-allelic SNPs | No. of singleton SNPs | Ti/Tv |
|---|---|---|---|---|---|
| **Raw** | 49 139 319 | 6 265 707 | 1 030 363 | 8 153 097 | 1.90 |
| **Filtered** | 3 804 535 | 0 | 0 | 0 | 2.02 |

## Supplementary Table 6. Individual genome-wide diversity indices.

| Population | Sample | Heterozygosity (-GL 1) | Heterozygosity (-GL 2) | Inbreeding (*F*) |
|---|---|---|---|---|
| **AENP** | A_243_14 | 0.002291319 | 0.002774089 | 0.208155 |
| | A_251_14 | 0.002178632 | 0.002688611 | 0.234317 |
| | A_264_14 | 0.002102339 | 0.002626992 | 0.253398 |
| | A_268_14 | 0.002283693 | 0.002855078 | 0.211124 |
| | A_87_13 | 0.002224362 | 0.002762455 | 0.226621 |
| **KNP** | B98_161 | 0.003295901 | 0.003789785 | 0.00004 |
| | B98_284 | 0.003319184 | 0.003798951 | 0.000041 |
| | B98_289 | 0.00324981 | 0.00370111 | 0.005002 |
| | B98_324 | 0.003288107 | 0.003781846 | 0.000603 |
| | B98_340 | 0.003206546 | 0.003751618 | 0.00647 |
| | B98_361 | 0.003254793 | 0.003767767 | 0.000751 |
| | B98_395 | 0.003217838 | 0.003696175 | 0.000555 |
| | B98_396 | 0.003295896 | 0.003854604 | 0.000481 |
| | B98_509 | 0.003232477 | 0.003699292 | 0.000602 |
| | B98_546 | 0.003287116 | 0.00383712 | 0.000326 |
| | B98_547 | 0.003356302 | 0.003903237 | 0.000046 |
| | B98_574 | 0.003258123 | 0.003768387 | 0.000656 |
| | B98_579 | 0.003338104 | 0.003879897 | 0.000327 |
| | B98_586 | 0.003332227 | 0.003935346 | 0.00096 |
| | B98_597 | 0.002687849 | 0.003145051 | 0.160587 |
| **HiP** | HA_1 | 0.002633009 | 0.003085595 | 0.031488 |
| | HA_41 | 0.002631377 | 0.003090141 | 0.005352 |
| | HA_43 | 0.002670355 | 0.003093391 | 0.000698 |
| | HB_31 | 0.00260152 | 0.002983572 | 0.01113 |
| | HB_39 | 0.002484253 | 0.002918803 | 0.021197 |
| | HC_11 | 0.002562633 | 0.003027731 | 0.029111 |
| | HC_15 | 0.002554905 | 0.003004638 | 0.036178 |
| | HC_17 | 0.002731626 | 0.003112482 | 0.000111 |
| | HC_18 | 0.002600692 | 0.002980888 | 0.028988 |
| | HC_20 | 0.002532242 | 0.002944969 | 0.008515 |
| | HC_30 | 0.002710954 | 0.003079401 | 0.004374 |
| | HC_32 | 0.002645481 | 0.002972926 | 0.036162 |
| | HC_40 | 0.002543342 | 0.002993296 | 0.04519 |
| | HC_41 | 0.002571964 | 0.003034663 | 0.038699 |
| | HC_48 | 0.002614778 | 0.002964487 | 0.025802 |
| **MNP** | M_120_13 | 0.003290583 | 0.003740989 | 0.000249 |
| | M_12_12 | 0.003316097 | 0.00381499 | 0.000054 |
| | M_301_14 | 0.003301199 | 0.0039377 | 0.002379 |
| | M_306_14 | 0.00324949 | 0.003881643 | 0.003451 |
| | M_47_14 | 0.003252709 | 0.003900671 | 0.002519 |

**Supplementary Table 7. Genome-wide heterozygosity of buffalo populations and other mammal species included in Fig. 2c.**

| Species | Common name | Heterozygosity | Min_Het | Max_Het | Census* | IUCNstatus** | Reference |
|---|---|---|---|---|---|---|---|
| *Syncerus caffer caffer* | MNP (Cape buffalo) | 0.003282016 | 0.00324949 | 0.003316097 | 400 | NT | This study |
| *Syncerus caffer caffer* | KNP (Cape buffalo) | 0.003241352 | 0.002687849 | 0.003356302 | 40,900 | NT | This study |
| *Syncerus caffer caffer* | Cape buffalo | 0.002880451 | 0.002102339 | 0.003356302 | 331,017 | NT | This study |
| *Syncerus caffer caffer* | HiP (Cape buffalo) | 0.002605942 | 0.002484253 | 0.002731626 | 4,000 | NT | This study |
| *Ursus arctos* | Brown bear | 0.0025 | NA | NA | 110,000 | LC | Hailer et al.[2] |
| *Syncerus caffer caffer* | AENP (Cape buffalo) | 0.002216069 | 0.002102339 | 0.002291319 | 800 | NT | This study |
| *Gorilla gorilla* | Western gorilla | 0.0019 | NA | NA | 316,000 | CR | Xue et al.[3] |
| *Papio cynocephalus* | Yellow baboon | 0.00168 | NA | NA | 1[§] | LC | Westbury et al.[4] |
| *Canis lupus* | Grey wolf | 0.00144 | 0.000459252 | 0.002176767 | 300,000 | LC | Fan et al.[5] |
| *Tursiops truncatus* | Common bottlenose dolphin | 0.00142 | NA | NA | 750,000 | LC | Yim et al.[6] |
| *Ailuropoda melanoleuca* | Giant panda | 0.00135 | NA | NA | 750 | VU | Li et al.[7] |
| *Pongo abelii* | Sumatran orangutan | 0.0012 | NA | NA | 13,846 | CR | Cho et al.[8] |
| *Panthera leo* | Lion | 0.0012 | NA | NA | 31,000 | VU | Armstrong et al.[9] |
| *Pan troglodytes* | Chimpanzee | 0.00117 | 0.0008 | 0.00176 | 236,200 | EN | Waterson et al.[10] |
| *Oryx dammah* | Scimitar-horned oryx | 0.00097 | 0.00076 | 0.0011 | 15,000 | EW | Humble et al.[11] |
| *Puma concolor* | Puma | 0.000848 | 0.00033 | 0.00166 | 10,000 | LC | Saremi et al.[12] |
| *Pan paniscus* | Bonobo | 0.0008 | 0.00065 | 0.00095 | 17,500 | EN | Prado-Martinez et al.[13] |
| *Homo sapiens* | Human (African) | 0.000791 | NA | NA | NA | NA | Westbury et al.[4] |
| *Pongo pygmaeus* | Bornean orangutan | 0.00065 | NA | NA | 104,700 | CR | Cho et al.[8] |
| *Gorilla beringei* | Eastern gorilla | 0.00065 | NA | NA | 2,600 | CR | Xue et al.[3] |
| *Balaenoptera acutorostrata* | Minke whale | 0.00061 | NA | NA | 200,000 | LC | Yim et al.[6] |
| *Panthera tigris altaica* | Siberian tiger | 0.00061 | 0.00049 | 0.00073 | 360 | EN | Cho et al.[8] |
| *Homo sapiens* | Human (European) | 0.000595 | NA | NA | NA | NA | Westbury et al.[4] |

| Species | Common name | Heterozygosity | Min_Het | Max_Het | Census* | IUCNstatus** | Reference |
|---|---|---|---|---|---|---|---|
| *Lynx lynx* | Eurasion lynx | 0.0004 | NA | NA | 8,000 | LC | Abascal et al.[14] |
| *Ursus maritimus* | Polar bear | 0.00032 | NA | NA | 26,000 | VU | Westbury et al.[4] |
| *Sarcophilus harrisii* | Tasmanian devil | 0.00032 | NA | NA | 17,500 | EN | Palkopoulou et al.[15] |
| *Gulo gulo* | Wolverine | 0.00028 | 0.00024 | 0.00032 | 580 | LC | Ekblom et al.[16] |
| *Panthera uncia* | Snow leopard | 0.00023 | NA | NA | 3,048 | EN | Cho et al.[8] |
| *Lynx pardinus* | Iberian lynx | 0.00022 | 0.00012 | 0.00022 | 156 | EN | Abascal et al.[14] |
| *Orcinus orca* | Orca | 0.000214 | NA | NA | 50,000 | DD | Westbury et al.[4] |
| *Acinonyx jubatus* | Cheetah | 0.0002 | 0.00019 | 0.00021 | 6,674 | VU | Dobrynin et al.[17] |
| *Urocyon littoralis* | Island fox | 0.000125 | 0.0000142 | 0.000408 | 4,001 | NT | Robinson et al.[18] |
| *Lipotes vexillifer* | Baiji (dolphin) | 0.000121 | NA | NA | 10 | CR | Zhou et al.[19] |
| *Hyaena brunnea* | Brown hyena | 0.000121 | NA | NA | 7,238 | NT | Westbury et al.[4] |

*Census size refers to mature individuals, if available (otherwise entire population size), as listed on the IUCN Red List website, accessed on 03/08/2020. Census size of individual populations are given for the Cape buffalo populations in this study and for the wolverine population from which the samples to calculate genome-wide heterozygosity originated.

**IUCN status is for entire distribution of species (as on 03/08/2020), not for local populations (thus the discrepancy between the status for wolverine here and in Ekblom et al.[16]).

[§]No census size for yellow baboon is available, thus a census of 1 was allocated, so it could still be included in Fig. 2c.

Cape buffalo mature individuals census size calculated as follows: Census size = 473,000*0.699 (the percentage of mature individuals- ratio as per the IUCN Red List calculation for Cape buffalo).

Scimitar-horned oryx census size obtained from Humble et al.[11].

Grey wolf census size obtained from Mech and Boitani[20].

*Homo sapiens* was not included in Fig. 2c.

**References**


1 QGIS.org. QGIS Geographic Information System. Open Source Geospatial Foundation Project. http://qgis.org. (2019).
2 Hailer, F. *et al.* Nuclear genomic sequences reveal that polar bears are an old and distinct bear lineage. *Science* **336,** 344-347, doi:10.1126/science.1216424 (2012).
3 Xue, Y. *et al.* Mountain gorilla genomes reveal the impact of long-term population decline and inbreeding. *Science* **348,** 242-245, doi:10.1126/science.aaa3952 (2015).
4 Westbury, M. V. *et al.* Extended and continuous decline in effective population size results in low genomic diversity in the world's rarest hyena species, the brown hyena. *Mol. Biol. Evol.* **35,** 1225-1237, doi:10.1093/molbev/msy037 (2018).
5 Fan, Z. *et al.* Worldwide patterns of genomic variation and admixture in gray wolves. *Genome Res.* **26,** 163-173, doi:10.1101/gr.197517.115 (2016).
6 Yim, H.-S. *et al.* Minke whale genome and aquatic adaptation in cetaceans. *Nat. Genet.* **46,** 88-92, doi:10.1038/ng.2835 (2014).
7 Li, R. *et al.* The sequence and de novo assembly of the giant panda genome. *Nature* **463,** 311-317, doi:10.1038/nature08696 (2010).
8 Cho, Y. S. *et al.* The tiger genome and comparative analysis with lion and snow leopard genomes. *Nat. Comm.* **4,** 2433, doi:10.1038/ncomms3433 (2013).
9 Armstrong, E. E. *et al.* Long live the king: Chromosome-level assembly of the lion (*Panthera leo*) using linked-read, Hi-C, and long-read data. *BMC Biol.* **18,** 3, doi:10.1186/s12915-019-0734-5 (2020).
10 Waterson, R. H., Lander, E. S., Wilson, R. K., The Chimpanzee, S. & Analysis, C. Initial sequence of the chimpanzee genome and comparison with the human genome. *Nature* **437,** 69-87, doi:10.1038/nature04072 (2005).
11 Humble, E. *et al.* Chromosomal-level genome assembly of the scimitar-horned oryx: Insights into diversity and demography of a species extinct in the wild. *Mol. Ecol. Resour.* **00,** 1-14, doi:10.1111/1755-0998.13181 (2020).
12 Saremi, N. F. *et al.* Puma genomes from North and South America provide insights into the genomic consequences of inbreeding. *Nat. Comm.* **10,** 4769, doi:10.1038/s41467-019-12741-1 (2019).
13 Prado-Martinez, J. *et al.* Great ape genetic diversity and population history. *Nature* **499,** 471-475, doi:10.1038/nature12228 (2013).
14 Abascal, F. *et al.* Extreme genomic erosion after recurrent demographic bottlenecks in the highly endangered Iberian lynx. *Genome Biol.* **17,** 251, doi:10.1186/s13059-016-1090-1 (2016).
15 Palkopoulou, E. *et al.* Complete genomes reveal signatures of demographic and genetic declines in the woolly mammoth. *Curr. Biol.* **25,** 1395-1400, doi:10.1016/j.cub.2015.04.007 (2015).
16 Ekblom, R. *et al.* Genome sequencing and conservation genomics in the Scandinavian wolverine population. *Conserv. Biol.* **32,** 1301-1312, doi:10.1111/cobi.13157 (2018).
17 Dobrynin, P. *et al.* Genomic legacy of the African cheetah, *Acinonyx jubatus*. *Genome Biol.* **16,** 277, doi:10.1186/s13059-015-0837-4 (2015).
18 Robinson, Jacqueline A. *et al.* Genomic flatlining in the endangered island fox. *Curr. Biol.* **26,** 1183-1189, doi:10.1016/j.cub.2016.02.062 (2016).
19 Zhou, X. *et al.* Baiji genomes reveal low genetic variability and new insights into secondary aquatic adaptations. *Nat. Comm.* **4,** 2708, doi:10.1038/ncomms3708 (2013).
20 Mech, L. D. & Boitani, L. *Wolves: Behavior, ecology, and conservation*. (University of Chicago Press, 2010).
